# Symbiotic nitrogen fixation for sustainable chickpea yield and prospects for genome editing in changing climatic situations

**DOI:** 10.3389/fpls.2025.1621191

**Published:** 2025-09-01

**Authors:** Rohit Kumar Mahto, Chandana B. S., Rajesh Kumar Singh, Arun Kumar, Sudhir Kumar, Renu Yadav, Debashish Dey, Aladdin Hamwieh, Rajendra Kumar

**Affiliations:** ^1^ Division of Genetics, Indian Council of Agricultural Research (ICAR)-Indian Agricultural Research Institute, New Delhi, Delhi, India; ^2^ School of Biotechnology, Institute of Science, Banaras Hindu University, Varanasi, Uttar Pradesh, India; ^3^ National Phytotron Facility, ICAR-Indian Agricultural Research Institute, New Delhi, Delhi, India; ^4^ Division of Plant Physiology, ICAR-Indian Agricultural Research Institute, New Delhi, Delhi, India; ^5^ Amity Institute of Organic Agriculture (AIOA), Amity University, Noida, India; ^6^ Crop Improvement and Molecular Biology, International Center for Agriculture Research in the Dry Areas (ICARDA), Giza, Egypt

**Keywords:** chickpea, CaNFP gene, nodulation, genome editing, sustainable agriculture

## Abstract

Chickpea (*Cicer arietinum* L.) is a vital/essential legume crop valued for its nutritional, agricultural, and economic importance, with a relatively large genome size of approximately 738 megabases. Chickpea roots establish symbiotic relationships with soil microorganisms, resulting in the formation of root nodules essential for biological nitrogen fixation. In this study, 20 chickpea genotypes were selected from a genome-wide association panel to assess nodulation traits under eight different treatment combinations involving biofertilizers (*Rhizobium*, vesicular-arbuscular mycorrhiza - VAM) and inorganic fertilizers (NPK) using a randomized block design with three replications. Pre-planting soil preparation included the application of fertilizers and biofertilizers. Comprehensive analyses including descriptive statistics, correlation, path analysis, principal component analysis, agglomerative hierarchical clustering, and gene expression studies were conducted. Among treatments, the NPK+*Rhizobium* combination significantly enhanced nodulation across genotypes, while the *Rhizobium*+VAM (T7) treatment identified ICC-9085 as a superior donor for the number of nodules, aiming for sustainable chickpea productivity. Gene expression profiling through qRT-PCR revealed that the RZ+VAM treatment notably upregulated several key genes, including CaNFP, GST, Leghemoglobin, Nodulin6, and CaLYK3, with CaNFP emerging as a pivotal regulator of nodulation. The marked upregulation of CaNFP underlines its potential as a target for enhancing symbiotic efficiency. The availability of the chickpea draft genome opens new avenues for employing genome editing tools such as CRISPR/Cas systems. Targeted editing of the CaNFP gene offers a promising strategy to improve nodule formation, nitrogen fixation, and overall plant vigor. Integrating CaNFP gene through genome editing with potential genotypes and use of microbial treatments can accelerate the development of elite chickpea cultivars, enhancing productivity while reducing reliance on chemical fertilizers and supporting sustainable agricultural practices.

## Introduction

Legumes are one of the most important crop groups for providing balanced nutrition globally. Among them, chickpea (*Cicerarietinum* L.) ranks third in global pulse production and plays a pivotal role in food security and sustainable agriculture. In addition to their dietary value, leguminous crops enhance soil fertility through symbiotic nitrogen fixation, reducing dependency on synthetic fertilizers. This symbiotic nitrogen fixation trait makes it an environmentally friendly option, especially in sustainable and low-input farming systems.

Prior to the present investigation, we conducted a comprehensive evaluation of 2,094 global chickpea core germplasm lines, including four standard checks, over two consecutive years to assess nodulation-related traits under field conditions. Based on this extensive phenotypic dataset, an association panel comprising 300 genotypes 150 high and 150 low nodulating lines was developed. From this, a focused subset of 20 genotypes (10 high and 10 low performers) was selected for detailed physiological, molecular, and transcriptomic analysis. Controlled pot experiments were conducted in triplicate at the National Phytotron Facility and the Nanaji Deshmukh Plant Phenomics Center, ICAR-IARI, New Delhi, under eight treatment regimens involving *Mesorhizobium* (IARI reference strain), vesicular-arbuscular-mycorrhiza (VAM), recommended dose of fertilizer (RDF), and their combinations. Previous phenotypic and transcriptomic analyses identified ICC 9085 as a superior and ICC 1083 as a poor nodulating genotype under both field and controlled environments (Mahto et al., 2022).

Additionally, our research group has contributed to chickpea improvement through genome editing approaches targeting cytokinin dehydrogenase genes, aimed at enhancing abiotic and biotic stress tolerance and promoting nutritional security (Mahto et al., 2023). In parallel, we have also explored post-harvest biology and gene editing potential in tomato, focusing on genotype performance, morpho-physiological and biochemical traits, and the influence of different growing media on shelf life (Yadav et al., 2024). These complementary efforts reflect our broader research objective of integrating genetic, physiological, and biotechnological tools to develop climate-resilient and high-performing crop varieties. The growth and productivity of plants are directly influenced by the availability of nutrients in the soil (Singh et al., 2022; Chandana et al., 2022; Mahto et al., 2022).

Varshney et al. (2013) initiated whole-genome sequencing and transcriptomic profiling of chickpea under drought and low phosphorus conditions, identifying key stress-responsive genes. More recent works such as Kudapa et al. (2020) utilized RNA-Seq to profile root-specific and nodulation-responsive genes in chickpea under rhizobial inoculation. The nitrogen is abundantly available in atmosphere; however, plants can only assimilate it in forms like nitrate or ammonium ions (Anas et al., 2020; Sainju et al., 2019). Nitrogen (N) is a fundamental nutrient required for plant growth and development, being a structural component of amino acids, nucleic acids, chlorophyll, and plant hormones (Tegeder and Masclaux-Daubresse, 2018). Therefore, nitrogen-based fertilizers are widely applied to improve yields. However, excessive use of these fertilizers causes severe ecological concerns, including nitrogen leaching, groundwater contamination, and eutrophication in aquatic ecosystems. Chickpeas and other legumes circumvent this dependency through a symbiotic relationship with *Rhizobium* spp., enabling biological nitrogen fixation (BNF). This process is triggered by plant-secreted flavonoids that activate *nod* genes in rhizobia (Begum et al., 2001; Hassan and Mathesius, 2012), leading to the production of Nod factors. These signaling molecules initiate nodule formation via interaction with plant receptors like LysM and LRR proteins. Inside these nodules, *Rhizobium* converts atmospheric nitrogen into plant-usable forms. Root nodules of chickpeas owing to their specialty permit *Rhizobium* to get attached to roots. Rhizobiumobtains its food from the plant and enables the usage of atmospheric nitrogen into a usable form by plants showing a symbiotic relationship. In this way, for increasing soil fertility legumes such as chickpeas are widely used as rotational crops. The process of formation of nodules in the chickpea is generally known as nodulation. Nodulation can occur due to the contribution of genes from the chickpea plants and rhizobia. The roots of chickpeas secrete certain compounds such as flavonoids and in response to that rhizobia secrete factors termed nod factors or genes (Desbrosses and Stougaard, 2011; Hayashi et al., 2013; Afonso-Grunz et al., 2014; Singh et al., 2022; Benedito et al., 2008; Libault et al., 2010). The availability of N also plays a major role in the development of nodulation and its activation in both host and symbiont (Ferguson et al., 2019). Later, genetic materials present in *Rhizobium* take part in biosynthetic machinery mechanisms to form nodulations. However, changes in gene expression or mutations can disrupt the nodulation process, potentially threatening the plant’s survival in the near future.

It is a critical biological process in which nitrogen is fixed symbiotically, enhancing nitrogen levels in the surrounding soil. The trait nodulation is directly facilitated by *Rhizobium* through the formation of nitrogen-fixing root nodules, the efficiency of this process is significantly influenced by the plant’s phosphorus (P) status a nutrient that is often limiting in many soils. Hence, the interaction effects of *Rhizobium* (RZ) and arbuscular mycorrhizal fungi (AMF/VAM) due to their complementary roles in enhancing symbiotic efficiency in the present study. AMF are known to enhance phosphorus acquisition by increasing root surface area and improving soil nutrient uptake, which indirectly promotes effective nodulation and nitrogen fixation. Moreover, AMF improve root architecture, rhizosphere health, and water absorption, further contributing to favorable conditions for rhizobial colonization and nodule development. Therefore, evaluating the combined application of RZ and AMF offers a holistic perspective on root symbiosis, enabling the identification of synergistic interactions that can be leveraged to improve nodulation traits, nutrient use efficiency, and overall plant performance under realistic agroecological conditions.

However, the efficiency of this symbiosis is highly sensitive to environmental stressors, such as temperature extremes, desiccation, drought, and heavy metal contamination (Gopalakrishnan et al., 2015). For example, nitrogen fixation significantly declines above 35°C (Kadam et al., 2019), making chickpea production vulnerable under climate change conditions. Additionally, despite advances in molecular biology, the genetic and transcriptomic regulation of nodulation in chickpea remains underexplored compared to model legumes like *Medicago truncatula.* Previous reports have also shown nodule development in legumes that have under gone comprehensive characterization (Benedito et al., 2008; Ferguson et al., 2019, 2010; Kumar et al., 2017). However, there has been relatively less focus on transcriptomic analysis of nodules and roots, particularly in the context of chickpeas. This was primarily due to the necessity of annotating these transcriptomes using genome sequences from other legumes, such as *Medicago truncatula*. With the availability of the draft genome sequence for chickpeas, we have undertaken a re-analysis of the transcriptomes of chickpea root nodules and validated the newly identified expression patterns in two extremely contradictory nodulating genotypes namely ICC-1083 (lower) and ICC-9085 (higher) employing 8 treatments in triplicates through quantitative real-time PCR (qRT-PCR).

The current study focuses on the investigation of 20 chickpea genotypes conducted in a randomized block design with three replications for assessing, dissecting and understanding nodulation features, genetics, gene expressions, impacts of various treatments and identification of high nodulating chickpea genotypes in response to the application of 8 treatments inclusive of plant growth-promoting rhizobacteria (*Rhizobium*), vesicular-arbuscular mycorrhiza (VAM), inorganic fertilizers and their combinations/interactions for harnessing genome editing sustainable yield/production.

With the recent availability of the chickpea genome, renewed efforts are being made to understand gene expression during nodulation. In this context, we re-analyzed chickpea root and nodule transcriptomes and validated expression in two contrasting genotypes ICC-1083 (low nodulation) and ICC-9085 (high nodulation) using quantitative real-time PCR (qRT-PCR) across eight treatments including *Rhizobium*, vesicular-arbuscular-mycorrhiza (VAM), inorganic fertilizers, and their combinations.

Genome editing or genetic engineering represents a pivotal advancement in modern science, involving the targeted insertion, modification, or deletion of DNA sequences. The earliest genome editing technologies were developed during the 20th century (Kiran and Chimmad, 2018). These tools function like molecular scissors, introducing site-specific cuts in the DNA. Among them, the CRISPR/Cas9 system has emerged as the most efficient and versatile platform, widely applied in the development of genetically modified organisms (GMOs) and precision genome engineering. CRISPR/Cas9 has significantly expanded the potential of agricultural research, enabling the creation of novel plant varieties with improved traits, such as acrylamide free potatoes (Halterman et al., 2015), non-browning apples, mushrooms, and potatoes, low phytic acid maize (Liang et al., 2014), blast disease-resistant rice (Wang et al., 2016), and powdery mildew-resistant wheat (Wang et al., 2014). Ongoing innovations in CRISPR technology continue to enhance its capacity for diverse genetic modifications, including gene knockouts, precise base editing, multiplex genome engineering, and the regulation of gene expression.

Despite chickpea’s ecological and nutritional significance, its full potential in sustainable agriculture remains untapped due to suboptimal and environmentally sensitive nitrogen fixation, limited understanding of its nodulation genetics, and inconsistent genotype performance across agro-ecological zones. Furthermore, climate change is exacerbating these challenges, stressing the need for genotypes that are both stress-resilient and capable of efficient nitrogen fixation.

In recent advances, several critical gaps remain in chickpea nodulation research. First, there is limited understanding of how integrated microbial treatments particularly *Rhizobium* (RZ) and vesicular arbuscularmycorrhiza (VAM) - jointly influence nodulation phenotypes and gene expression in chickpea. Second, although key regulatory genes such as *CaNFP*have been implicated in nodulation, their functional roles and underlying molecular mechanisms remain poorly characterized and largely unvalidated. Third, efforts to integrate phenotypic, genetic, and transcriptomic data for the identification of stable, high nodulating donors such as ICC 9085, are still lacking. Furthermore, the application of modern genome editing tools like CRISPR/Cas9 has not yet been fully exploited for precise manipulation of nodulation associated genes in chickpea. Although genome editing in chickpea is still in the early phases due to transformation constraints, Badhan et al. (2021) reported early success using CRISPR-Cas9 in targeting stress-responsive transcription factors, setting a precedent for functional genomics applications in legumes.

By systematically addressing these research gaps, the present study aims to lay a robust foundation for microbe-assisted and genome editing–enabled strategies to enhance symbiotic nitrogen fixation, stress resilience, and sustainable yield in chickpea. This research study is important as some modification need to enhance nitrogen fixation efficiency in chickpea, a legume of global significance for both food security and soil health. In the face of increasing climate variability, improving the symbiotic interaction between chickpea and nitrogen-fixing bacteria is essential for achieving sustainable and low input agricultural systems. The research explores how genotype x environment x treatment interactions affect nodulation, offering insights into the molecular and physiological basis of this complex trait. By identifying and characterizing high and low nodulating genotypes under different microbial and nutrient treatments, the study provides a foundation for breeding and biotechnological interventions aimed at improving nitrogen use efficiency. Furthermore, it lays the groundwork for the strategic application of genome editing as a supportive tool to regulate the expression of key genes governing nodulation and stress resilience.

## Materials and methods

### Experimental conditions, plant materials, and treatments

The experimental materials used for the study consisted of 20 chickpea genotypes including two checks *Desi* (Pusa 372) and *Kabuli* (Pusa 3022), out of which 18 were selected based on phenotypic data recorded on a large germplasm (2094) inclusive of global core germplasm procured from ICRISAT, Patencheru, Telangana, India and breeding materials developed by ICAR-Indian Agricultural Research Institute (IARI), Pusa, New Delhi, India (data not published). Twenty genotypes included nine genotypes with extremely low and nine with extremely high numbers of nodules (**Table 1**).

**Table 1:** List of genotypes with high and low number of nodules and their country of origin.

S. no	Low nod. genotype	Location	S. no	High nod. genotypes	Location
1	ICC-1083	Iran	11	ICC-1891	India
2	ICC-1172	India	12	ICC-1896	India
3	ICC-3093	Iran	13	ICC-2083	Mexico
4	ICC-3631	Iran	14	ICC-3696	Iran
5	ICC-6579	Iran	15	ICC-4638	India
6	ICC-6995	Iran	16	ICC-6661	Iran
7	ICC-7167	Turkey	17	ICC-9002	Iran
8	ICC-7305	Afghanistan	18	ICC-9085	Iran
9	ICC-13185	Iran	19	BG-372 **(Check)**	India
10	ICC-1852	India	20	BG-3022 **(Check)**	India

Bold values are Checks used in this experiment.

Experiments were conducted over two consecutive years (2020-21 & 2021-22) in pots, which were filled with the soil taken from the experimental fields No. 6B & 6C of the Division of Genetics, IARI, situated at (29.7008° N Latitude, 76.9839° E Longitude, 228.6 m AMSL) for the respective years. Before the application of the treatments, soil analyses were performed during both years for soil samples those were used as controls to assess spatial variability for key soil parameters (**Supplementary Table S1**). The classification of soil fertility status in the current study is based on standardized value ranges aligned with established agronomic thresholds. These thresholds were used to categorize soil nutrient levels into low, medium, and high fertility classes. This range-based classification facilitates consistent interpretation across treatment groups and geographical locations and avoids the complexity of presenting exhaustive raw soil data. The pH levels ranged from 6.7 to 9.1, with a mean of 7.9, indicating mildly alkaline soil. Electrical conductivity (EC) values for soil salinity ranged from 0.08 to 1.03, suggesting low salinity levels. Organic carbon (OC) content in the 0–15 cm depth varied from 0.055 to 1.11, indicating moderate variability. Nitrogen (N) content ranged from 132 to 514, suggesting moderate to high availability. Phosphorus (P) levels ranged from 3.1 to 254, reflecting varying phosphorus availability. Potassium (K) content ranged from 98 to 1879, showing significant spatial variability. Sulfur (S) levels varied from 2.8 to 35.8, indicating diverse sulfur availability. Micronutrient levels, including zinc (Zn), iron (Fe), manganese (Mn), and copper (Cu), exhibited spatial variability, suggesting diverse availability across the farms. This spatial variability underscores the importance of targeted management practices and crop improvement strategies to optimize agricultural productivity in different treatment conditions and areas of the experimental farms. Seed germination percentage was evaluated prior to sowing to ensure uniform plant establishment. Soil physic-chemical properties, including pH, electrical conductivity (EC), organic carbon, nitrogen (N), phosphorus (P), potassium (K), sulfur (S), and micronutrients (Zn, Fe, Mn, Cu), were measured and reported using appropriate SI units (e.g., mg/kg, dS/m, kg/ha). Microbial inoculum concentrations were clearly defined. *Mesorhizobium ciceri* (IARI reference strain) was applied at 1 × 10^8^ CFU/mL, while vesicular arbuscular-mycorrhiza (VAM) inoculum was administered at 100 spores per gram of carrier material, both standardized according to established protocols to ensure effective root colonization. The sterile distilled water was used for all experimental procedures and sample processing to prevent microbial contamination, while running tap water was used for irrigation to maintain adequate soil moisture. The explicitly well described experiment was conducted in RBD design that included 8 treatment combinations (T1–T8), 20 chickpea genotypes under 3 replications, totaling 480 pots. Environmental parameters such as temperature, humidity, precipitation, sunshine hours, and evaporation rates were recorded for both cropping seasons to provide climatic context. Additionally, pest and disease monitoring were conducted through weekly visual inspections, and no significant biotic stress incidences were recorded. All growth, nodulation, physiological, and yield-related traits were measured using standardized methods and units, ensuring consistency and replicability of results. Prior to transplantation, seed germination tests were conducted under controlled conditions, and the germination percentage across all 20 genotypes ranged from 92% to 98%, indicating high seed viability and uniform establishment.

The weather data for the 2020–21 crop season indicated a temperate climate with an average temperature of 20.03°C, ranging from a minimum of 2.52 to a maximum of 42.81°C. Total rainfall during the period was 13.11 mm, and the peak wind speed was recorded as 6.68 kmph. Wind direction values were predominantly 3.71, with relative humidity (RH) levels reaching to a maximum of 309.00% and 286.00% for RH-I and RH-II, respectively. The bright sunshine for 6.02 hours with 2.07 mm measured evaporation was observed (**Supplementary Table S2**). However, the crop season 2021–22 indicated a temperate climate characterized by an average temperature of 21.68°C, spanning from a minimum of 2.4°C to a maximum of 30.9°C. Recorded precipitation reached to 69.2 mm, with the highest wind speed peaking at 9.5 kmph. Elevated relative humidity attaining 100% along with a restricted duration of bright sunshine hours (4.20 hrs) and 5.1 mm evaporation were recorded (**Supplementary Table S2**).

The experimental plant materials were grown in triplicates in 16-inch diameter pots under varied treatment (**Table 2**) combinations as T1(Control-No fertilizer, No inoculants), T2 {required dose of fertilizer (RDF) as DAP 100 Kg/ha equivalent to 20KgN and 40 Kg P}, T3 {*Meso rhizobium*(Mr) Ref strain (IARI)}, T4 {Vesicular Arbuscular Mycorrhiza (VAM)}, T5(VAM+RDF), T6{Mr (ref strain IARI+RDF)}, T7{Mr (Ref strain IARI)+VAM}, and T8{Mr (Ref strain IARI)+VAM+RDF}. All plants were systematically monitored for any signs of pest or disease infestation. Weekly visual inspections were carried out, paying close attention to morphological symptoms such as leaf chlorosis, necrotic spots, wilting, stunted growth, or any abnormal tissue development. Upon detection of any irregularities, standard phyto-pathological diagnostic protocols were promptly implemented to identify potential pathogens or insect pests. These included the use of hand lenses for closer inspection and sample collection for laboratory verification, when necessary. Despite consistent monitoring, no significant incidence of pests or disease was recorded during the experimental duration, indicating a healthy crop stand under the adopted management practices and controlled conditions. Thus, a total of 20 genotypes × 8 treatments × 3 replications = 480 pots were used for the experiment. Each pot initially contained 9 plants (3 plants for observations on root/shoot traits, 3 plants for RNA isolation those were uprooted after 60 days of sowing (50% flowering) and the remaining 3 plants were harvested at the maturity (110 days) stages for pods and seed-related data observations. The pots were placed at the premises of the Nanaji Deshmukh National Phenomic Facility (NDNPF), ICARI-IARI, Pusa, New Delhi, India, and plants were grown under natural light conditions with a temperature range of 32°C/28°C (day/night) and 70 to 80% RH. Plants were grown at saturated moisture conditions (25%V/V basis) under well watering systems. Recommended weed, pest, and disease control measures were practiced.

**Table 2:** Detailed list of treatments name and their combinations.

Treatment code	Abbreviation	Description
T1	C	Control (no inoculation, no fertilizer)
T2	RZ	*Rhizobium* only
T3	VAM	Vesicular-Arbuscular- Mycorrhiza only
T4	RDF	Recommended Dose of Fertilizer only
T5	RZ+VAM	*Rhizobium* + VAM
T6	RZ+RDF	*Rhizobium* + Recommended Dose of Fertilizer
T7	VAM+RDF	VAM + Recommended Dose of Fertilizer
T8	RZ+VAM+RDF	*Rhizobium* + VAM + Recommended Dose of Fertilizer

### Seed inoculation with *Rhizobium* and application of VAM

During *Rhizobium* treatment visually uniform seeds were selected to nullify the effect on plant growth due to differential seed vigor. In commercial use, a preparation of 200 g of *Rhizobium* is used for treating 10 kg seeds, accordingly, for treating 100 seed weight 1.2-1.5 g *Rhizobium* was used. During *Rhizobium* treatments, seeds were mixed with CMC solution followed by a sprinkling of *Rhizobium* (F-75 strain) culture, and seeds were dried under shade for immediate sowing. VAM applications were made at the time of sowing by making a hole at a depth of 5 cm and by placing the VAM(mixed inoculum of *Funneliformis mosseae*, *Rhizoglomus fasciculatum*, *Entrophospora etunicata*, *Gigsporamargarita*, and *Scutella* sp*ora* species) followed by placing of seeds and covering with soil. commonly used for their colonization potential in legumes. However, as only one mixed inoculum was used, comparing the performance of individual species was beyond the scope of this study. Despite the absence of colonization data, we observed notable phenotypic responses under Treatment T4 (VAM alone) and T7 (*Rhizobium* + VAM), where T4 showed increased root biomass and moderate nodulation, while T7 demonstrated synergistic effects in terms of nodule number, plant biomass, and upregulation of nodulation-related genes such as *CaNFP*, *Leghemoglobin*, and *Nodulin6*. These responses likely reflect successful VAM establishment, albeit not directly confirmed. To strengthen future work, we have indicated in the revised manuscript that subsequent experiments will include detailed VAM colonization analysis using established methods such as Trypan Blue staining and light microscopy (Phillips & Hayman, 1970) to quantify root colonization and structures like hyphae, vesicles, and arbuscules. Finally, the revised *Discussion* also offers a clearer interpretation of the differential effects of VAM alone (T4), VAM + *Rhizobium* (T7), and the tripartite combination with RDF (T8), though we agree that without colonization data, conclusions on VAM performance under each condition remain preliminary.

Sterile distilled water was employed for all critical experimental procedures, including the preparation of *Mesorhizobium* and VAM inoculums, RNA isolation, biochemical assays, and nitrogen estimation, in order to avoid microbial contamination and ensure the reproducibility of results. For irrigation and routine maintenance of the experimental plants, running tap water was used. This differentiation between water types was made intentionally to uphold the sterility and integrity of lab-based processes while also maintaining realistic field-like growing conditions for the chickpea genotypes. By documenting these details, we aimed to enhance the reliability, accuracy, and transparency of the experimental design and outcomes.

Phenotyping of diverse 20 chickpea genotypes of the subset for nodulation-associated traits namely the number of nodules, nodules fresh weight (mg), nodules dry weight (mg), root fresh weight (g), root dry weight (g), shoot fresh weight (g), shoot dry weight (g), plant height (cm), days to maturity, days to flowering, number of primary branches, number of secondary branches, number of pods/plant, number of seeds per plant, and seed weight/plant (g) were accomplished during the period either at the stage of at least 50% flowering of plants or after 55–65 days of sowing or at the time of harvesting as per the requirement of the trait under observation. For example, as nodule formation usually starts after 45 days of sowing, to take observations on nodulating traits after 55–65 days of sowing one day before the root traits’ observation process, selected pots were watered, and plants were uprooted carefully with the help of sharp edge objects followed by thorough washing with water and data was recorded. The mean of three observations was computed for all the measurements.

### Fluorescence imaging for chlorophyll content

Fluorescence imaging for assessing the effects of various combinations of treatments on the chlorophyll contents of the genotypes was performed utilizing an automated and high throughput infrastructure established and nomenclature as Nanaji Deshmukh National Phenomic Facility (NDNPF), IARI-IARI, Pusa, New Delhi, India. Crop Reporter™ (PhenoVation, Netherlands) phenotyping machine having a pixel resolution of 1038x1038 which captures spatial heterogeneity of energy quenched by fluorescence was used (Kumar et al., 2017). Samples were loaded on the RFID (Radiofrequency enabled ID) based cart. All samples were dark-adapted for 15 minutes to completely close the electron transport chain. Afterward, crop reporter-based LED (Light emitting diode) induced direct fluorescence imaging technology was used to image the samples. The Crop Reporter captured spectral images from the top view by using a 6-position optical filter wheel. For color, it used red, green, and blue information. The software reconstructed the images into a 3x14 bit color image with a spatial resolution of 1.4 MP. A total of 25 frames were captured. Image processing was done using Lemna grid software i.e. started from de-mosaicing followed by foreground separation using image thresholding. Once the noises and background were removed, the image-based analysis was done to convert the pixel-based information into grey values. Out of the 25 frames, the first frame was considered as F_0_ while frame F_13_ was considered as Fm, and F_14_ to F_24_ frames were not considered for our study. So, based on these values FV/Fm was calculated. The first plants were exposed to low-intensity actinic light in the red region of the spectrum (0.5 μmol m^− 2^s^− 1^, wavelength 650 nm) which gave minimum fluorescence (F_0_). Then a white saturating pulse (2800 μmol m^− 2^s^− 1^) was flashed to image maximum fluorescence (Fm) with peaks around 650 nm and 700 nm. The image was captured in a far-red region (700 nm) to ensure that the captured signal did not overlap with the wavelength of provided actinic light. The configuration and timing of the pulse as well as image acquisition were controlled using the imager configuration module of Lemna Control software (LemnaTech, Germany). Images were acquired in top view configuration at regular intervals through the significant stages of the chickpea. The top view configuration was selected because the top leaves have high assimilation rates as well as higher photosynthesis efficiency than the middle or bottom leaves. The acquired ChlF image data was further analyzed for various fluorescence-based photosynthetic parameters as minimum and maximum fluorescence.

### Estimation of nitrogen

Seed nitrogen estimations were done for all the selected genotypes and treatment combinations according to Kjeldahl’s method (AOAC, 1990). The percentage of total nitrogen (N) in the samples was determined following the procedures outlined in the AOAC 1990 guidelines. Initially, 0.5 g of the finely ground powder of the samples was passed through a 40 mm sieve to ensure uniform particle size. Subsequently, the prepared samples were transferred into a 500 mL Kjeldahl flask.

While initiating the digestion process, 25 mL of concentrated sulfuric acid (H_2_SO_4_) was added into the flask, and the mixture was left undisturbed for 30 minutes. After this resting period of 30 minutes, 5 g of sodium thiosulfate was introduced into the mixture, followed by 30-minute incubation. Then the catalyst mixture was transferred into the digestion system. In this experiment the Kjeldahl flask was gradually heated until persistent frothing was observed. At this stage, the heat was increased, and digestion continued for an additional 30 minutes, ensuring the digests became clear. Once digestion was complete, the flask was allowed to cool. Next to cooling of the flask, 150 mL of water was carefully added, and the solution was cooled further. For distillation, 120 mL of a 40% sodium hydroxide (NaOH) solution was gently poured along the inner walls of the flask. Simultaneously, glass beads and drops of mineral oil were added. The flask was then connected to a distillation setup. During distillation, the released ammonia gas was captured in a solution containing 25 mL of boric acid, which included a mixed indicator of methyl red and methylene blue. Distillation proceeded until 150 mL of distillate was collected. The ammonia present in the distillate was then titrated using a 0.1 N H_2_SO_4_ solution. This procedure allowed for the precise determination of the total nitrogen content within the analyzed sample utilizing the following formula,


$$\begin{array}{c} \text{Nitrogen }\left(\%\right)\;\\ =\;\frac{\text{Titre value }\left({\text{V}}_{\text{a}}-{\text{V}}_{\text{b}}\right)\times \text{normality of HCl }\times 14.001\;}{\text{W }\left(\text{g}\right)}\times 100 \end{array}$$

Where: Va= Titre value of sample, Vb= Titre value of blank, W= Weight of sample taken for digestion (0.5g).

### Gene expression profiling

For expression profiling, two genotypes with contrasting responses for the aforesaid treatments were selected. Freshly harvested root samples were collected and stored in liquid nitrogen for future use. RNA extraction from root tissues was carried out utilizing the Trizol method. 50–100 mg ground powder of each sample was rapidly pulverized in liquid nitrogen followed by the addition of 1 ml Trizol and incubation for 5 minutes at room temperature (RT). Subsequently, 200 μl chloroform per 1 ml Trizol was vigorously mixed for 15 seconds, and the mixture was incubated at RT for 10–15 minutes. The resulting tubes underwent centrifugation at 12,000 rpm for 15 minutes at 4°C. The aqueous phase was carefully transferred to a new tube, and 600 μl isopropyl alcohol was added. After a 10-minute incubation at RT, the samples were centrifuged again at 12,000 rpm for 15 minutes at 4°C. The resulting pellets were washed with 0.5 ml 70% ethanol, followed by another centrifugation at 7500 rpm for 5 minutes at 4°C. This washing step was repeated thrice. The pellet was air-dried overnight and subsequently dissolved in an appropriate volume of RNase-free water with incubation at 55-60°C for 10 minutes. RNA quantification was performed using a Thermo Nanodrop 2000c spectrophotometer, and purity was confirmed by assessing the A260/A280 ratio. To get rid of DNA contamination, total RNA was treated with DNAse (Thermo Scientific), and polymerase chain reaction (PCR) was accomplished followed by a PCR run on the 1.5% denaturing agarose gel in order to check the RNA quality. The cDNA preparation was done using SuperScript^®^ III First-Strand Synthesis followed by a cDNA reverse transcription kit (Thermo Fisher, USA). For expression profiling *Applied Biosystems™ SYBR Green* Master Mix was used in the AriaMx Real-time PCR System (Agilent, USA). For the calculation of relative expressions, the Delta Ct method was utilized.

### Statistical analysis

To assess the variations in parameters under the treatments, a one-way ANOVA, Genetic Parameters, Correlation, Path, and Diversity were performed using WindoStat 9.3V. The *P value*>0.05 is regarded as statistically significant, and the data is represented as mean ± SE. The genotypic and phenotypic coefficients of variations were determined following the methodology proposed by Burton and Devane (Burton and Devane, 1953). These coefficients were subsequently classified into three categories low (< 10%), moderate (10-20%), and high (> 20%), following the criteria outlined by Sivasubramanian and Menon (1973). Broad-sense heritability as described by Allard (Allard, 1999). was computed and categorized as low (0-30%), medium (31-61%), and high (61-100%), following the guidelines provided by Robinson et al (Robinson et al., 1949). The anticipated genetic advances, expressed as a percentage of the mean, were calculated and classified into three groups as low (< 10%), moderate (10-20%), and high (> 20%), as per the recommendations of (Johnson et al., 1955). Analysis of variance was analyzed as per (Panse and Sukhatme, 1978). Genotypic (rg) and phenotypic (rp) correlation coefficients were computed using the procedure elucidated by (Miller et al., 1959). Path analysis was performed following the approach advocated by (Wright, 1921). and (Dewey and Lu, 1959). Diversity analysis was executed utilizing Mahalanobis D2 statistics (Mahalanobis, 1936), and subsequently, the genotypes were clustered into distinct groups based on Tocher’s method as described by Rao (Rao, 1952). Further, the data was subjected to analysis for various components of principal component analysis (PCA-I, II & III based on X, Y& Z vectors) using XLStat2020 V. The X vector represents the PCA- I, a linear combination of the original variables (features) that capture the maximum variance, essentially a direction in the feature space along the most varied data. Y vector represents the PCA-II which is orthogonal (perpendicular) to the first principal component and captures the second most significant source of variance in the data or in other words, captures the remaining variance that was not explained by the first principal component. Z vector represents the third principal component which is orthogonal to both X and Y vectors and captures the third most significant source of variance in the data.

## Results and discussions

With the advent of genome editing technologies, chickpea (*Cicerarietinum* L.) improvement has entered a new era of precision breeding, enabling targeted modifications to enhance agronomic traits such as nodulation, nitrogen fixation, and yield stability. In this context, the present investigation was undertaken to study latent potentials of variability, heritability, genetic advance, correlations, direct and indirect effects, diversity, and gene expressions among 20 diverse chickpea genotypes, traits, and treatments. The use of value ranges rather than individual soil test values allows for a clearer and more meaningful comparison of fertility status across treatments and locations. This approach is consistent with national and international soil fertility interpretation guidelines, which define critical limits for nutrient sufficiency and crop response. Such categorization supports effective nutrient management and is widely accepted in agronomic studies (Brady and Weil, 2016).

Analysis of variance revealed extremely significant variances for all the parameters, genotypes, and treatments, highlighting a broad range of diversity and confirming the appropriateness of the experimental data. This analysis indicated a significant degree of diversity across all characters, suggesting the potential for effective selection and substantial genetic improvement (**Supplementary Table S3**). However, the analysis of variance alone is insufficient to fully explain inherent genetic variability. Therefore, to clearly distinguish the genetic component from the observed phenotypic variance, the phenotypic and genotypic coefficients of variation were calculated, representing the extent of variability for particular traits. The study examined 15 traits, 8 treatments, 20 genotypes, and their interactions. Significant variations were observed among traits, replications, years, treatments, genotypes, and their interactions at p< 0.001. Coefficients of variation ranged from 5.74% to 8.23%, suggesting moderate to low variability within the traits studied. Notably, traits such as the number of nodules, nodule fresh weight, seed weight per plant, and days to maturity exhibited highly significant variations across genotypes and treatments under ambient conditions, reflecting substantial genetic and environmental influences. In this study, a focused subset of 20 chickpea genotypes was selected from a broader association panel previously developed from 2,094 global accessions. This subset included 18 genotypes representing two distinct nodulation categories: nine high nodulating and nine extremely high nodulating lines, based on rigorous multi-environment phenotypic evaluations, with two standard checks which is BG 3022 is and BG-372. The genotype ICC-9085 exhibited superior performance, characterized by a high number of nodules and enhanced yield, whereas ICC-1083 consistently showed poor performance with minimal nodulation and low yield outcomes. These two genotypes have been strategically highlighted throughout the manuscript for comparative validation across phenotypic, biochemical, and molecular analyses. The inclusion of clearly defined nodulation categories ensures coherence and enhances interpretability across all experimental sections, addressing previous inconsistencies in genotype classification and strengthening the overall narrative.

### Variability

The presence of sufficient genetic heterogeneity is a basic prerequisite for carrying out any crop enhancement effort. Thus, it is important to select the genotypes based on heritability and genetic advancement since selection based on phenotypic performance may be ineffective because these genotypes may perform poorly in later segregating generations. The genotypic coefficient of variation (GCV) quantifies the extent of genetic variability in the population, heritability (h^2^) quantifies the heritable portion of variability, and the genetic advance (GA) indicates the expected desirable genetic gain expressed as genetic advance as percent mean (GAM) after selection. The comprehensive estimations for PCV, GCV, and heritability in a broad sense along with their GAM at 5% for all the genotypes revealed that generally, PCV values were higher than their respective GCV values indicating slight environmental influences (**Supplementary Table S4**).

**Figure 1:**
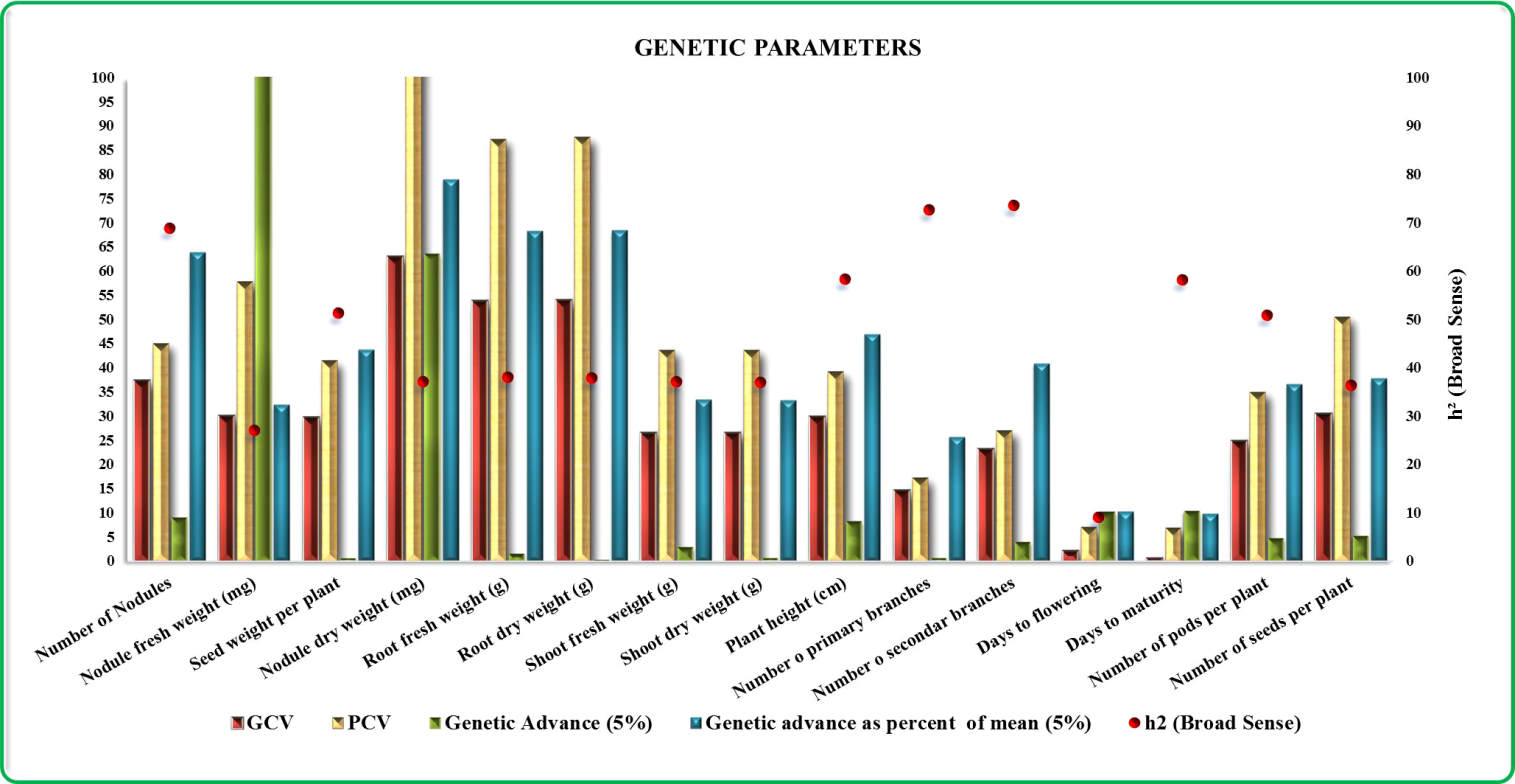
Diagrammatic representations illustrating the variability in genetic parameters for all 15 traits. The color keys denote brown=GCV, yellow=PCV, green=genetic advance, blue=genetic advance at 5%, and red dots=broad-sense heritability.

Among the traits examined, the nodules’ dry weight (mg) exhibited the highest GCV (63.0), followed by root dry weight (54.0 g) and root fresh weight (53.8 g). Notably, the number of nodules emerged as a significant parameter with a GCV of 37.4%. Similarly, the number of seeds per plant demonstrated substantial genetic variability with a GCV of 30.4%, whereas days to flowering and days to maturity showcased lower GCV values of 2.1% and 0.6%, respectively. Moving to PCV, nodules’ dry weight displayed the highest PCV (103.5%), followed by root dry weight (87.7%) and root fresh weight (87.3%). Nodule fresh weight demonstrated considerable phenotypic variability with a PCV of 57.8%, closely followed by the number of seeds per plant (50.5%). Additionally, h^2^ analyses unveiled that the number of secondary branches held the highest h^2^ value (73.6%), followed by the number of primary branches (72.6%) and the number of nodules (68.9%). Meanwhile, the GAM (5%) revealed that nodule fresh weight manifested the greatest advancement (107.8%), while the number of nodules exhibited was the lowest (9.0%). GAM was pronouncedly notable for nodule dry weight (79.1%), followed by root dry weight (68.5%) and root fresh weight (68.3%). The traits days to flowering and days to maturity showcased comparatively lower GAM values of 10.3% and 9.7%, respectively. This comprehensive analysis of various traits provides insights into their variability, heritability, and potential for genetic improvement (**Figure 1**).

### Correlation studies

**Figure 2:**
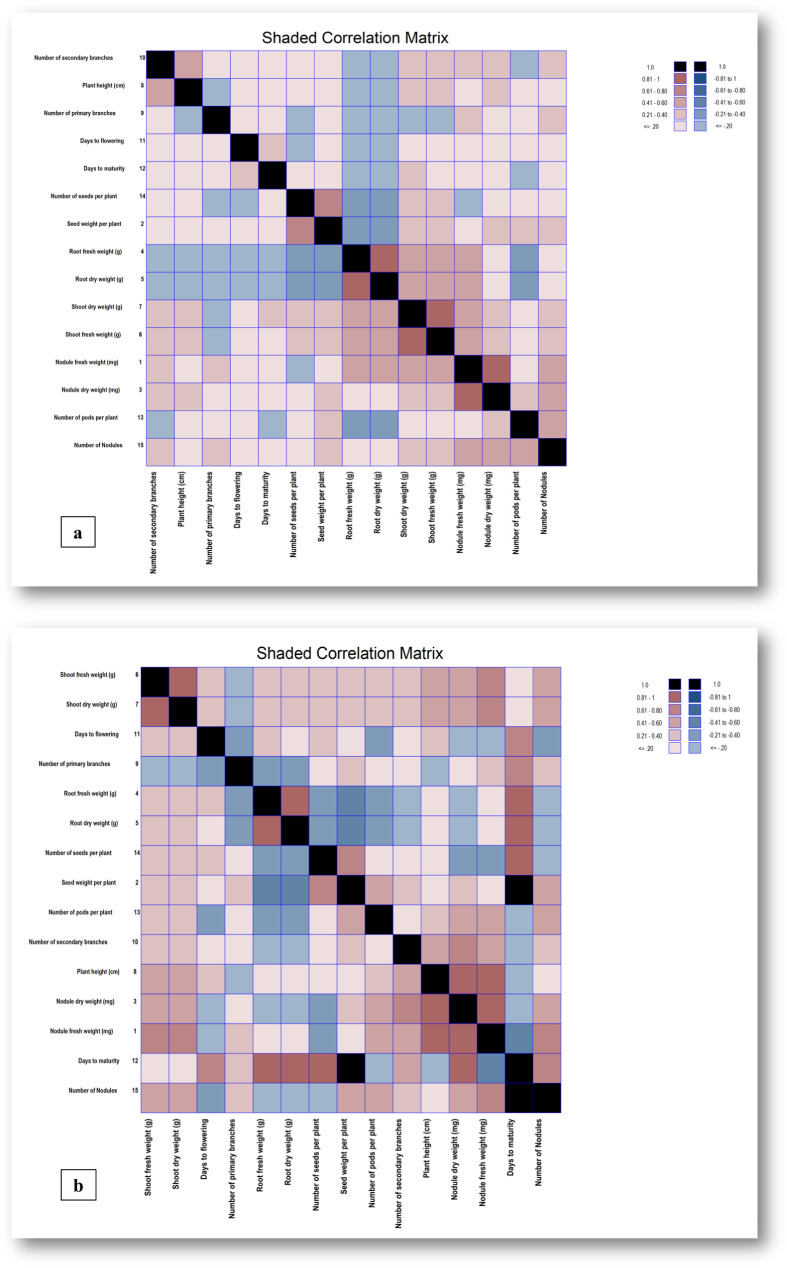
**(a, b)** Phenotypic and Genotypic correlation matrices depicted as shaded heat maps for yield and its 15 component traits. The heat map assigns colors to represent values, with black indicating 1.0, Redwood red for values between 0.81 and 1, Indian Red for values between 0.61 and 0.80, Piglet pink for values between 0.41 and 0.60, Grapefruit dark for values between 0.21 and 0.40, and Grapefruit light for values less than or equal to 0.20. On the negative side, Dark black represents -1.0, Dark slate blue represents values between -0.81 and -1, Light slate blue represents values between -0.61 and -0.80, Dodger blue represents values between -0.41 and -0.60, Steel blue dark represents values between -0.21 and -0.40, and Steel light blue represents values less than or equal to -0.20.

Correlation studies help to understand the interrelationships among yield and related traits. Association between two different traits is the result of linkage and pleiotropic effect of genes. Therefore, to identify suitable selection strategies for improvement in yield, it is essential to know about the correlation between yield and yield component traits. The phenotypic and genotypic correlation coefficients computed among the fifteen characters have been presented (**Figures 2a, b**).

The phenotypic correlation matrix elucidates an expressible degree of intricate relationships among diverse traits within chickpea genotypes. Notable correlations were observed between various traits at both phenotypic and genotypic levels (**Supplementary Table S5a, b**). Nodule fresh weight exhibited significant positive phenotypic correlations with nodule dry weight (0.868), shoot dry weight (0.520), shoot fresh weight (0.518), number of nodules (0.427), root dry weight (0.411), root fresh weight (0.404) and non-significant positive correlations with number of primary branches (0.236), number of secondary branches (0.206), number of pods per plant (0.175), days to maturity (0.167), plant height (0.136), and days to flowering (0.065), along with non-significant negative correlation with the number of seeds per plant (-0.059). On the other hand, the nodule fresh weight demonstrated significant genotypic correlations with plant height (0.917), nodule dry weight (0.848), shoot fresh weight (0.745), shoot dry weight (0.743), number of nodules (0.637), and number of secondary branches (0.497), while showing non-significant positive correlations with root fresh weight (0.144), root dry weight (0.141), seed weight per plant (0.139), and non-significant negative correlations with number of seeds per plant (-0.335) and days to maturity (-0.568). The trait seed weight per plant exhibited significant positive phenotypic correlations with number of seeds per plant (0.695), shoot dry weight (0.356), shoot fresh weight (0.355); non-significant positive correlations with nodule dry weight (0.212), number of nodules (0.202), days to maturity (0.164), and non-significant negative correlations with root fresh weight (-0.317) and root dry weight (-0.319). Nodule dry weight indicated significant positive phenotypic correlations with nodule fresh weight (0.400), number of pods per plant (0.381), seed weight per plant (0.378); shoot fresh weight (0.497), days to maturity (0.255), and plant height (0.181), among others. Root fresh weight exhibited significant positive correlations with root dry weight (0.983), shoot dry weight (0.419), and shoot fresh weight (0.418); non-significant negative correlations with root fresh weight (-0.175) and number of seeds per plant (-0.23). The number of primary branches displayed non-significant correlations with various traits, while the number of secondary branches showed significant positive correlations with plant height (0.477), shoot fresh weight (0.326), shoot dry weight (0.320), and non-significant positive correlations with nodule dry weight (0.249), number of nodules (0.226), and nodule fresh weight (0.206). Days to flowering demonstrated significant positive correlations with days to maturity (0.285) and significant negative correlations with days to flowering (-0.352) at both phenotypic and genotypic levels. The obtained results align with the findings of several others (Elias, 2009; Mohamed and Hassan, 2015 and Hazra et al., 2021). In the current investigation, the genotypic correlations for most of the traits were slightly higher than their corresponding phenotypic correlations which would be beneficial in the selection of traits because they exclude the environmental influences. It also revealed significant and positive correlation values for seed yield with the number of pods per plant, seeds per plant, shoot fresh weight, days to flowering, and days to maturity as supported by several others (Yadav J. K. et al., 2003; Roy et al., 2019; Rao, 1952 and Grafius, 1964). The observed positive and significant correlations among these traits suggest that beyond seed yield, other characteristics can also be valuable criteria for selecting genotypes for high yield.

### Path coefficient analysis

**Figure 3:**
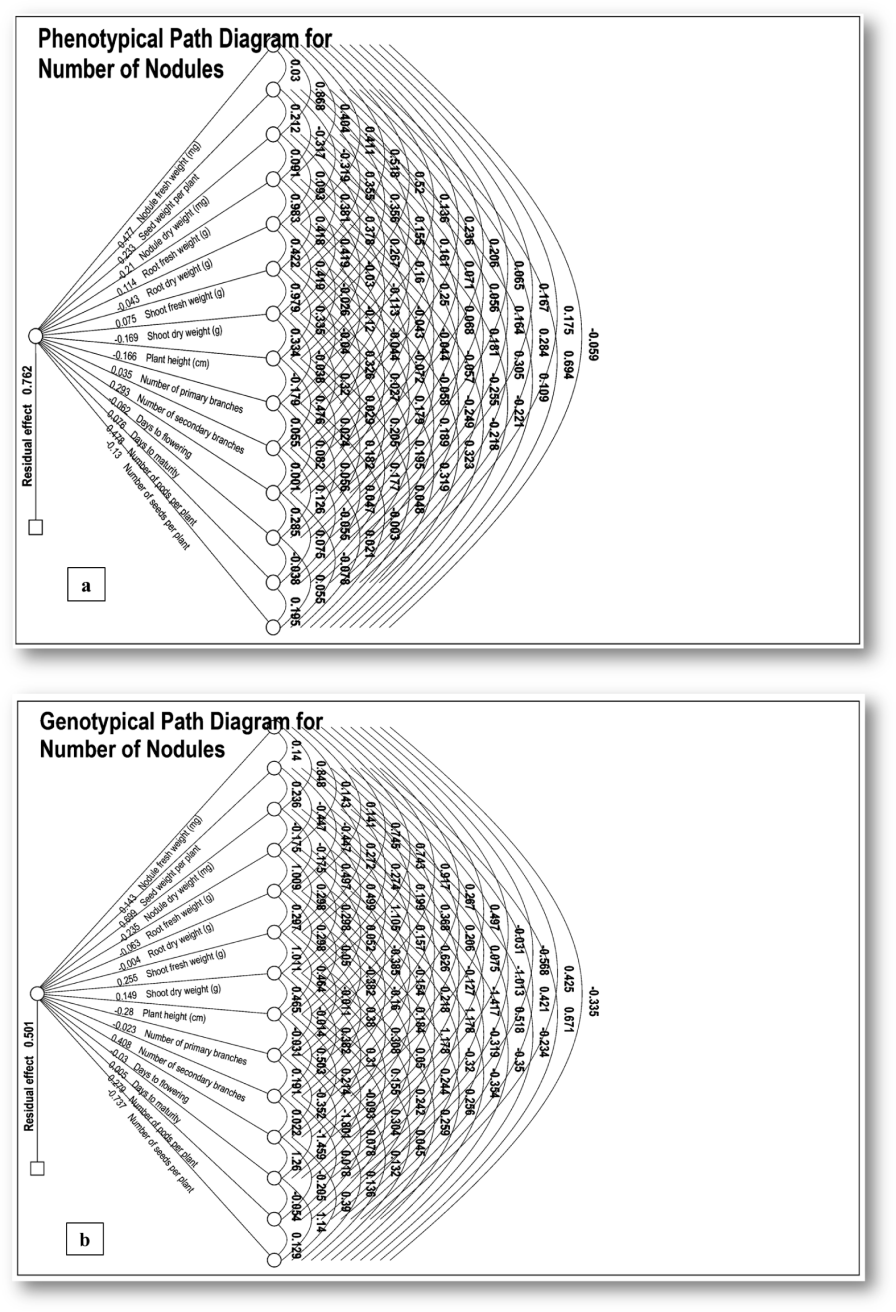
**(a, b)** Path diagrams illustrating **(a)** phenotypic and **(b)** genotypic relationships for the number of nodules and their constituent traits. The path coefficients were analyzed by taking a number of nodules per plant as a dependent character and remaining fourteen characters *viz*. nodule fresh weight (mg), seed weight/plant (g), nodule dry weight (mg), root fresh weight (g), root dry weight (g), shoot fresh weight (g), shoot dry weight (g), days to flowering, days to maturity, number of primary branches, number of secondary branches, number of pods/plants, plant height (cm) and number of seeds/plant as independent variables.

Simple correlation coefficients indicate an association between any two characters, but it does not give a complete picture of a complex relationship. Therefore, it is essential to have path coefficient analysis to get a clear picture of the association among the characters, as it splits the correlation coefficient into the measure of direct and indirect effects of a set of independent variables on the dependent variable through other component traits. The path analysis, a technique developed by Wright (Wright, 1921), was employed to disentangle the direct and indirect influences of the independent characters on the number of nodules per plant. The direct and indirect relationships amongst various characters along with their phenotypic and genotypic path coefficients for the number of nodules per plant are presented (**Supplementary Table S6a, b**; **Figures 3a, b**).

The critical estimation of phenotypic path coefficient analysis for direct effects indicates that the number of pods/plant (0.478) had the highest direct and positive effect on the number of nodules followed by nodule fresh weight (0.477), number of secondary branches (0.293), seed weight/plant (0.233), root fresh weight (0.114), days to maturity (0.076), shoot fresh weight (0.075), number of primary branches (0.035). Similarly, on the other hand, root dry weight (-0.044) had the highest negative straight effects on number of nodules, followed by days to flowering (-0.062), number of seed/plant (-0.130), plant height (-0.166), shoot dry weight (-0.169) and nodule dry weight (-0.210).

At the genotypic level, seed weight/plant (0.699) had the highest direct and positive effect on the number of nodules followed by a number of secondary branches (0.408), number of pod/plant (0.279), shoot fresh weight (0.256), shoot dry weight (0.149), nodule fresh weight (0.143) and days to maturity (0.005). On the other hand, root dry weight (-0.004) had the highest negative straight effects on the number of nodules followed by the number of primary branches (-0.023), days to flowering (-0.030), root fresh weight (-0.063), nodule dry weight (-0.235), plant height (-0.280) and number of seeds/plant (-0.737).

In contrast, indirect effects of diverse plant morphological traits on the quantifiable parameter of nodule count like nodule fresh weight exhibited a notable positive phenotypic influence on nodulation, particularly through nodule dry weight (0.414), shoot dry weight (0.248), shoot fresh weight (0.247) and root dry weight (0.196) among other contributing factors at the phenotypic level. The positive genotypic effect of nodule fresh weight (0.143) on nodules was discerned through intricate pathways, prominently involving plant height (0.131) and nodule dry weight (0.121), alongside other variables. Seed weight per plant (0.233) demonstrated a predominantly positive phenotypic influence on nodules, albeit with a noteworthy negative impact mediated through root parameters. Conversely, nodule dry weight manifested predominantly negative phenotypic effects on nodules, particularly through shoot and root characteristics. Root fresh weight exhibited discernible positive effects, while the impact of root dry weight was characterized by a minute interaction of positive and negative effects. Both shoot fresh weight and shoot dry weight were identified as significant positive contributors to nodulation. Additionally, plant height, although showing limited direct positive effects, predominantly influenced nodulation through the mediation of primary branches. The number of pods per plant (0.449) is identified as a pivotal factor influencing nodulation, exhibiting a notable positive phenotypic effect through diverse interconnected pathways. In contrast, the impact of the number of seeds per plant on nodulation appeared to be more limited in scope. These findings enhance the nuanced comprehension of intricate interactions dictating the formation of nodules in the context of plant development.

These path analysis findings aligned with prior studies offer a nuanced understanding of the intricate interactions among independent traits and the number of nodules per plant. The exploration of direct and indirect contributions at both phenotypic and genotypic levels enhances our comprehension of the underlying mechanisms, contributing valuable insights to the broader knowledge in the field as reported earlier, affirming that the selected set of characters is most suitable for determining the higher yield. Consequently, utilizing these nodule traits as selection criteria for genotype choice holds promise for enhancing the seed yield potential of chickpeas.

### Genetic divergence

The evaluation of genetic divergence through Mahalanobis D2 statistics for 20 chickpea genotypes across fifteen characters yielded meaningful insights. Following (Rao, 1952) Tocher’s method was employed for cluster formation, resulting in the categorization of the 20 genotypes into five clusters. Cluster I emerged as the largest one, encompassing 16 genotypes, while Clusters II, III, IV, and V, each comprised one genotype (**Table 3**).

**Table 3:** Cluster groups for chickpea genotypes under study.

Cluster group	No. of genotypes	List of genotypes
1 Cluster	16	ICC1896, ICC2083, ICC 1172, ICC3093, ICC3631, ICC7167, ICC1852, ICC7305, ICC6995, ICC13185, ICC 1083, ICC6579, ICC1891, ICC9002, ICC4638 & ICC3696
2 Cluster	1	ICC6661
3 Cluster	1	BG 3022 (Check)
4 Cluster	1	ICC9085
5 Cluster	1	BG 372 (Check)

Intra and inter-cluster distances were computed based on the mean D^2^ values of cluster constituents. Notably, Cluster I exhibited the most substantial intra-cluster distance, and the analysis of inter-cluster distances revealed notable variations, with the maximum inter-cluster distance observed between Clusters I and V. By determining cluster means for each trait, significant variability across the genotypes was unveiled. For instance, Cluster IV showcased the maximum number of nodules, Cluster V exhibited the highest nodule fresh weight, and Cluster V had the greatest seed weight per plant. These findings offer valuable insights into the genetic divergence among chickpea genotypes and their clusters based on various traits. Such information is pivotal for understanding chickpea diversity, thereby contributing to the formulation of informed breeding and selection programs.

Consequently, the initiation of hybridization programs incorporating genetically diverse parents from distinct clusters presents an opportunity to amalgamate gene constellations with diverse attributes. This approach may yield promising hybrid derivatives, potentially attributed due to the complementary interaction of divergent genes in the selected parents (Elias, 2009).

### Percent contribution of various characters for divergence

**Figure 4:**
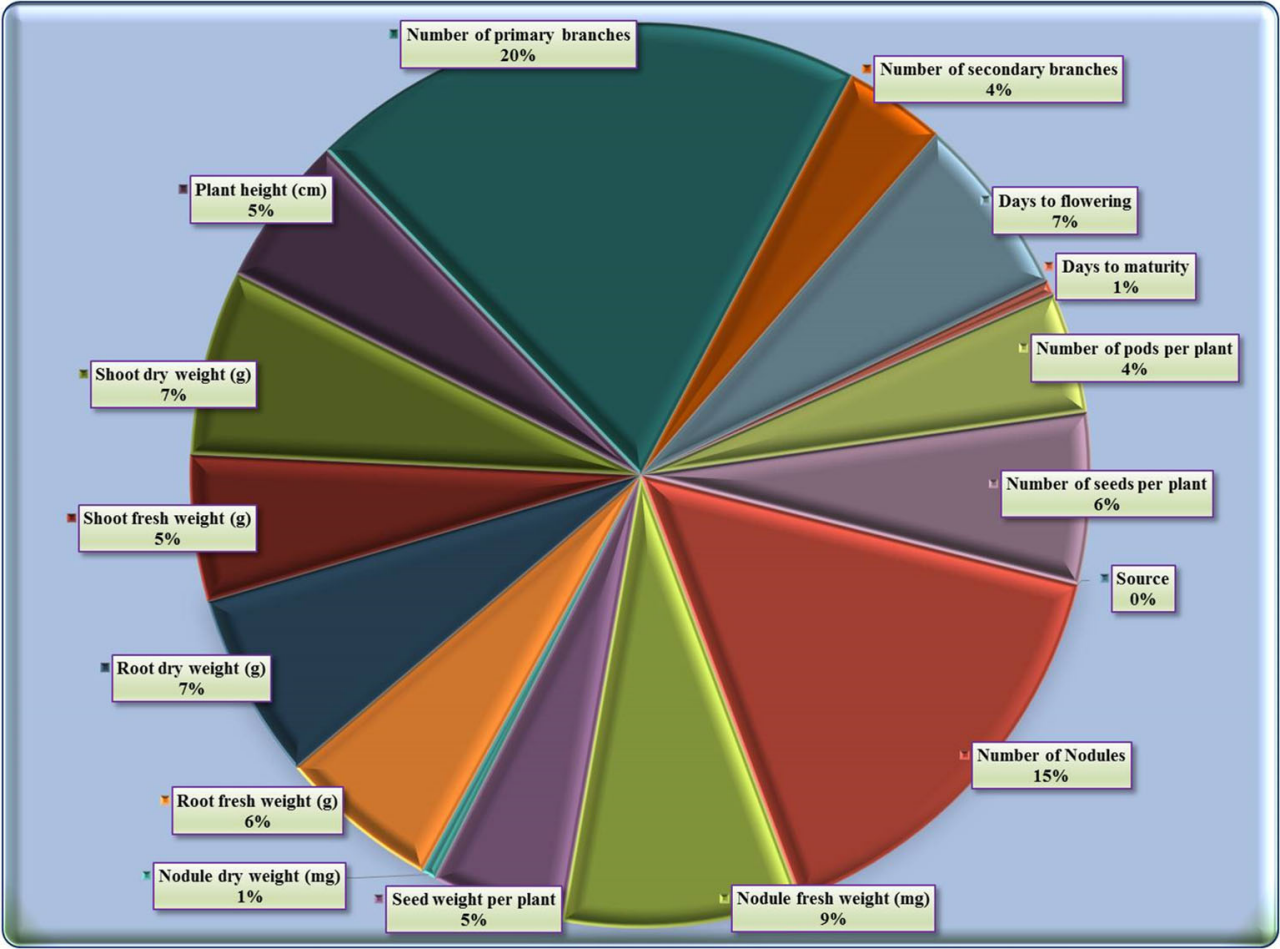
Illustrative diagram depicting the contributions of different traits towards divergence.

The contribution of fifteen studied characters towards genetic divergence was quantified showing that the number of primary branches exhibited the highest contribution (20%) to genetic divergence, followed by the number of nodules (15.38%), nodules fresh weight (8.52%), root fresh weight (5.79%), shoot dry weight (6.62%), days to flowering (6.52%), number of seed/plant (6.1%), root dry weight (6.65%), plant height (5.41%), shoot fresh weight (5.23%), while seed weight/plant (4.74%), number of pods/plant (4.33%), number of secondary branches (3.65%), nodules dry weight (0.53%), and the least one by days to maturity (0.53%) (**Supplementary Table S7**; **Figure 4**).

The ranking analysis revealed that number of primary branches ranked first (38 times) followed by number of nodules (29 times), nodules fresh weight (16 times), root fresh weight (11 times), shoot dry weight (12 times), days to flowering (12 times), number of seed/plant (12 times), root dry weight (13 times), plant height (10 times), and shoot fresh weight (10 times). Conversely, seed weight/plant (9 times), number of pods/plant (8 times), number of secondary branches (7 times), nodules dry weight (1 time), and days to maturity (1 time).

**Figure 5:**
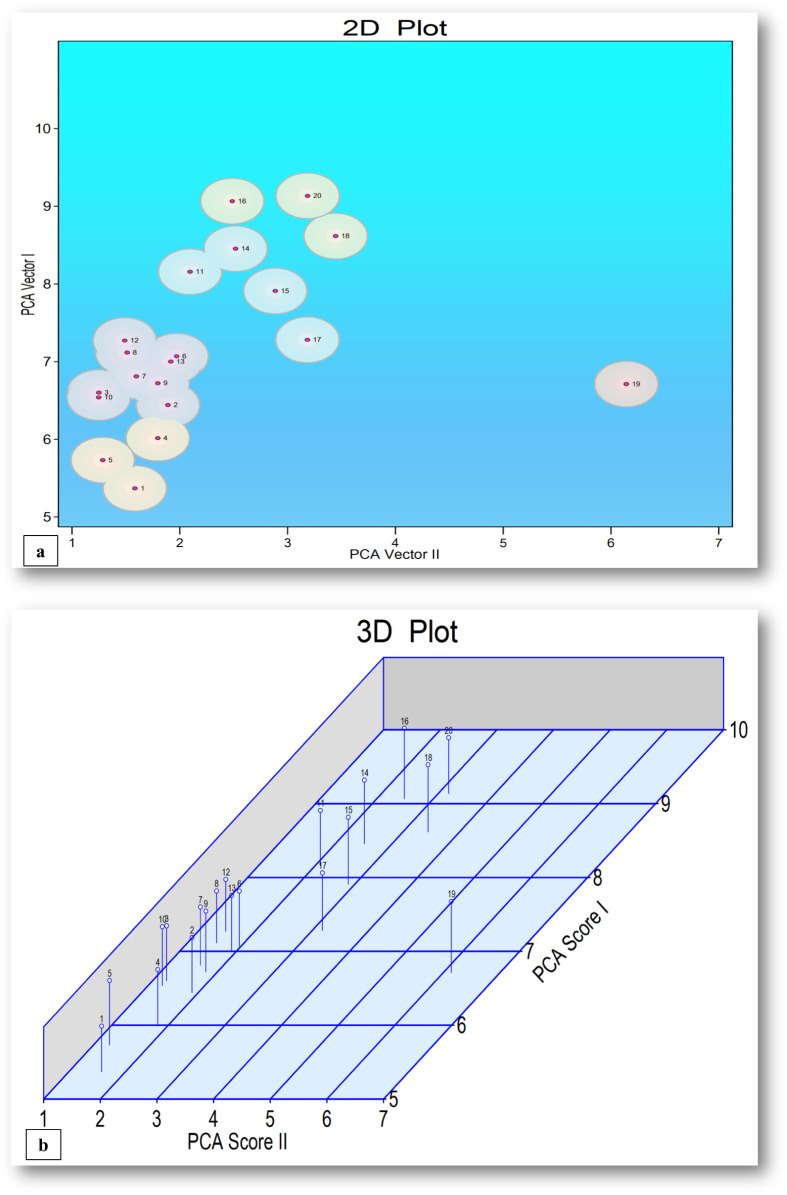
**(a, b)** 2D and 3D plots for chickpea genotypes and treatments utilizing PCA-I and PCA-II scores.

The principal component analysis was conducted to assess morpho-physiological and yield trait variations, with the first principal component explaining the highest percentage of total variation (33.81%) providing an insight into the relative importance of different traits contributing to genetic divergence among chickpea genotypes, which have implications for breeding and selection programs (**Supplementary Table S8**; **Figures 5a, b**).

### Fluorescence imaging

**Figure 6:**
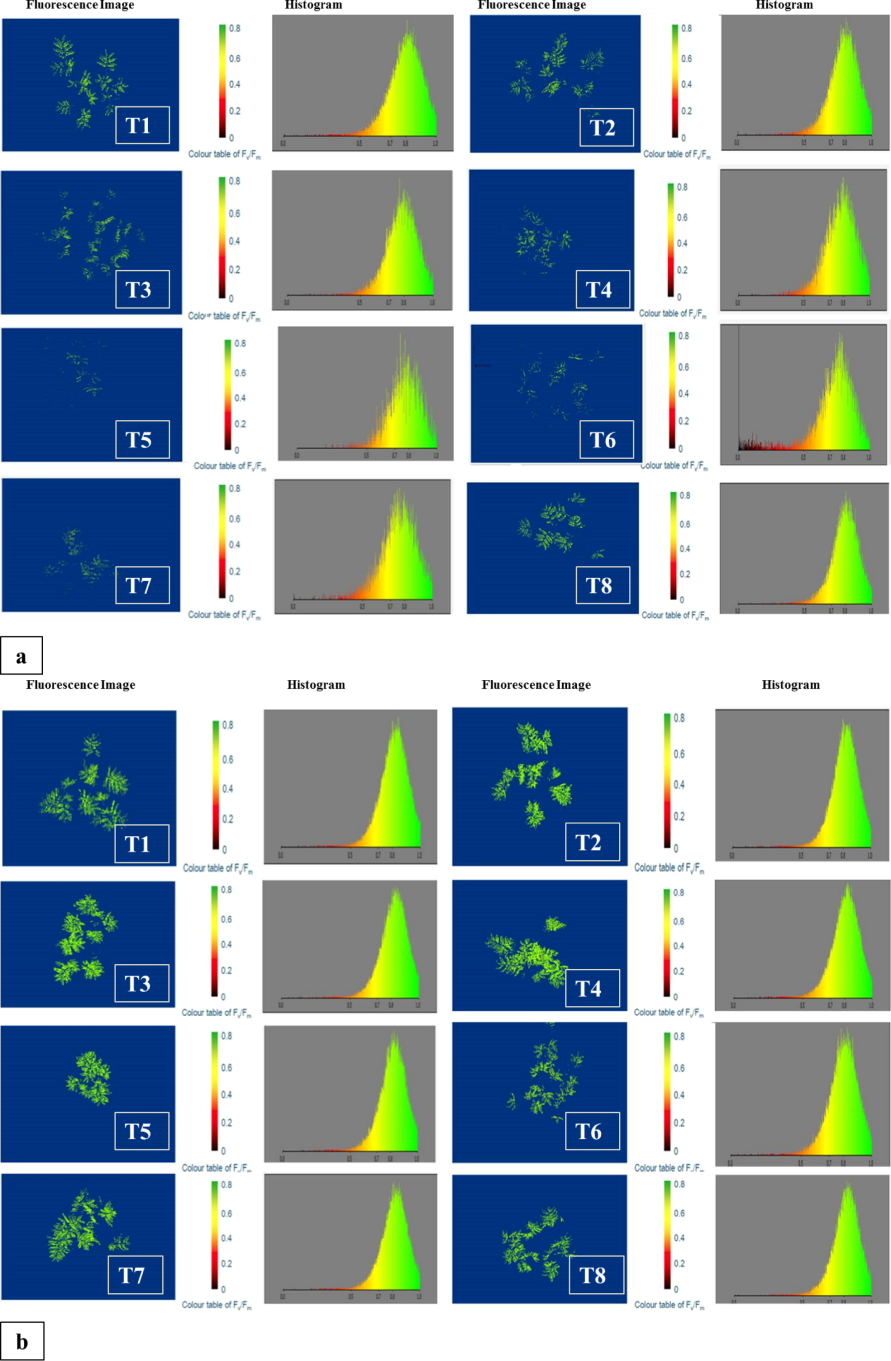
**(a)** Fluorescence image and histogram for the genotype ICC-1083 (low number of Nodules) showing the chlorophyll content for different treatments. The colored bar histogram images from 0.8 to and above show high chlorophyll content and below 0.8 shows low chlorophyll content. **(b)** Fluorescence image and histogram for the genotype ICC-9085 (Higher number of Nodules) shows the chlorophyll content for different treatments. The colored bar histogram images from 0.8 to and above show high chlorophyll content and below 0.8 shows low chlorophyll content.

Fluorescence imaging and histogram analysis were performed on ICC-1083 and ICC-9085, the genotypes characterized as low and high nodule counts were grown in 8 environments (treatments) Control (Treatment 1), RDF (Treatment 2), *Mesorhizobium* (Mr) Reference strain (Treatment 3), VAM (Treatment 4), VAM+RDF (Treatment 5), Mr reference strain IARI + RDF (Treatment 6), Mr reference strain IARI + VAM (Treatment 7), and Mr reference strain IARI + VAM+RDF (Treatment 8) as illustrated in (**Figures 6a, b**).

The fluorescence images consistently depicted comparatively diminished chlorophyll levels and photosystem-II activity across all the eight treatments for the genotype ICC-1083 in comparison to ICC-9085. The attached image figures, bar graphs cum related data files 1 and 2 captured and analyzed by CropReporter™ (PhenoVation, Netherlands) during the fluorescence imaging clearly show the superiority of treatment 7 over the other treatments with the highest Y-Top values for the studied genotypes ICC-9085 and ICC-9083. This observation was also corroborated by histogram analysis, where values below 0.8 indicated low chlorophyll content and low photosystem -II (PS-2) activity across treatments. Notably, treatment 7 (RZ+VAM) exhibited the highest chlorophyll content and higher photosystem -_II activity among all treatments. Comparative analysis revealed a descending gradient of chlorophyll and PS-2 response, with treatment 7 showing the highest chlorophyll content and PS-2 activity, followed by treatments 8, 6, 4, 5, 3, 2, and 1. Treatment 1, serving as the control without fertilizer or inoculant, demonstrated the most gradual increase in chlorophyll content. Consequently, ICC-9085 emerged as a prominent genotype, with treatment 7 (Mr reference strain IARI + VAM) identified as most promising for further breeding programs. These findings underscore the potential of treatment 7 in enhancing chlorophyll production, PS-2 activity and support for the selection of ICC-9085 for future breeding endeavors aimed at improving crop traits.

Agronomic and physiological traits such as number of nodules, nodule dry weight, root biomass, chlorophyll content, and grain yield have been systematically analyzed in relation to the eight treatment combinations (T1–T8). Among these, Treatment 7 (T7: Mr + VAM) consistently demonstrated the best overall performance, particularly in nodule number, chlorophyll content, and grain yield. The synergistic interaction between *Mesorhizobium* and VAM is interpreted to enhance root colonization, nutrient assimilation (notably nitrogen and phosphorus), and symbiotic efficiency, thereby promoting higher nodulation and plant biomass. The significantly increased chlorophyll content observed under T7 reflects improved nitrogen status and photosynthetic capacity, contributing directly to superior yield. Trait-specific highlights for other treatments are as follows:T1 (Control): As expected, the lowest values across all traits, indicating the baseline response without microbial or nutrient inputs., T2 (RDF only): Showed moderate improvement in grain yield and biomass, attributable to direct nutrient availability, though lacking symbiotic enhancement, T3 (Mr only): Led to a notable increase in nodule number and nodule dry weight, indicating effective *Rhizobium*-mediated nodulation, albeit with limited impact on chlorophyll content and yield, T4 (VAM only): Resulted in a higher root biomass and improved phosphorus uptake, supporting the known role of VAM in root development and nutrient acquisition, T5 (RDF + Mr): Improved both nodule dry weight and biomass accumulation, suggesting additive effects of chemical and microbial inputs., T6 (RDF + VAM): Showed enhanced root biomass and chlorophyll content, highlighting the role of VAM in nutrient uptake and RDF in chlorophyll biosynthesis, T8 (RDF + Mr + VAM): Although showing comparable performance to T7 in certain traits, it did not surpass T7, possibly due to complex nutrient-microbe interactions diluting the synergistic effect observed in the Mr + VAM treatment alone.

### Nitrogen estimation

**Figure 7:**
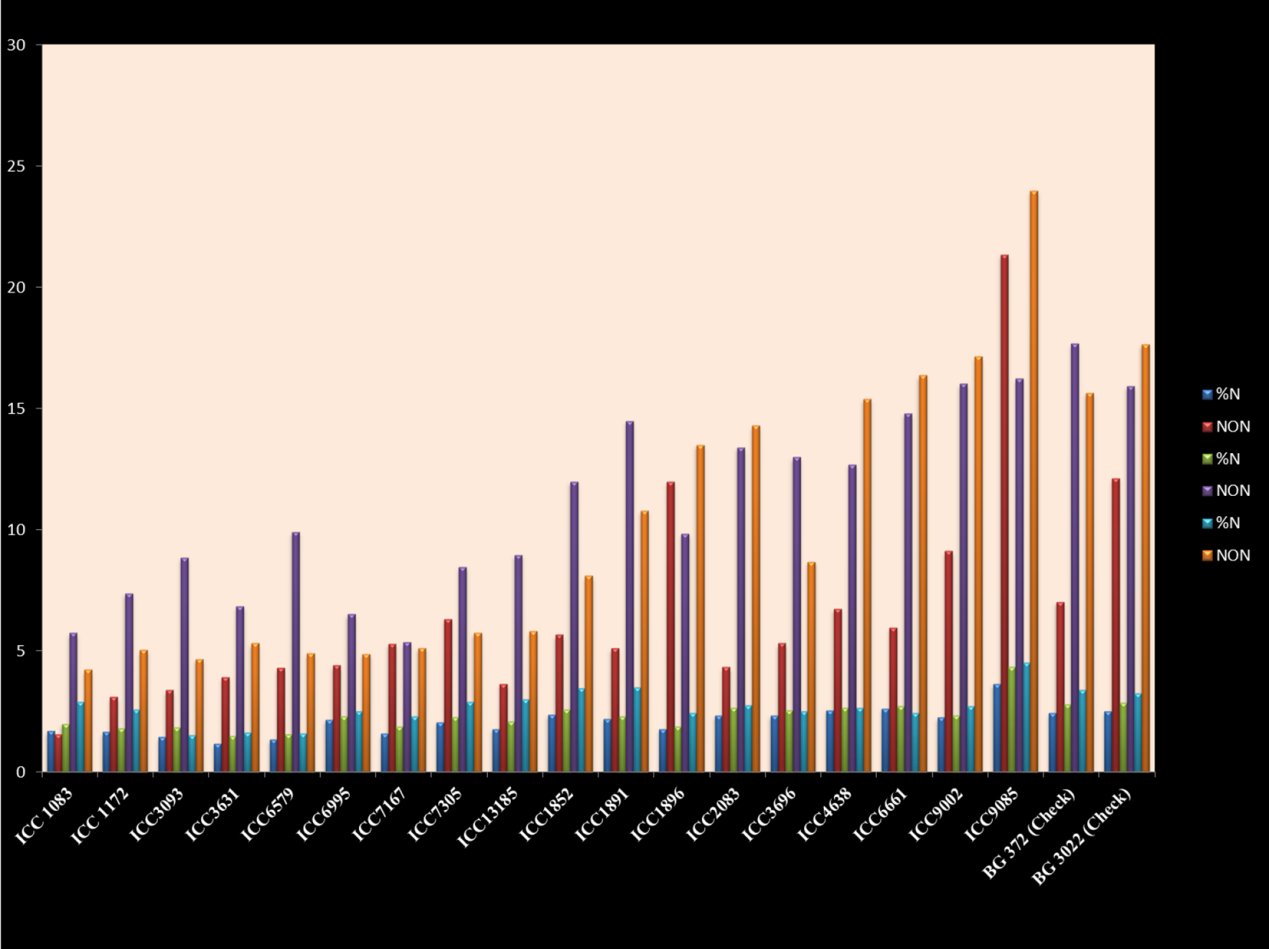
Number of nodules and nitrogen contents in chickpea genotypes in control, VAM, and *Rhizobium* treatments.

Nodules facilitated by the symbiotic interaction with nitrogen-fixing bacteria, notably *Rhizobium* species manifest as specialized structures on the roots of legume plants including chickpeas. The discernible presence of nodules bears a direct correlation with the exhibited nitrogen-fixing capability. This relationship is characterized by an augmented nodule count, signifying increased nitrogen content within the chickpea genotypes. This, in turn, exerts a favorable influence on the holistic growth and developmental trajectory of the plant. The pivotal role of the bacterial partners lies in their conversion of atmospheric nitrogen into a readily assimilable form by the plant, thus enhancing the overall nitrogen availability to the host. Through meticulous scrutiny of the nodule count data, a deduction can be drawn that each genotype establishes a mutualistic rapport with the nitrogen-fixing bacteria. However, the extent of this association is discernibly variable due to the observed disparities in nodule quantities. The quantification of nitrogen’s proportion, as represented by the percentage of nitrogen (%) in chickpeas, holds a critical significance in their nutritional context, profoundly influencing their growth and developmental patterns. The N (%) metric serves as an indicator of the nitrogen quantum present within the plants, implying a direct correlation with their nitrogen uptake and utilization efficiency. Employing Kjeldahl’s method for nitrogen estimation, as delineated in the AOAC (AOAC, 1990) guidelines, facilitated the determination of the percentage of total nitrogen. The ensuing variability in N (%) values spans a range from 1.69% to 4.504%, encapsulating the diverse nitrogen composition among the genotypes. Notably, the genotype ICC-9085 attains zenith with an N (%) value of 4.504%, whereas the ICC-1083 genotype occupies the bottom with an N (%) value of 1.693% (**Supplementary Table S9**; **Figure 7**).

As nodule number, nodule dry weight, root architecture, biomass accumulation, and chlorophyll content to their well-documented roles in enhancing symbiotic efficiency and yield outcomes. For instance, increased nodule number and biomass under treatments which is T7 (Mr + VAM) are discussed in light of improved nitrogen fixation capacity and nutrient uptake, aligning with findings by Kumar et al. (2017) and Ferguson et al. (2019). The development of a robust root system is interpreted as a facilitator of enhanced mycorrhizal colonization and resource acquisition, supported by Gopalakrishnan et al. (2015). Elevated chlorophyll content is presented as an indirect marker of nitrogen sufficiency and photosynthetic performance. The Treatment 7 (Mr + VAM), which combines the *Rhizobium* strain with the VAM inoculum, showed excellent performance for the genotype ICC-9085.

These figures indicate a substantial disparity in nitrogen content between ICC-9085 and ICC-1083, a phenomenon further corroborated by the accompanying nodule counts of 23.98 and 1.5 per plant, respectively.

### Expression profiling

For expression analysis of genes involved in the nodule formation and N synthesis assimilation, a comprehensive literature survey was done and found many genes that were responsible for the aforesaid trait. Based on the survey, we selected the five most prominent genes that were playing important roles in nodule formation and nitrogen synthesis viz; *Ca_03442* (Glutathione S-transferase), *Ca_16084* (Leghemoglobin), *Ca_15466* (Nodulin6) as per Afonso-Grunz et al., 2014 andLOC101499326 (*CaLYK3*) and LOC101515074 *(CaNFP)* as per Singh and Verma as presented in **Table 4**.

**Table 4:** The genes and their details for root nodules used in the present chickpea gene expression analysis.

Gene	Primer sequence (5’-3’)		Reference
*Ca_15466* (Nodulin 6)	Forward	ACTGATGCCTATGCATTTCCTGAAC	Afonso-Grunz et al (2014)
	Reverse	ACTTCCACAGCCTCCGGAAC	
*Ca_16084* (Leghemoglobin)	Forward	GAGATGCTACATTGGGTGCTGTTC	
	Reverse	GCCAATCCATCATAGGCGAGTTC	
*Ca_03442* (Glutathione S-Transferase)	Forward	GGAAGAGAATGAAGCCAAGTTGAACAC	
	Reverse	TAGACCAAGCTGGTCTTGCAGTG	
LOC101515074 *(CaNFP)*	Forward	GATGCTTCTGAGGAGCT	Singh and Verma (2002)
	Reverse	GTCCGAAGACACACCCATTAG	
LOC101499326 *(CaLYK3)*	Forward	AGGGTCTTGTAGCCTTGTTTG	
	Reverse	AGTTGAGCCATCTTGAGAACAG	

**Figure 8:**
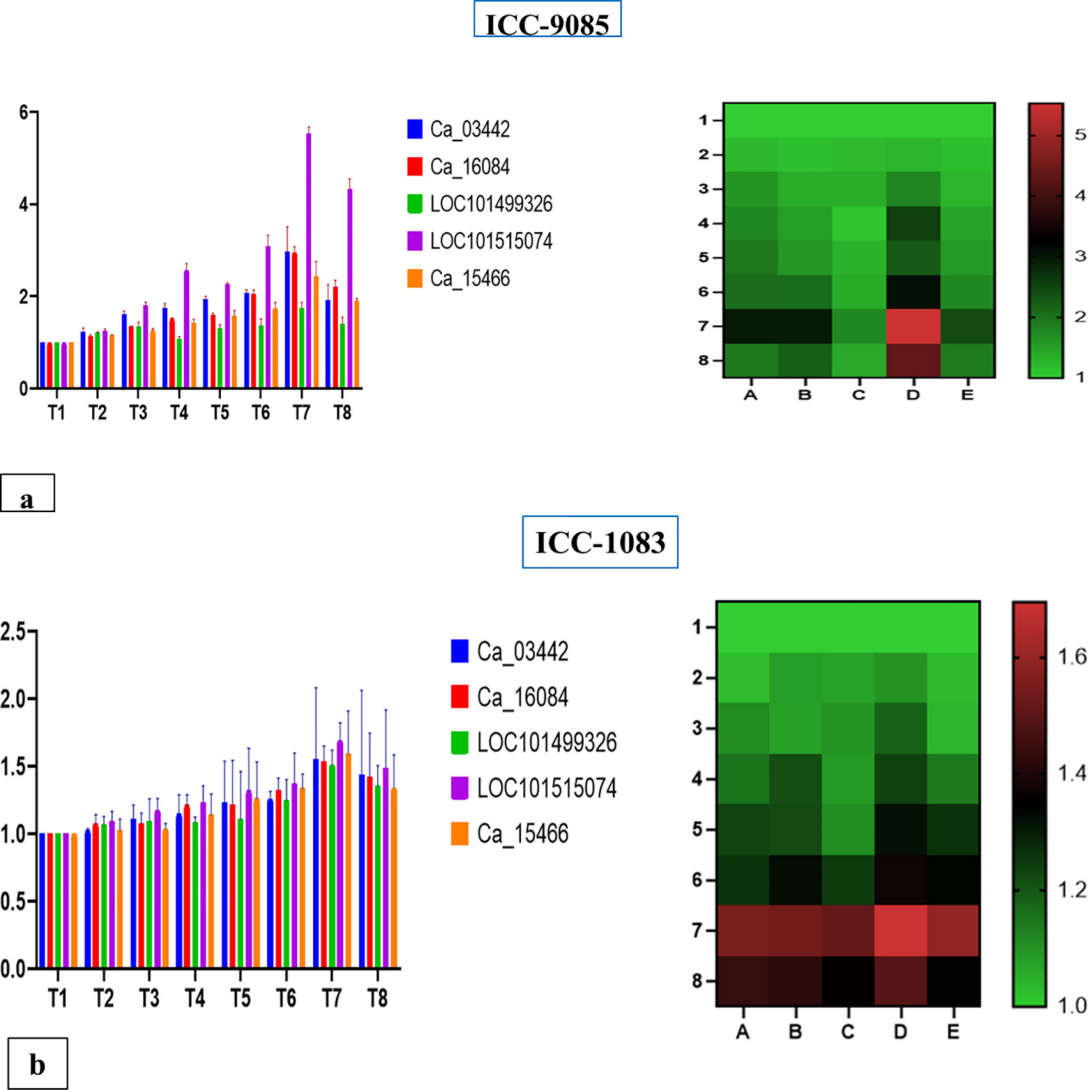
**(a)** Comparative gene expression analysis for chickpea root nodulation with highest fold change in *CaNFP* followed by glutathione s transferase, leghemoglobin, Nodulin6 and *CaLYK3* primers in control and treatments. **(b)** Comparative gene expression analysis for chickpea root nodulation with highest expression fold change in *CaNFP*followed by Glutathione transferase, leghemoglobin, Nodulin6 and *CaLYK3* primers in control and treatments.

From the expression analysis, we found that all the selected genes were differentially expressed in both genotypes and have a varying degree of response in various treatments. In all the treatments ICC-9085 had a high fold change in the expression of genes as compared to the ICC-1083 (**Figures 8a, b**).

Treatment 7(RZ+VAM) had a high expression of genes in both the genotypes for all five genes. Among treatments, T7>T8>T6>T4>T5>T3>T2 and T1 best gradual response for genes was observed. Where, T1=Control (No fertilizer, No inoculant), T2=RDF (DAP 100 Kg/ha equivalent to 20KgN and 40 Kg P), T3= *Mesorhizobium* (Mr) Ref strain IARI, T4=VAM, T5=VAM+RDF, T6=Mr (ref strain IARI)+RDF, T7=Mr (Ref strain IARI) +VAM, T8=Mr (Ref strain IARI) +VAM+RDF.

Out of five selected genes, LOC101515074 had a significant impact on different treatments. From the expression analysis, we inferred that the treatment of *Rhizobium*+Mycorrhiza had a direct effect on the nodule-related genes that led to the production of a high number of nodules, which increased the biomass and yield of chickpea.

Gene expression profiles and nitrogen content with key phenotypic traits such as nodule number, nodule dry weight, root biomass, shoot biomass, and overall plant vigor. For instance, the superior-performing genotype ICC-9085 exhibited significantly higher transcript levels of nodulation and nitrogen assimilation-related genes, including *CaNFP*, *Leghemoglobin*, and *CaLYK3*, under the T7 treatment (Meso*Rhizobium* + VAM). These elevated gene expression levels were positively associated with greater nitrogen accumulation (as measured by %N through Kjeldahl’s method) and improved nodulation traits specifically higher nodule number, greater nodule biomass, and increased root/shoot mass demonstrating a functional alignment between molecular and physiological responses. Similarly, low-nodulating genotypes displayed reduced expression of the same genes and correspondingly lower nitrogen uptake and biomass, reinforcing the reliability of the gene-trait relationships. This enhanced integration strengthens the biological interpretation of the experimental data and underscores the effectiveness of combined microbial treatments (Mr + VAM) in promoting symbiotic efficiency and nitrogen fixation, ultimately contributing to improved plant performance and yield potential.

VAM enhances phosphorus solubilization and uptake, which is crucial for optimal root development and energy metabolism—both of which are preconditions for effective *Rhizobium* colonization. In turn, *Rhizobium* initiates nodulation through the activation of *nod* genes and the synthesis of Nod factors, which are recognized by chickpea root receptors to trigger nodule organogenesis. This complementary interaction is biologically synergistic: VAM improves root architecture and nutrient availability (especially phosphorus), while *Rhizobium* contributes biologically fixed nitrogen together resulting in higher nodule number, improved plant biomass, and enhanced expression of key nodulation genes, including *CaNFP*, *Leghemoglobin*, and *Nodulin6*. The integrated treatment T7 (Meso*Rhizobium* + VAM) consistently exhibited superior performance across multiple traits and genotypes, especially in the high-nodulating line ICC-9085. These results underscore the functional synergy between the two microbial partners in promoting plant growth and symbiotic efficiency. Furthermore, this tripartite approach offers a viable strategy for reducing dependency on synthetic fertilizers and promoting sustainable chickpea productivity, especially under nutrient-limited or stress-prone climatic conditions.

High-nodulating genotype ICC-9085 consistently exhibited significantly elevated expression levels of key nodulation-related genes, including *CaNFP*, *Leghemoglobin*, *GST*, and *CaLYK3*, particularly under the T7 treatment (Meso*Rhizobium* + VAM). These upregulated gene expression patterns closely aligned with superior phenotypic performance, such as higher nodule number, greater nodule dry weight, enhanced nitrogen content (as quantified by Kjeldahl’s method), and increased shoot and root biomass. This strong molecular-to-phenotypic correlation underscores the functional relevance of these genes in promoting efficient symbiotic nitrogen fixation. In contrast, the low-nodulating genotype ICC-1083 showed markedly reduced expression of the same gene set, which was reflected in its poor nodulation efficiency, lower nitrogen accumulation, and diminished plant growth. This comparative analysis reinforces the role of genotype × treatment interaction in driving symbiotic efficiency and plant performance under combined microbial inoculation.

Various treatments, as well as their combinations, exerted notable influences on the expression profiles of the genes examined in this study. Specifically, the combination denoted as T7, comprising RZ treatment in conjunction with VAM, emerged as the most effective treatment regimen. Within the T7 treatment regimen, the expression levels of certain genes displayed remarkable alterations. Notably, the gene encoding Calcium and Calmodulin-dependent protein kinase (*CaNFP*) exhibited the highest expression levels among the genes under investigation. This finding aligns with previous research that has highlighted the significance of *CaNFP* in various plant physiological processes, particularly its role in calcium signaling and stress responses. Additionally, within the T7 treatment, the expression of Glutathione S-transferase (GST) showed a substantial increase. GST is known for its pivotal role in detoxification processes and its involvement in the plant’s defense mechanisms against environmental stress. Furthermore, the genes associated with Leghemoglobin, Nodulin6, and Calcium-dependent protein kinase 3 (*CaLYK3*) also exhibited elevated expression levels with theT7 treatment. Leghemoglobin is essential for nitrogen fixation in root nodules of leguminous plants, while Nodulin6 is closely linked to the nodulation process in legumes. *CaLYK3* is a key component of calcium signaling pathways and has been implicated in various plant responses to environmental cues. ICC-9085 (a superior nodulating genotype) and ICC-1083 (a poor nodulating genotype). Their contrasting performance has been systematically emphasized across sections focused on phenotypic, genotypic, and biochemical evaluations. Clear differences in nodulation efficiency, gene expression levels (e.g., *CaNFP*, *Leghemoglobin*, *Nodulin6*), and responsiveness to microbial treatments such as *Rhizobium* and VAM have been outlined. This genotype-level contrast not only strengthens the interpretation of experimental outcomes but also offers valuable insights into genotype–treatment interactions. Both ICC-9085 and ICC-1083 have been consistently incorporated throughout figures, tables, and data narratives, reinforcing the study’s central objective. Additionally, ICC-9085 has been identified as a promising candidate for further genomic analysis and targeted genome editing to enhance nodulation traits in chickpea.

Each treatment (T1–T8) affected both the high-nodulating genotype (ICC-9085) and the low-nodulating genotype (ICC-1083). Further, it is explicitly demonstrated that ICC-9085 responded very well, especially under Treatment T7 (Meso*Rhizobium* + VAM), which led to a high number of nodules, increased biomass, and higher expression of key nodulation-related genes (*CaNFP*, *Leghemoglobin*, *Nodulin6*). This confirms its status as a strong nodulator. In contrast, ICC-1083 showed poor response under the same treatment, with low nodulation and gene expression levels, supporting its classification as a low-performing genotype. Comparing the two genotypes under all treatments, a more complete picture of how genotype × treatment interactions influence nodulation efficiency and plant performance has been provided. This helps highlight the synergistic effect of microbial inoculants and how their impact can vary depending on genotype. Further, to address the reviewer’s concern regarding the separation of treatment effects, the results have now been distinctly presented with respect to low and high nodulating genotypes. For each microbial and nutrient treatment, the differential responses of ICC-9085 and ICC-1083 have been evaluated independently. High nodulating genotypes, particularly ICC-9085, demonstrated significant positive responses in nodule number, dry weight, nitrogen content, and expression of nodulation-related genes, especially under combined treatments (e.g., *Rhizobium* + VAM). In contrast, low nodulating genotypes, including ICC-1083, showed minimal or no significant improvement across these parameters under the same treatment regimes.

Our findings on the direct and indirect effects of key nodulation-related traits. Specifically, we now compare our observations on traits such as nodule number, fresh and dry biomass, chlorophyll content, and shoot/root ratio with previously published studies in legumes, particularly chickpea. For example, Kumar et al. (2017) demonstrated that increased nodule number and biomass are significantly correlated with higher nutrient assimilation and yield traits in chickpea. Similarly, Ferguson et al. (2019) provided insights into the molecular and physiological pathways by which improved nodulation positively influence photosynthetic efficiency and overall plant productivity. These citations have been incorporated to strengthen the interpretation of trait interactions and their contributions to yield enhancement.

Thus, it is summarized that the combined treatment of RZ and VAM (T7) demonstrated the most significant impact on gene expression profiles with notable upregulation observed in CaNFP, GST, Leghemoglobin, Nodulin6, and *CaLYK3* genes. These findings emphasize the intricate interplay of various treatments on gene expression in plants and emphasize the potential utility of the RZ+VAM combination in enhancing specific molecular responses.

CRISPR/Cas9 system, could be leveraged to enhance symbiotic nitrogen fixation in chickpea. Based on our gene expression results, we propose that precise editing of key regulatory genes such as *CaNFP*, which exhibited a strong association with superior nodulation traits in the high-performing genotype ICC-9085 could be applied to improve nodulation efficiency in underperforming genotypes like ICC-1083. Targeted modification of *CaNFP* has the potential to enhance early signaling and nodule organogenesis. Furthermore, multiplex genome editing strategies could be employed to simultaneously edit other promising candidate genes, including *CaLYK3*, *Leghemoglobin*, and *GST*, thereby improving the overall symbiotic effectiveness. This genome engineering-based approach underscores the translational relevance of our findings and opens new avenues for the development of high-performing, nitrogen-efficient chickpea cultivars adapted to resource-constrained environments.

### Future prospects for harnessing targeted manipulation

The advancement of genome-editing technologies such as zinc finger nucleases (ZFNs), transcription activator-like effector nucleases (TALENs), and the CRISPR/Cas systems presents unprecedented opportunities for chickpea (*Cicer arietinum* L.) improvement. These technologies enable precise genetic modifications, allowing the introduction, deletion, or alteration of specific traits linked to agronomic performance. In particular, the CRISPR/Cas system, owing to its simplicity, high efficiency, and versatility, has revolutionized targeted gene editing and holds immense promise for functional genomics and trait enhancement in chickpea. Future research could be focus on the targeted manipulation of key genes such as CaNFP, associated with nodulation and biological nitrogen fixation, to optimize symbiotic efficiency and improve crop yield and we propose to harness the strategy as advocated in tomato by us (Yadav R. et al., 2023). Moreover, integrating multiplex genome editing approaches may enable simultaneous modifications of multiple genes controlling complex traits such as stress tolerance, nutrient use efficiency, and seed quality. Combined with emerging base-editing and prime-editing tools, genome editing is expected to accelerate chickpea breeding programs, offering sustainable solutions to meet global food security demands (Chen et al., 2019; Zhu et al., 2020).Based on the differential gene expression and phenotypic performance of the studied genotypes, key candidate genes, *CaNFP* (Nod factor perception), *CaLYK3* (LysM receptor kinase), and *Leghemoglobin*, have been identified as critical regulators of symbiotic efficiency in chickpea. We propose that CRISPR/Cas9-mediated editing could be strategically applied to enhance or activate these genes in low-nodulating genotypes such as ICC-1083, thereby emulating the superior nodulation efficiency as observed in high-performing genotypes like ICC-9085. Moreover, the use of multiplex CRISPR approaches could enable the simultaneous editing of multiple nodulation-associated loci, offering a comprehensive strategy to boost nitrogen fixation. These interventions are expected to result in increased nodule formation, improved nitrogen assimilation, reduced reliance on synthetic fertilizers, and overall yield enhancement, particularly under low-input and sustainable agricultural systems. Such targeted genome editing efforts will bridge functional genomics with precision breeding, accelerating chickpea improvement programs.

## Conclusion

The comprehensive evaluation of eight treatment combinations, including Rhizobium (RZ) and vesicular-arbuscular mycorrhiza (VAM), revealed their significant influence on nodulation traits in chickpeas. Heritability estimates correlated positively with yield parameters, and correlation and path analysis emphasized the potential of nodulation-based selection strategies for yield improvement. Among treatments, the combined application of RZ+VAM (T7) was the most effective, notably enhancing nodulation traits and upregulating key nodulation-associated genes such as CaNFP, GST, Leghemoglobin, Nodulin6, and CaLYK3, with CaNFP emerging as a pivotal regulator. Genotype ICC-9085 was identified as a promising donor with a novel, stable, and high-nodulation phenotype. Gene expression profiling through qRT-PCR corroborated the superior performance of the T7 treatment, highlighting its potential for enhancing symbiotic efficiency. The availability of the chickpea genome opens avenues for genome editing strategies, such as CRISPR/Cas targeting the CaNFP gene, to further improve nodulation and plant vigor. The integration of microbial treatments and genome editing can accelerate the development of elite chickpea cultivars, thereby reducing dependence on chemical fertilizers and promoting sustainable agriculture. Overall, the findings underline the critical role of RZ+VAM treatment, the genotype ICC-9085 and CaNFP the key nodulation-associated gene suitable for biofortification through genome editing, will be valuable resources for enhancing nodulation and achieving sustainable higher yield and productivity in chickpea.

## Data Availability

The datasets presented in this study can be found in online repositories. The names of the repository/repositories and accession number(s) can be found in the article/**Supplementary Material**.
