# Supplementary material for: Symbiotic nitrogen fixation for sustainable chickpea yield and prospects for genome editing in changing climatic situations
Source: Front Plant Sci. 2025 Sep 1;16:1621191. doi: 10.3389/fpls.2025.1621191 (PMC12433944; doi:10.3389/fpls.2025.1621191)
Supplement: Supplementary file 1 [file Presentation1.pptx]

## Slide 1
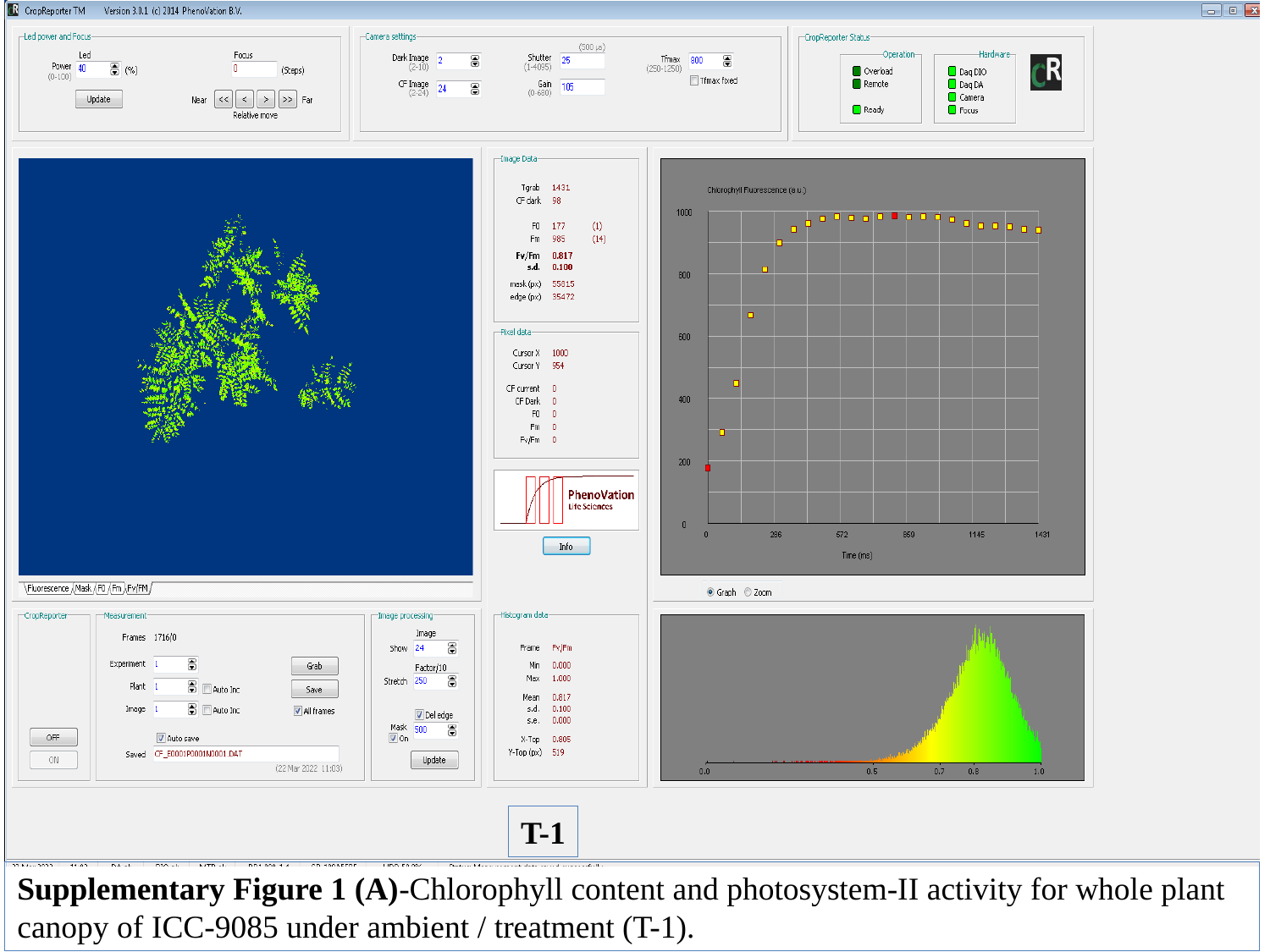

T-1
Supplementary Figure 1 (A)-Chlorophyll content and photosystem-II activity for whole plant canopy of ICC-9085 under ambient / treatment (T-1).

## Slide 2
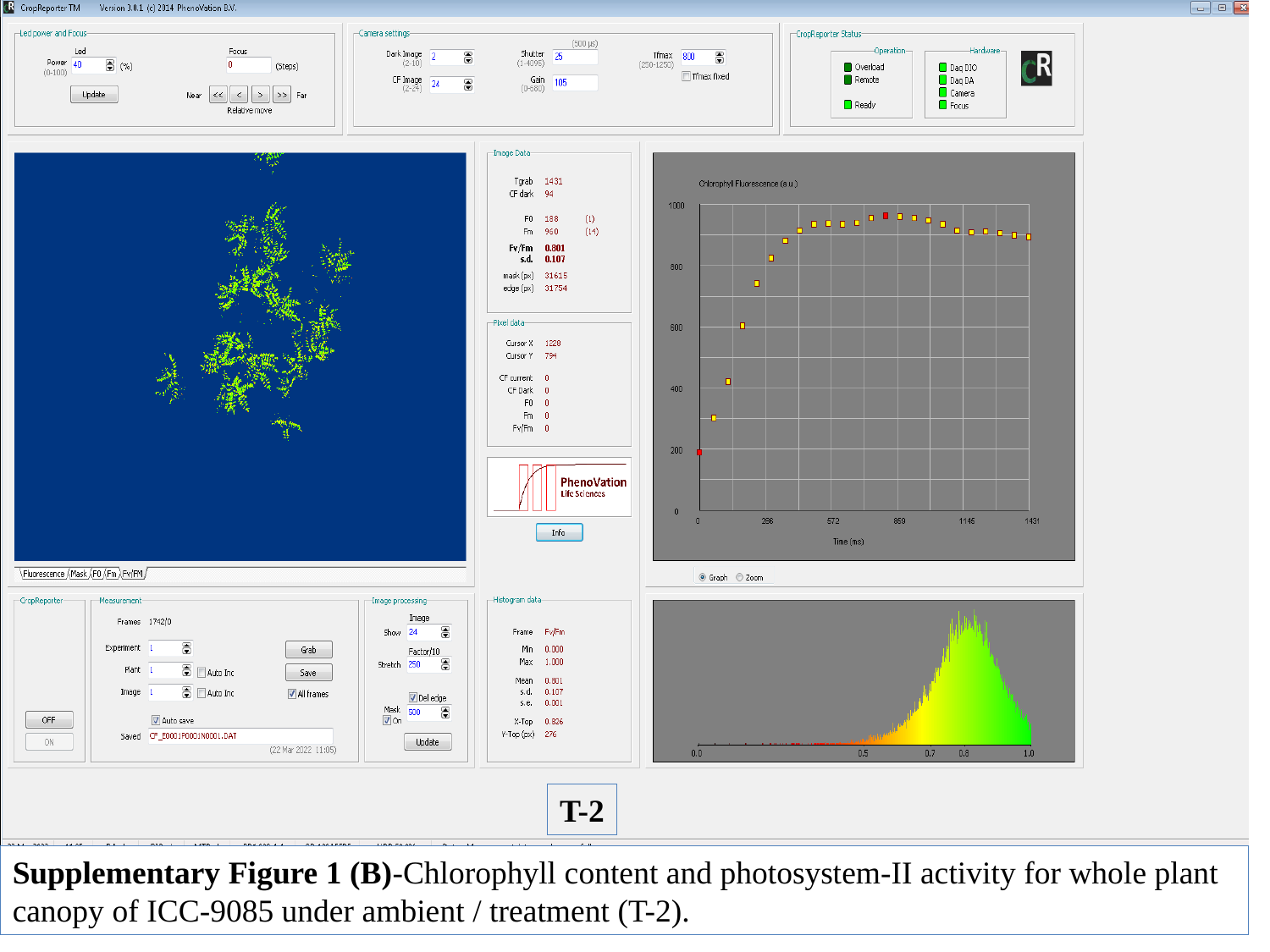

T-2
Supplementary Figure 1 (B)-Chlorophyll content and photosystem-II activity for whole plant canopy of ICC-9085 under ambient / treatment (T-2).

## Slide 3
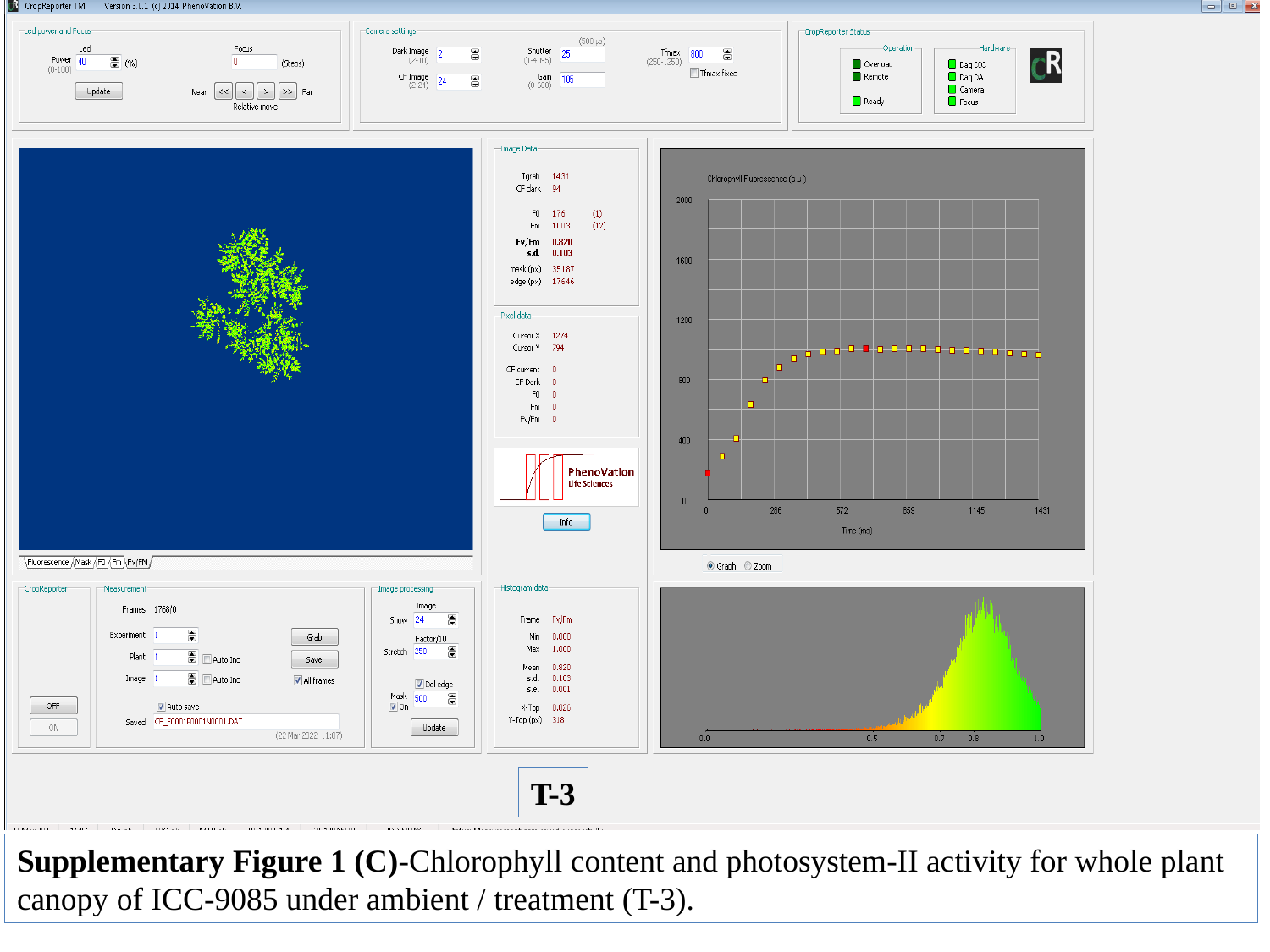

T-3
Supplementary Figure 1 (C)-Chlorophyll content and photosystem-II activity for whole plant canopy of ICC-9085 under ambient / treatment (T-3).

## Slide 4
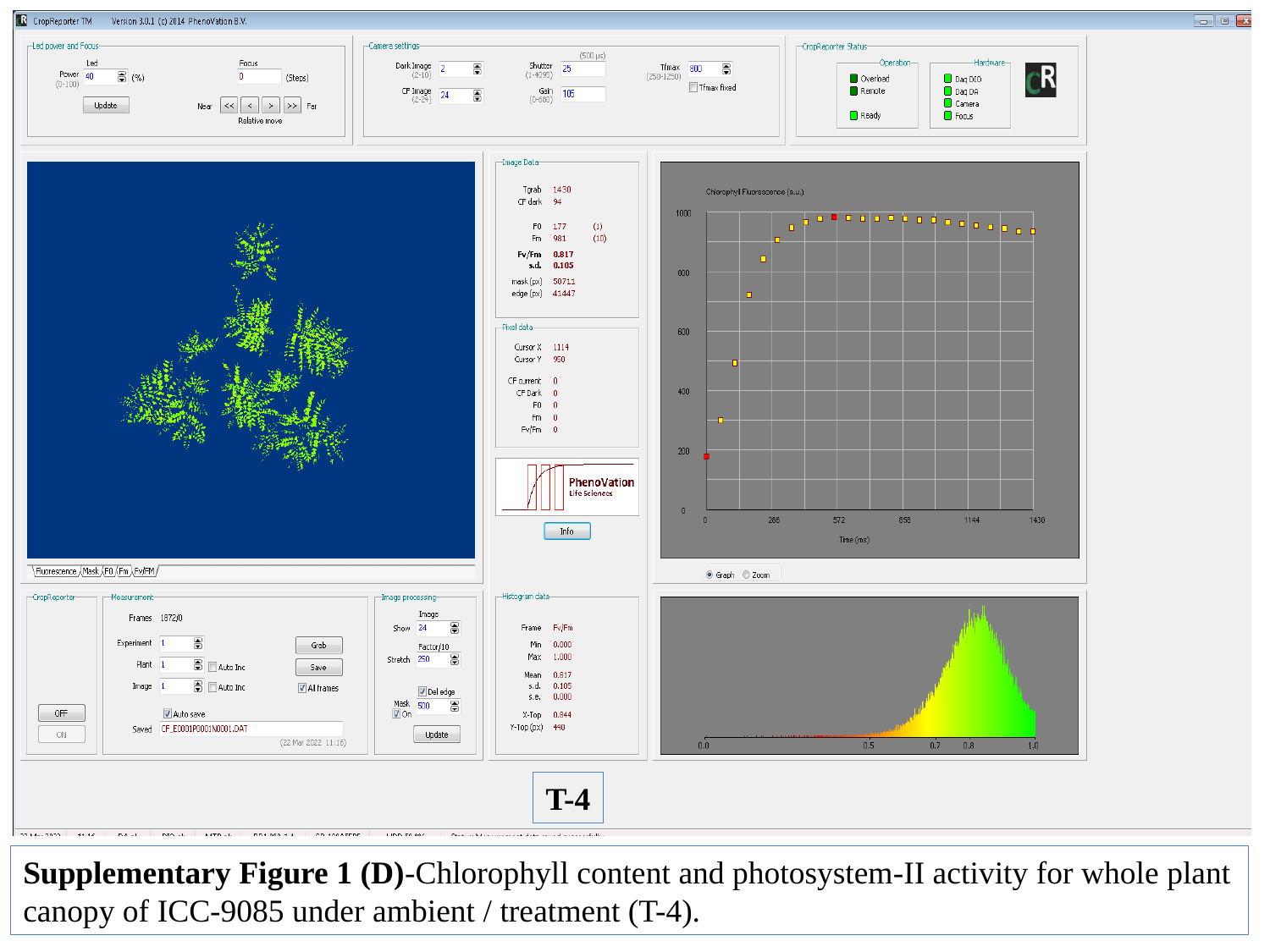

T-4
Supplementary Figure 1 (D)-Chlorophyll content and photosystem-II activity for whole plant canopy of ICC-9085 under ambient / treatment (T-4).

## Slide 5
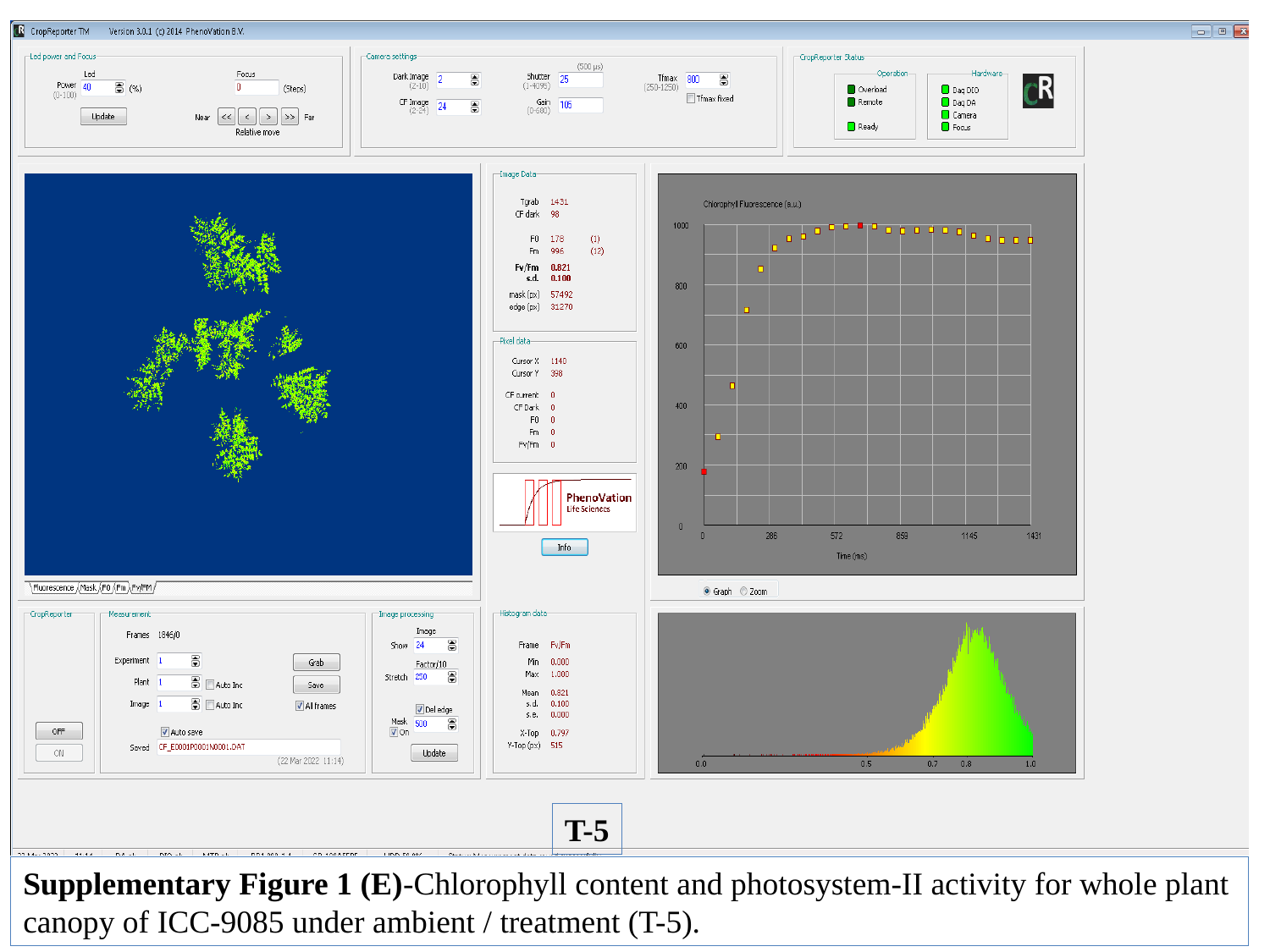

T-5
Supplementary Figure 1 (E)-Chlorophyll content and photosystem-II activity for whole plant canopy of ICC-9085 under ambient / treatment (T-5).

## Slide 6
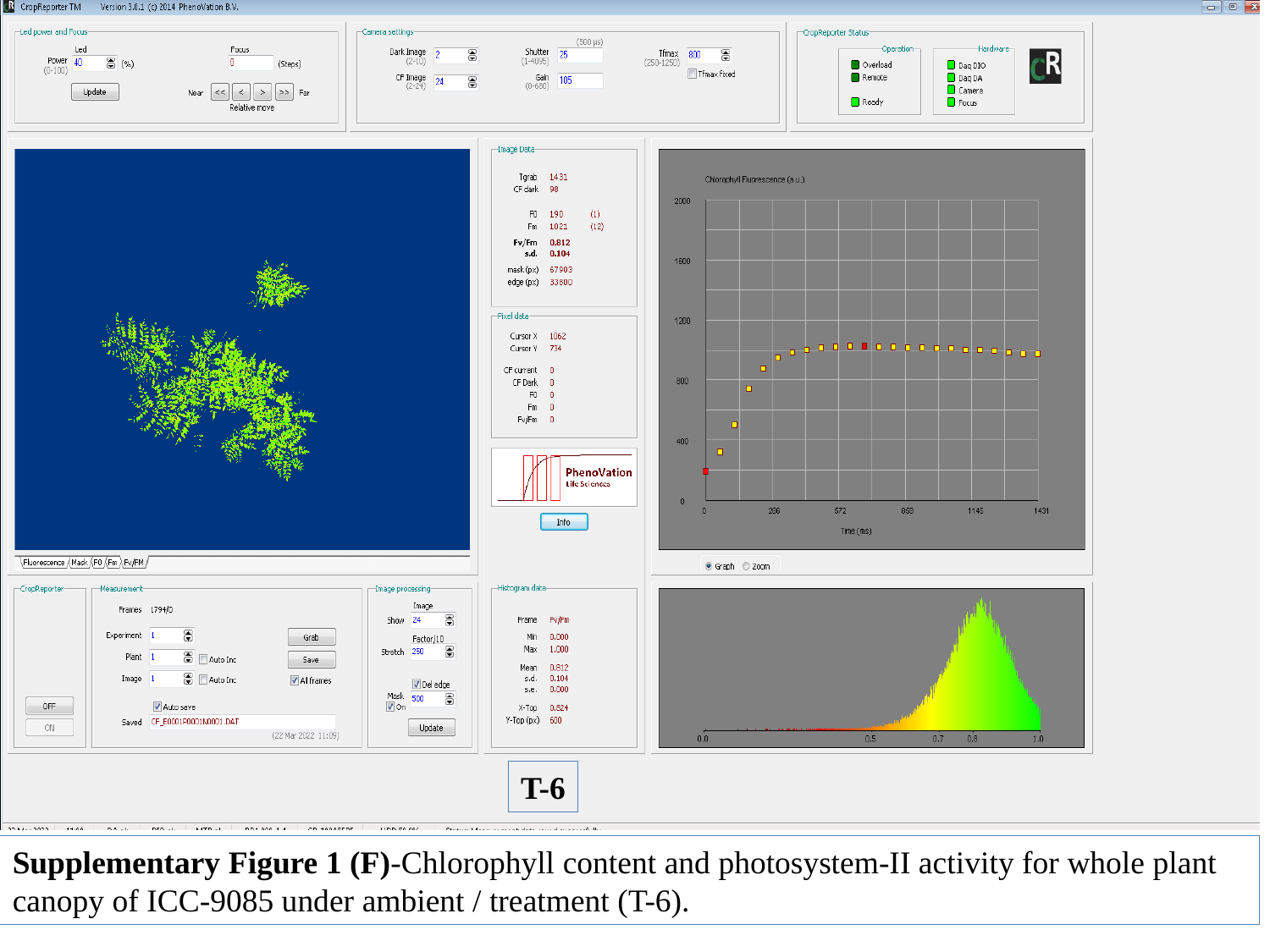

T-6
Supplementary Figure 1 (F)-Chlorophyll content and photosystem-II activity for whole plant canopy of ICC-9085 under ambient / treatment (T-6).

## Slide 7
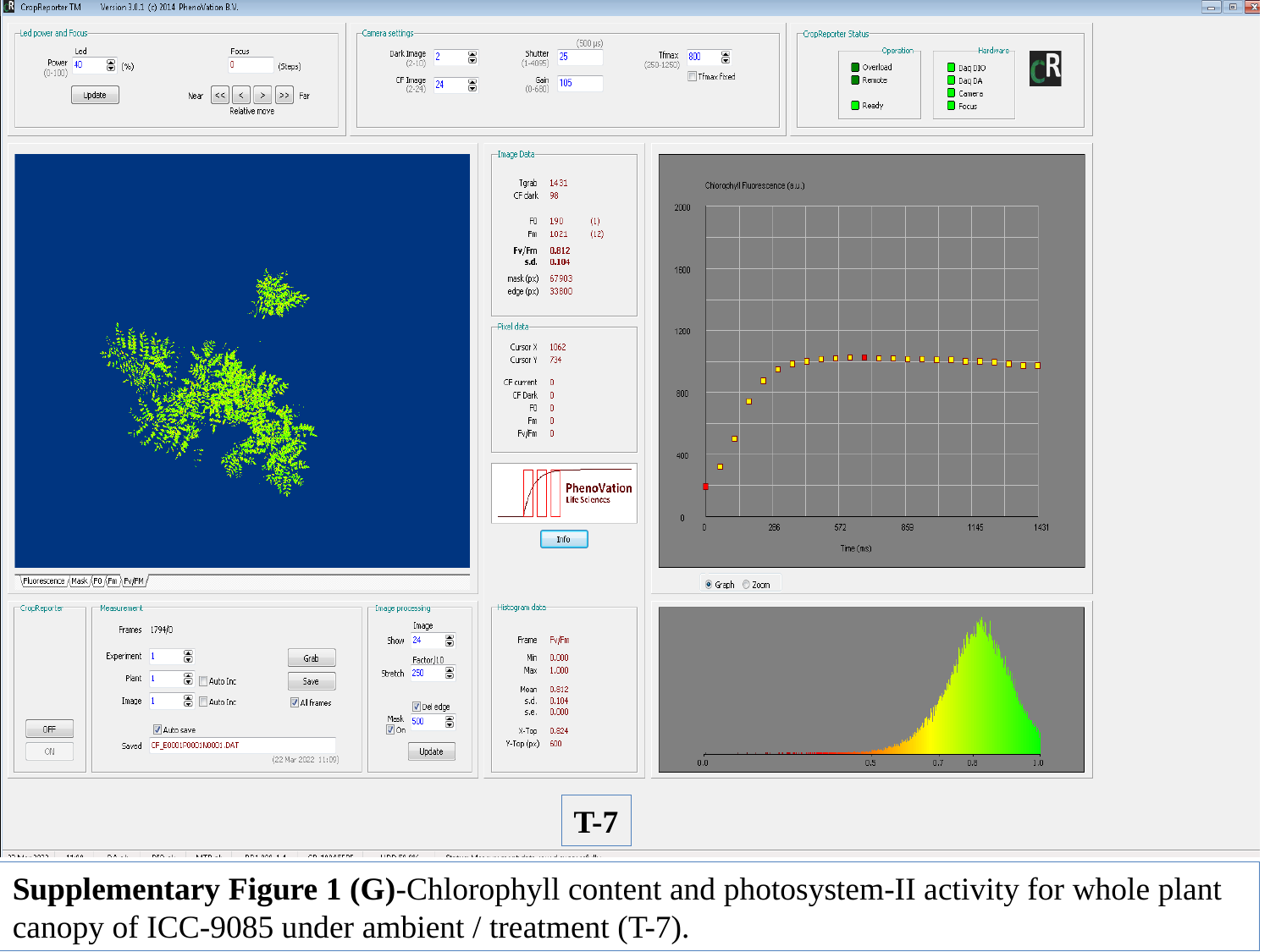

T-7
Supplementary Figure 1 (G)-Chlorophyll content and photosystem-II activity for whole plant canopy of ICC-9085 under ambient / treatment (T-7).

## Slide 8
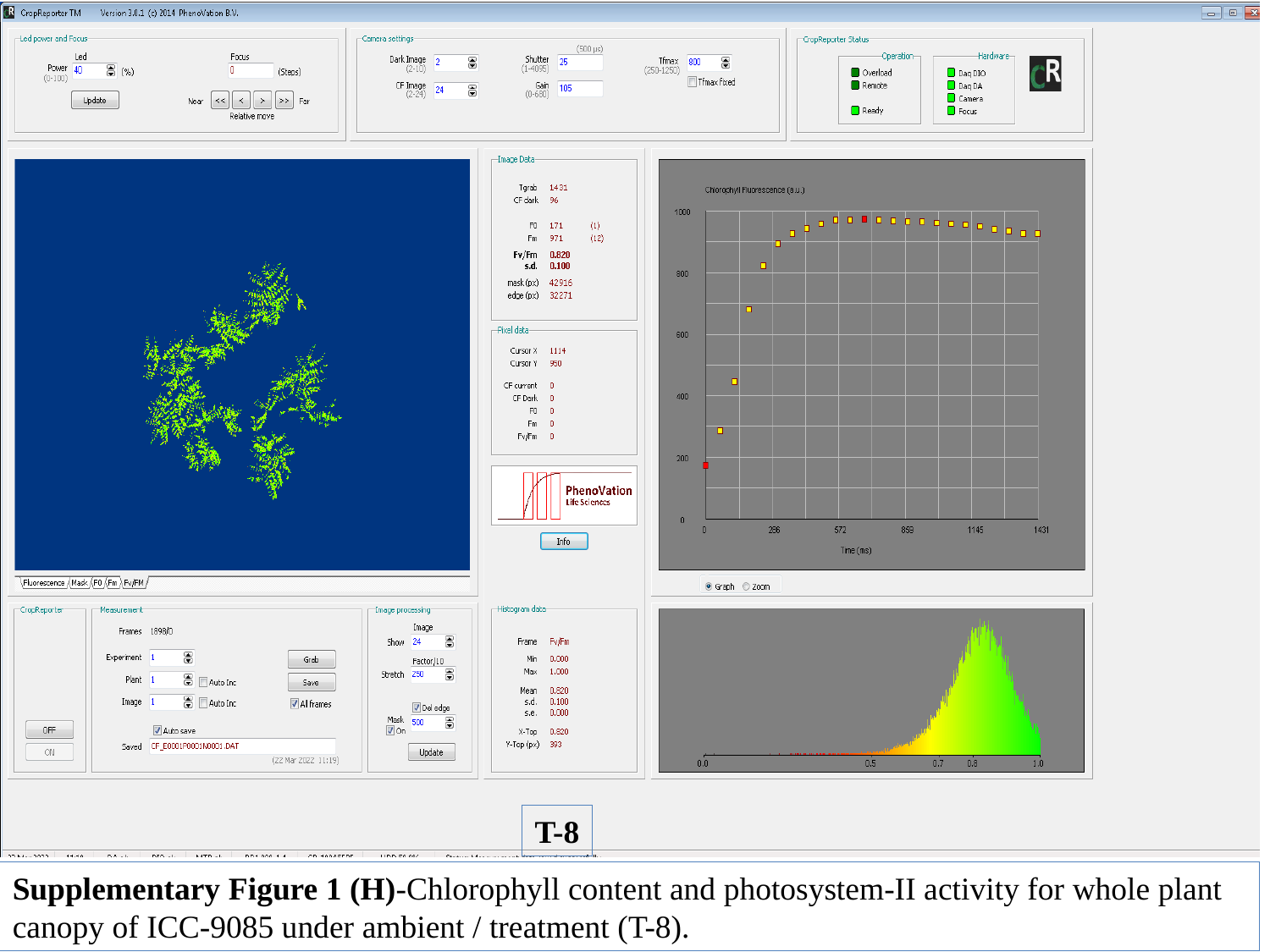

T-8
Supplementary Figure 1 (H)-Chlorophyll content and photosystem-II activity for whole plant canopy of ICC-9085 under ambient / treatment (T-8).

## Slide 9
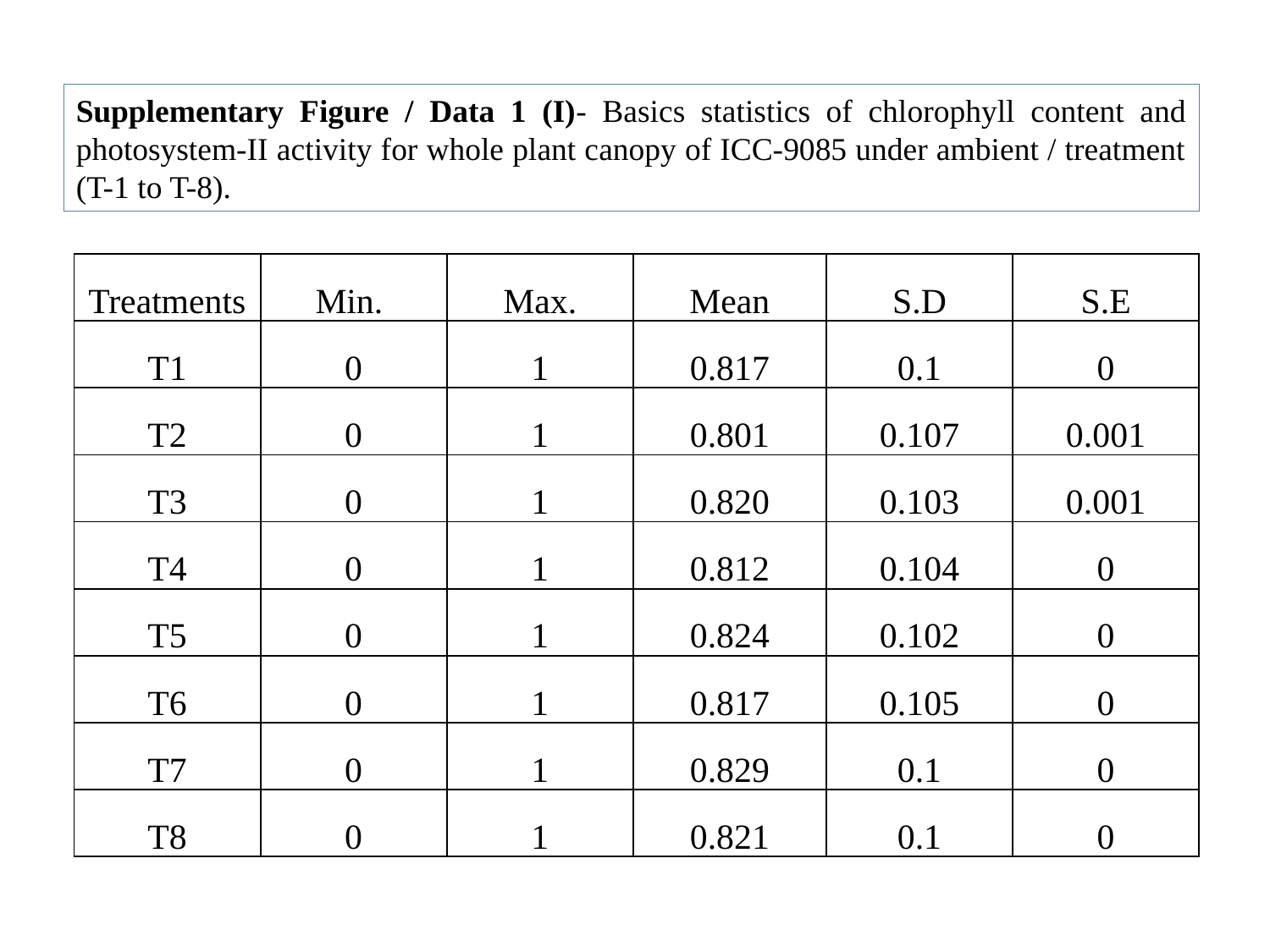

Supplementary Figure / Data 1 (I)- Basics statistics of chlorophyll content and photosystem-II activity for whole plant canopy of ICC-9085 under ambient / treatment (T-1 to T-8).
| Treatments | Min. | Max. | Mean | S.D | S.E |
| --- | --- | --- | --- | --- | --- |
| T1 | 0 | 1 | 0.817 | 0.1 | 0 |
| T2 | 0 | 1 | 0.801 | 0.107 | 0.001 |
| T3 | 0 | 1 | 0.820 | 0.103 | 0.001 |
| T4 | 0 | 1 | 0.812 | 0.104 | 0 |
| T5 | 0 | 1 | 0.824 | 0.102 | 0 |
| T6 | 0 | 1 | 0.817 | 0.105 | 0 |
| T7 | 0 | 1 | 0.829 | 0.1 | 0 |
| T8 | 0 | 1 | 0.821 | 0.1 | 0 |

## Slide 10
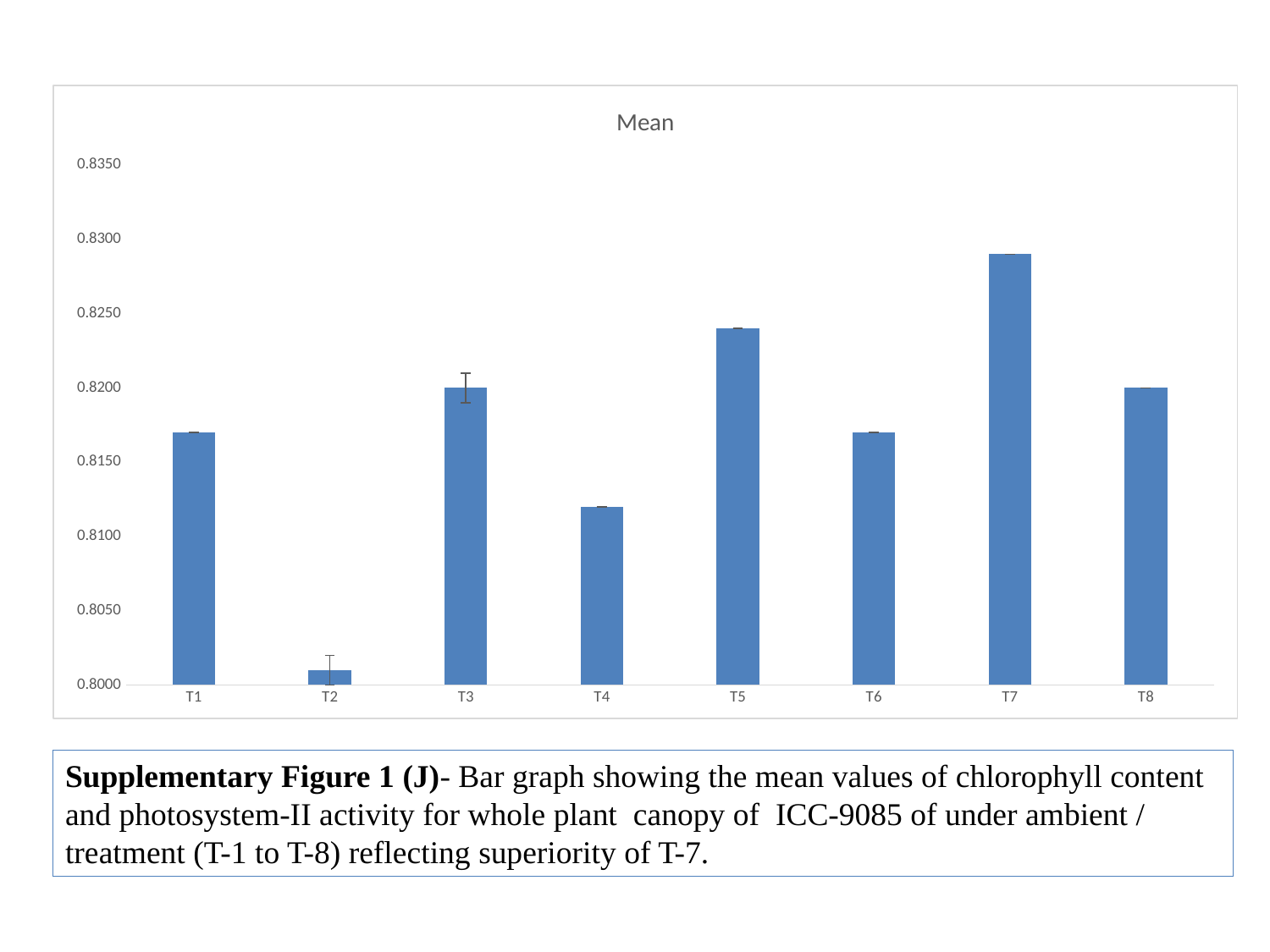

### Chart:
| Category | Mean |
|---|---|
| T1 | 0.817 |
| T2 | 0.801 |
| T3 | 0.8200000000000002 |
| T4 | 0.8120000000000003 |
| T5 | 0.824 |
| T6 | 0.817 |
| T7 | 0.8290000000000002 |
| T8 | 0.8200000000000002 |Supplementary Figure 1 (J)- Bar graph showing the mean values of chlorophyll content and photosystem-II activity for whole plant canopy of ICC-9085 of under ambient / treatment (T-1 to T-8) reflecting superiority of T-7.
