# Supplementary material for: Symbiotic nitrogen fixation for sustainable chickpea yield and prospects for genome editing in changing climatic situations
Source: Front Plant Sci. 2025 Sep 1;16:1621191. doi: 10.3389/fpls.2025.1621191 (PMC12433944; doi:10.3389/fpls.2025.1621191)
Supplement: Supplementary file 3 [file Table1.docx]

**SUPPLEMENTARY TABLE-1: SOIL PROFILE DESCRIPTIONS**

| **S. No.** | **Soil Characteristics** | **Characteristics Category** | **Value Range** | **S.**  **No.** | **Soil Characteristics** | **Characteristics Category** | **Value Range** |
| --- | --- | --- | --- | --- | --- | --- | --- |
| 1. | PH | Mildly Alkaline | 7.5-8.5 | 7. | S (mg/kg) | Medium | 10.0-20.0 |
| 2. | EC (ds/m) | Low | 0.4-0.6 | 8. | DTPA_Zn (mg/kg) | Adequate | 1.0-5.0 |
| 3. | O.C. (%) | Low | <0.5 | 9. | DTPA_Fe (mg/kg) | Adequate | 5.8-10 |
| 4. | N (kg/ha) | Low | <280 | 10. | DTPA_Mn (mg/kg) | Adequate | 10.0-25.0 |
| 5. | P (kg/ha) | High | 25-50 | 11. | DTPA_Cu (mg/kg) | Adequate | 0.5-10 |
| 6. | K (kg/ha) | High | >280 |  | - | - | - |

This table presents the categorized physico-chemical and micronutrient characteristics of the soil samples. The soil pH falls within the mildly alkaline range (7.5–8.5), indicating slightly basic conditions, while electrical conductivity (EC) is low (0.4–0.6 dS/m), suggesting minimal salinity stress. The organic carbon (O.C.) content is low (<0.5%), reflecting limited organic matter and potential constraints on soil fertility. Available nitrogen (N) is also low (<280 kg/ha), indicating a need for nitrogen supplementation. In contrast, phosphorus (P) and potassium (K) levels are high, ranging from 25–50 kg/ha and >280 kg/ha, respectively, suggesting adequate availability of these macronutrients. Among the micronutrients, sulfur (S) falls in the medium range (10.0–20.0 mg/kg), while DTPA-extractable zinc (Zn: 1.0–5.0 mg/kg), iron (Fe: 5.8–10.0 mg/kg), manganese (Mn: 10.0–25.0 mg/kg), and copper (Cu: 0.5–10.0 mg/kg) are all present in adequate concentrations. Overall, the soil exhibits a balanced micronutrient profile but shows deficiencies in nitrogen and organic carbon, which may require appropriate management interventions for optimal crop productivity are properly mentioned in **Supplementary Table 1**
