# Supplementary material for: Symbiotic nitrogen fixation for sustainable chickpea yield and prospects for genome editing in changing climatic situations
Source: Front Plant Sci. 2025 Sep 1;16:1621191. doi: 10.3389/fpls.2025.1621191 (PMC12433944; doi:10.3389/fpls.2025.1621191)
Supplement: Supplementary file 4 [file Table2.docx]

**SUPPLEMENTARY TABLE 2: METROLOGICAL DATA FOR THE CROP SEASONS 2020-21 & 2021-22**

| **Date** | **Max. Temp (^o^C)** | **Min. Temp (^o^C)** | **Rainfall (mm)** | **Wind Speed (kmph)** | **Wind Direction-I** | **Wind Direction-II** | **Weather condition**  (Code 0-9) | **Weather condition-II**  (Code 0-9) | **RH-I**  **(%)** | **RH-II (%)** | **BSS** (hrs.) | **Evaporation (mm)** |
| --- | --- | --- | --- | --- | --- | --- | --- | --- | --- | --- | --- | --- |
| **Year 2020-21** | | | | | | | | | | | | |
| 1-Oct-20 | 35.0 | 22.0 | 0.0 | 5.4 | C | NW | 1.0 | 0.0 | 78.0 | 41.0 | 9.0 | 6.0 |
| 2-Oct-20 | 36.0 | 20.0 | 0.0 | 7.8 | C | NW | 0.0 | 0.0 | 75.0 | 52.0 | 9.0 | 6.2 |
| 3-Oct-20 | 35.4 | 20.0 | 0.0 | 1.8 | C | NW | 0.0 | 0.0 | 86.0 | 37.0 | 8.6 | 5.8 |
| 4-Oct-20 | 35.4 | 19.6 | 0.0 | 3.1 | C | NW | 0.0 | 0.0 | 69.0 | 46.0 | 8.7 | 6.0 |
| 5-Oct-20 | 35.0 | 19.0 | 0.0 | 2.9 | C | NW | 0.0 | 0.0 | 75.0 | 50.0 | 8.8 | 5.6 |
| 6-Oct-20 | 35.0 | 18.8 | 0.0 | 4.6 | C | WNW | 0.0 | 0.0 | 73.0 | 52.0 | 9.3 | 6.0 |
| 7-Oct-20 | 36.0 | 19.0 | 0.0 | 4.2 | C | NW | 0.0 | 0.0 | 77.0 | 42.0 | 8.9 | 6.2 |
| 8-Oct-20 | 35.4 | 19.0 | 0.0 | 3.6 | C | NW | 0.0 | 0.0 | 83.0 | 45.0 | 8.4 | 5.8 |
| 9-Oct-20 | 34.8 | 19.4 | 0.0 | 3.2 | C | NW | 1.0 | 0.0 | 85.0 | 42.0 | 8.4 | 5.6 |
| 10-Oct-20 | 34.8 | 19.2 | 0.0 | 1.9 | C | WNW | 0.0 | 0.0 | 72.0 | 41.0 | 8.0 | 5.4 |
| 11-Oct-20 | 34.8 | 19.3 | 0.0 | 3.1 | C | NW | 1.0 | 0.0 | 75.0 | 42.0 | 7.7 | 4.9 |
| 12-Oct-20 | 34.6 | 19.5 | 0.0 | 3.0 | C | NW | 0.0 | 0.0 | 87.0 | 39.0 | 8.0 | 5.6 |
| 13-Oct-20 | 34.8 | 19.4 | 0.0 | 1.8 | C | SE | 1.0 | 1.0 | 86.0 | 41.0 | 7.2 | 5.3 |
| 14-Oct-20 | 35.0 | 23.0 | 0.0 | 1.9 | C | C | 1.0 | 1.0 | 85.0 | 41.0 | 3.7 | 4.6 |
| 15-Oct-20 | 34.2 | 20.0 | 0.0 | 1.5 | C | NW | 1.0 | 0.0 | 86.0 | 39.0 | 0.0 | 4.0 |
| 16-Oct-20 | 35.0 | 17.0 | 0.0 | 2.0 | C | NW | 1.0 | 0.0 | 81.0 | 33.0 | 7.3 | 5.0 |
| 17-Oct-20 | 34.0 | 17.2 | 0.0 | 1.3 | C | SW | 1.0 | 0.0 | 83.0 | 37.0 | 7.3 | 4.4 |
| 18-Oct-20 | 34.0 | 17.0 | 0.0 | 1.1 | C | NW | 1.0 | 0.0 | 79.0 | 33.0 | 6.7 | 4.0 |
| 19-Oct-20 | 34.0 | 16.5 | 0.0 | 2.9 | C | NW | 1.0 | 0.0 | 80.0 | 35.0 | 8.0 | 4.2 |
| 20-Oct-20 | 33.4 | 13.5 | 0.0 | 3.0 | C | NW | 1.0 | 0.0 | 80.0 | 24.0 | 9.0 | 4.4 |
| 21-Oct-20 | 34.0 | 12.5 | 0.0 | 2.2 | C | N | 1.0 | 0.0 | 87.0 | 23.0 | 8.9 | 4.1 |
| 22-Oct-20 | 34.9 | 15.6 | 0.0 | 1.9 | C | C | 1.0 | 1.0 | 82.0 | 29.0 | 8.3 | 4.3 |
| 23-Oct-20 | 32.7 | 12.6 | 0.0 | 0.9 | C | SW | 1.0 | 1.0 | 83.0 | 32.0 | 1.5 | 3.8 |
| 24-Oct-20 | 33.0 | 12.8 | 0.0 | 1.3 | C | SW | 1.0 | 1.0 | 89.0 | 32.0 | 7.2 | 3.6 |
| 25-Oct-20 | 32.1 | 12.9 | 0.0 | 2.6 | C | W | 0.0 | 0.0 | 93.0 | 39.0 | 6.6 | 3.4 |
| 26-Oct-20 | 33.6 | 12.4 | 0.0 | 2.2 | C | N | 0.0 | 1.0 | 82.0 | 28.0 | 6.3 | 3.1 |
| 27-Oct-20 | 32.2 | 13.4 | 0.0 | 1.4 | C | N | 1.0 | 0.0 | 89.0 | 25.0 | 0.8 | 3.6 |
| 28-Oct-20 | 30.9 | 10.9 | 0.0 | 2.7 | C | N | 0.0 | 1.0 | 82.0 | 35.0 | 7.6 | 2.6 |
| 29-Oct-20 | 30.8 | 10.8 | 0.0 | 1.0 | C | C | 4.0 | 1.0 | 93.0 | 31.0 | 2.6 | 2.2 |
| 30-Oct-20 | 31.0 | 11.6 | 0.0 | 1.3 | C | N | 4.0 | 0.0 | 93.0 | 33.0 | 4.4 | 2.1 |
| 31-Oct-20 | 30.4 | 11.4 | 0.0 | 1.8 | C | NW | 4.0 | 0.0 | 95.0 | 28.0 | 7.2 | 3.0 |
| 1-Nov-20 | 30.2 | 10.2 | 0.0 | 3.2 | C | NW | 4.0 | 0.0 | 88.0 | 25.0 | 6.5 | 3.2 |
| 2-Nov-20 | 29.0 | 11.0 | 0.0 | 3.9 | C | NW | 1.0 | 1.0 | 73.0 | 25.0 | 5.4 | 3.7 |
| 3-Nov-20 | 29.0 | 10.0 | 0.0 | 2.3 | C | NW | 1.0 | 0.0 | 84.0 | 26.0 | 1.4 | 3.5 |
| 4-Nov-20 | 31.0 | 11.4 | 0.0 | 2.2 | C | W | 1.0 | 1.0 | 80.0 | 33.0 | 6.4 | 3.2 |
| 5-Nov-20 | 28.0 | 10.7 | 0.0 | 1.8 | C | W | 1.0 | 1.0 | 85.0 | 28.0 | 0.0 | 3.0 |
| 6-Nov-20 | 30.0 | 11.0 | 0.0 | 2.1 | C | NW | 1.0 | 0.0 | 88.0 | 26.0 | 4.8 | 3.4 |
| 7-Nov-20 | 30.2 | 11.0 | 0.0 | 1.9 | C | NW | 1.0 | 0.0 | 85.0 | 29.0 | 1.0 | 2.8 |
| 8-Nov-20 | 29.2 | 11.0 | 0.0 | 2.4 | C | C | 1.0 | 1.0 | 83.0 | 33.0 | 4.0 | 3.0 |
| 9-Nov-20 | 28.4 | 9.1 | 0.0 | 0.2 | C | C | 4.0 | 4.0 | 90.0 | 38.0 | 0.4 | 2.9 |
| 10-Nov-20 | 26.8 | 9.8 | 0.0 | 2.0 | C | E | 4.0 | 1.0 | 88.0 | 33.0 | 0.0 | 2.1 |
| 11-Nov-20 | 28.5 | 11.0 | 0.0 | 0.9 | C | SE | 1.0 | 0.0 | 86.0 | 37.0 | 0.8 | 2.4 |
| 12-Nov-20 | 28.4 | 10.8 | 0.0 | 1.8 | C | S | 1.0 | 0.0 | 88.0 | 41.0 | 3.6 | 2.9 |
| 13-Nov-20 | 28.2 | 11.0 | 0.0 | 0.9 | C | C | 1.0 | 0.0 | 90.0 | 36.0 | 2.6 | 3.4 |
| 14-Nov-20 | 28.5 | 9.4 | 0.0 | 1.3 | C | SW | 4.0 | 1.0 | 85.0 | 37.0 | 2.8 | 2.6 |
| 15-Nov-20 | 27.8 | 11.8 | 0.0 | 0.6 | C | SE | 4.0 | 4.0 | 80.0 | 42.0 | 0.0 | 2.9 |
| 16-Nov-20 | 27.0 | 13.2 | 3.2 | 3.5 | C | SE | 4.0 | 1.0 | 76.0 | 67.0 | 0.0 | 2.2 |
| 17-Nov-20 | 24.0 | 11.8 | 0.0 | 1.5 | C | NW | 4.0 | 0.0 | 79.0 | 53.0 | 1.7 | 3.0 |
| 18-Nov-20 | 25.5 | 10.8 | 0.0 | 2.8 | C | W | 4.0 | 0.0 | 77.0 | 35.0 | 6.4 | 3.3 |
| 19-Nov-20 | 25.0 | 8.6 | 0.0 | 2.2 | C | NW | 4.0 | 0.0 | 90.0 | 33.0 | 5.5 | 3.1 |
| 20-Nov-20 | 24.4 | 7.0 | 0.0 | 1.8 | C | W | 4.0 | 0.0 | 79.0 | 34.0 | 6.1 | 2.6 |
| 21-Nov-20 | 24.8 | 10.6 | 0.0 | 4.9 | W | N | 1.0 | 0.0 | 88.0 | 30.0 | 5.8 | 3.2 |
| 22-Nov-20 | 24.0 | 5.5 | 0.0 | 3.6 | N | N | 4.0 | 0.0 | 95.0 | 36.0 | 5.2 | 2.2 |
| 23-Nov-20 | 23.6 | 7.0 | 0.0 | 2.5 | C | W | 1.0 | 1.0 | 74.0 | 31.0 | 4.5 | 2.0 |
| 24-Nov-20 | 25.4 | 6.0 | 0.0 | 0.7 | C | E | 1.0 | 1.0 | 76.0 | 32.0 | 6.0 | 2.2 |
| 25-Nov-20 | 24.5 | 10.0 | 0.0 | 0.9 | C | C | 4.0 | 2.0 | 87.0 | 53.0 | 3.6 | 1.5 |
| 26-Nov-20 | 22.0 | 12.5 | 0.0 | 3.4 | NE | N | 1.0 | 0.0 | 83.0 | 40.0 | 0.0 | 1.5 |
| 27-Nov-20 | 25.4 | 11.5 | 0.0 | 4.0 | NW | NW | 0.0 | 0.0 | 78.0 | 30.0 | 7.0 | 2.5 |
| 28-Nov-20 | 26.2 | 10.8 | 0.0 | 3.7 | W | NW | 1.0 | 0.0 | 75.0 | 38.0 | 8.5 | 3.0 |
| 29-Nov-20 | 25.4 | 6.0 | 0.0 | 3.3 | C | NW | 0.0 | 0.0 | 81.0 | 31.0 | 8.4 | 3.4 |
| 30-Nov-20 | 25.4 | 10.0 | 0.0 | 1.4 | C | NW | 0.0 | 0.0 | 71.0 | 34.0 | 7.8 | 3.3 |
| 1-Dec-20 | 25.2 | 6.5 | 0.0 | 1.6 | C | W | 4.0 | 0.0 | 94.0 | 35.0 | 7.1 | 3.0 |
| 2-Dec-20 | 25.4 | 7.0 | 0.0 | 1.3 | C | NW | 4.0 | 0.0 | 94.0 | 32.0 | 5.0 | 1.9 |
| 3-Dec-20 | 26.2 | 7.4 | 0.0 | 2.1 | C | W | 4.0 | 0.0 | 89.0 | 48.0 | 6.6 | 2.2 |
| 4-Dec-20 | 26.2 | 8.2 | 0.0 | 1.4 | C | SW | 4.0 | 0.0 | 80.0 | 53.0 | 6.8 | 2.6 |
| 5-Dec-20 | 27.0 | 12.8 | 0.0 | 1.5 | C | S | 4.0 | 1.0 | 87.0 | 57.0 | 5.4 | 2.2 |
| 6-Dec-20 | 25.6 | 12.9 | 0.0 | 2.8 | C | C | 4.0 | 0.0 | 91.0 | 53.0 | 3.4 | 2.3 |
| 7-Dec-20 | 27.2 | 10.6 | 0.0 | 1.4 | C | W | 4.0 | 1.0 | 93.0 | 54.0 | 3.8 | 2.5 |
| 8-Dec-20 | 25.4 | 8.4 | 0.0 | 2.2 | C | C | 4.0 | 0.0 | 92.0 | 36.0 | 3.6 | 1.9 |
| 9-Dec-20 | 27.2 | 8.3 | 0.0 | 0.9 | C | N | 4.0 | 1.0 | 95.0 | 36.0 | 6.8 | 2.8 |
| 10-Dec-20 | 28.0 | 9.6 | 0.0 | 2.8 | C | S | 4.0 | 0.0 | 86.0 | 57.0 | 6.9 | 2.1 |
| 11-Dec-20 | 26.7 | 9.9 | 0.0 | 0.8 | C | C | 4.0 | 1.0 | 93.0 | 78.0 | 5.5 | 2.0 |
| 12-Dec-20 | 25.0 | 14.6 | 0.6 | 0.5 | C | N | 4.0 | 1.0 | 90.0 | 66.0 | 0.8 | 1.5 |
| 13-Dec-20 | 21.2 | 9.7 | 0.0 | 3.0 | C | C | 4.0 | 0.0 | 97.0 | 54.0 | 0.0 | 1.4 |
| 14-Dec-20 | 22.9 | 7.7 | 0.0 | 1.6 | W | NW | 2.0 | 0.0 | 90.0 | 54.0 | 5.9 | 1.9 |
| 15-Dec-20 | 18.0 | 3.6 | 0.0 | 7.9 | NW | N | 4.0 | 1.0 | 92.0 | 59.0 | 6.7 | 1.6 |
| 16-Dec-20 | 18.0 | 16.2 | 0.0 | 7.3 | NW | SW | 4.0 | 0.0 | 97.0 | 52.0 | 3.7 | 1.3 |
| 17-Dec-20 | 17.4 | 3.4 | 0.0 | 2.8 | W | W | 4.0 | 0.0 | 84.0 | 57.0 | 5.6 | 1.6 |
| 18-Dec-20 | 14.6 | 4.6 | 0.0 | 5.6 | W | W | 4.0 | 0.0 | 74.0 | 38.0 | 6.0 | 1.7 |
| 19-Dec-20 | 19.4 | 2.5 | 0.0 | 4.1 | C | NW | 4.0 | 0.0 | 78.0 | 42.0 | 8.2 | 2.0 |
| 20-Dec-20 | 21.2 | 2.4 | 0.0 | 3.4 | C | W | 4.0 | 0.0 | 70.0 | 27.0 | 7.6 | 2.2 |
| 21-Dec-20 | 21.2 | 3.9 | 0.0 | 2.8 | C | W | 4.0 | 0.0 | 85.0 | 39.0 | 5.9 | 1.8 |
| 22-Dec-20 | 22.5 | 3.5 | 0.0 | 2.0 | C | NW | 4.0 | 0.0 | 94.0 | 36.0 | 5.9 | 2.3 |
| 23-Dec-20 | 22.6 | 2.6 | 0.0 | 1.2 | C | W | 4.0 | 0.0 | 93.0 | 43.0 | 5.5 | 2.0 |
| 24-Dec-20 | 23.0 | 2.9 | 0.0 | 1.2 | C | W | 4.0 | 0.0 | 93.0 | 48.0 | 4.6 | 1.7 |
| 25-Dec-20 | 22.0 | 2.6 | 0.0 | 1.3 | C | NW | 4.0 | 0.0 | 93.0 | 42.0 | 5.2 | 1.5 |
| 26-Dec-20 | 21.2 | 2.4 | 0.0 | 3.0 | C | NW | 4.0 | 0.0 | 90.0 | 48.0 | 6.6 | 1.6 |
| 27-Dec-20 | 20.2 | 4.4 | 0.0 | 2.0 | C | SE | 4.0 | 1.0 | 88.0 | 55.0 | 6.1 | 5.1 |
| 28-Dec-20 | 22.0 | 3.6 | 0.0 | 4.7 | C | NW | 4.0 | 0.0 | 94.0 | 34.0 | 2.9 | 1.7 |
| 29-Dec-20 | 19.0 | 3.5 | 0.0 | 3.6 | C | NW | 4.0 | 0.0 | 85.0 | 47.0 | 7.5 | 2.0 |
| 30-Dec-20 | 17.0 | 5.0 | 0.0 | 6.0 | NW | W | 4.0 | 0.0 | 94.0 | 64.0 | 7.2 | 1.6 |
| 31-Dec-20 | 16.0 | 2.6 | 0.0 | 4.2 | NW | W | 4.0 | 1.0 | 97.0 | 63.0 | 5.5 | 1.5 |
| 1-Jan-21 | 30.2 | 10 | 0 | 3.4 | C | W | 4 | 0 | 83 | 38 | 8.7 | 4.4 |
| 2-Jan-21 | 27 | 15 | 0 | 7.6 | NW | W | 1 | 0 | 70 | 29 | 9.3 | 3.8 |
| 3-Jan-21 | 27.8 | 10.6 | 0 | 4.2 | C | W | 4 | 0 | 82 | 27 | 9.7 | 4.7 |
| 4-Jan-21 | 30 | 9.9 | 0 | 7.7 | C | C | 4 | 0 | 89 | 21 | 9.5 | 5.1 |
| 5-Jan-21 | 33 | 11.4 | 0 | 1.8 | C | W | 4 | 0 | 85 | 31 | 8.6 | 5 |
| 6-Jan-21 | 30 | 13 | 0 | 7.9 | C | N | 4 | 0 | 78 | 28 | 8.8 | 4.4 |
| 7-Jan-21 | 30 | 13.5 | 0 | 3.4 | C | SE | 0 | 0 | 83 | 34 | 8.9 | 4.5 |
| 8-Jan-21 | 32 | 13.8 | 0 | 6 | SE | SE | 1 | 0.0 | 72 | 29 | 7.7 | 3.8 |
| 9-Jan-21 | 30.6 | 17.2 | 0 | 3.6 | N | SE | 1 | 0 | 77 | 43 | 5.9 | 3.6 |
| 10-Jan-21 | 32.1 | 16.4 | 0.3 | 4.7 | C | W | 4 | 0 | 83 | 36 | 5.2 | 3.8 |
| 11-Jan-21 | 32.4 | 15.2 | 0 | 1.7 | C | E | 4 | 0 | 88 | 28 | 7.1 | 3.6 |
| 12-Jan-21 | 33.6 | 15.9 | 0 | 2.4 | C | SE | 6 | 2 | 74 | 45 | 6.6 | 3.4 |
| 13-Jan-21 | 24.8 | 13.1 | 0 | 2.1 | C | N | 0 | 0 | 83 | 38 | 1.8 | 2.6 |
| 14-Jan-21 | 30.2 | 13.2 | 0 | 6.3 | C | N | 0 | 0 | 98 | 35 | 7.1 | 4 |
| 15-Jan-21 | 30.7 | 13.6 | 0 | 3.7 | C | NW | 4 | 0 | 86 | 37 | 8.5 | 4.4 |
| 16-Jan-21 | 30.9 | 16.1 | 0 | 4.3 | C | W | 4 | 0.0 | 83 | 36 | 3.7 | 4.2 |
| 17-Jan-21 | 31.3 | 15 | 0 | 4 | C | NW | 4 | 0 | 86 | 30 | 0 | 4.1 |
| 18-Jan-21 | 34 | 14.2 | 0 | 3 | C | W | 4 | 0 | 88 | 32 | 5 | 4.2 |
| 19-Jan-21 | 34.1 | 18.4 | 0 | 2.1 | C | NW | 0 | 0 | 80 | 36 | 0.7 | 4 |
| 20-Jan-21 | 34 | 14.8 | 0 | 2.3 | C | NNW | 4 | 0 | 86 | 32 | 6.3 | 4.2 |
| 21-Jan-21 | 33 | 14.4 | 0 | 3.3 | C | E | 0 | 1 | 86 | 33 | 7.8 | 3.9 |
| 22-Jan-21 | 35.4 | 15.5 | 0 | 5.2 | E | NE | 1 | 1 | 67 | 44 | 7.8 | 4 |
| 23-Jan-21 | 32 | 20.4 | 0 | 10.2 | NW | NW | 2 | 2 | 63 | 75 | 0 | 4 |
| 24-Jan-21 | 29 | 14 | 0 | 6.3 | C | C | 1 | 0.0 | 80 | 31 | 1.8 | 3.3 |
| 25-Jan-21 | 32 | 16.4 | 0 | 2.1 | NW | N | 1 | 0 | 72 | 36 | 6.2 | 4.1 |
| 26-Jan-21 | 29.0 | 16.0 | 0.0 | 7.4 | NNW | NW | 1.0 | 0 | 79 | 36 | 5.7 | 3.6 |
| 27-Jan-21 | 32 | 14 | 0 | 4.8 | C | W | 1 | 0 | 79 | 29 | 8.6 | 4 |
| 28-Jan-21 | 34.4 | 16.2 | 0 | 2.9 | C | W | 0 | 0 | 80 | 39 | 8.4 | 4.5 |
| 29-Jan-21 | 37.2 | 20.6 | 0 | 3 | C | S | 1 | 0 | 68 | 36 | 8.4 | 4.6 |
| 30-Jan-21 | 40 | 16.5 | 0 | 3.1 | C | NW | 0 | 2 | 75 | 15 | 7.8 | 5.4 |
| 31-Jan-21 | 38 | 19.4 | 0 | 13.3 | W | NW | 4 | 0 | 88 | 40 | 6.3 | 5.5 |
| 1-Feb-21 | 34.8 | 17.5 | 0 | 10.6 | W | NW | 0 | 0 | 65 | 43 | 8.8 | 5.4 |
| 2-Feb-21 | 34 | 15.2 | 0 | 9.8 | W | NW | 0 | 0 | 64 | 40 | 9.2 | 5.6 |
| 3-Feb-21 | 34 | 10.2 | 0 | 4.9 | C | SE | 0 | 0 | 59 | 49 | 9.5 | 5.6 |
| 4-Feb-21 | 35 | 13.6 | 0 | 2.9 | C | W | 0 | 0 | 78 | 38 | 9.5 | 6 |
| 5-Feb-21 | 37 | 17.6 | 0 | 3.2 | C | C | 0 | 1.0 | 67 | 11 | 10 | 6.4 |
| 6-Feb-21 | 38.6 | 20.5 | 0 | 4.3 | SE | N | 1 | 0 | 72 | 21 | 8 | 5.4 |
| 7-Feb-21 | 37 | 21.2 | 0 | 5.8 | C | C | 1 | 1 | 83 | 22 | 5.9 | 4.7 |
| 8-Feb-21 | 36.4 | 19.8 | 0 | 5.3 | C | NW | 0 | 0 | 70 | 23 | 8.8 | 5.8 |
| 9-Feb-21 | 35.2 | 19.5 | 0 | 3.7 | NW | NW | 0 | 0 | 75 | 22 | 10.5 | 6.2 |
| 10-Feb-21 | 36.2 | 15.5 | 0 | 3.8 | C | W | 0 | 0 | 67 | 27 | 10.1 | 6.6 |
| 11-Feb-21 | 37 | 17.5 | 0 | 4.6 | C | W | 0 | 0 | 67 | 24 | 10.3 | 6.6 |
| 12-Feb-21 | 39 | 17.2 | 0 | 3.4 | C | W | 1 | 0 | 62 | 25 | 9.5 | 7 |
| 13-Feb-21 | 39 | 18.2 | 0 | 3.7 | C | NW | 0 | 0 | 66 | 16 | 8 | 6.2 |
| 14-Feb-21 | 39.7 | 20.2 | 0 | 6.1 | C | NW | 0 | 0 | 50 | 23 | 9.1 | 7.2 |
| 15-Feb-21 | 39.9 | 20.4 | 0 | 5 | C | NW | 0 | 0 | 71 | 30 | 9.1 | 7.1 |
| 16-Feb-21 | 40 | 19 | 0 | 1 | C | S | 0 | 1.0 | 55 | 35 | 8.8 | 8.1 |
| 17-Feb-21 | 39.7 | 17 | 5.2 | 11.5 | S | S | 1 | 0 | 94 | 25 | 7.7 | 6.6 |
| 18-Feb-21 | 39 | 17.4 | 0 | 6.5 | C | C | 0 | 0 | 77 | 27 | 9.5 | 7.9 |
| 19-Feb-21 | 35.6 | 18.2 | 0 | 3.6 | C | NW | 0 | 0.0 | 69 | 36 | 9.8 | 6.5 |
| 20-Feb-21 | 38 | 20.8 | 0 | 8.3 | E | SE | 1 | 1 | 64 | 42 | 10.1 | 6.6 |
| 21-Feb-21 | 33.6 | 18.5 | 0 | 4.4 | W | W | 1 | 0 | 75 | 55 | 1.1 | 5.8 |
| 22-Feb-21 | 32.7 | 18 | 0 | 6.5 | N | W | 0 | 0 | 71 | 46 | 8.3 | 7.1 |
| 23-Feb-21 | 32.8 | 22.4 | 0.2 | 7 | SE | NE | 1 | 0 | 63 | 53 | 10 | 7 |
| 24-Feb-21 | 31.6 | 16.6 | 0 | 7.4 | NNW | NW | 0 | 0 | 72 | 53 | 6.4 | 6.7 |
| 25-Feb-21 | 33.8 | 15.9 | 0 | 6.1 | N | C | 0 | 0 | 75 | 27 | 11.6 | 8.4 |
| 26-Feb-21 | 37 | 14.6 | 0 | 4.7 | C | N | 0 | 0 | 56 | 26 | 11.1 | 7.2 |
| 27-Feb-21 | 39.3 | 19.6 | 0 | 3.1 | C | C | 0 | 0.0 | 59 | 29 | 9.7 | 7.6 |
| 28-Feb-21 | 41.4 | 21.2 | 0 | 3.5 | C | W | 0 | 0 | 59 | 32 | 8.9 | 8 |
| 1-Mar-21 | 42 | 22.4 | 0 | 4.2 | C | W | 1 | 0 | 67 | 32 | 7.6 | 8.4 |
| 2-Mar-21 | 42 | 22 | 0 | 4.6 | C | N | 0 | 0 | 69 | 33 | 6.3 | 8.6 |
| 3-Mar-21 | 39.8 | 27 | 0 | 4.1 | C | NW | 1 | 0 | 61 | 29 | 5.5 | 9.1 |
| 4-Mar-21 | 40 | 24.2 | 0 | 4.1 | C | W | 0 | 0 | 51 | 40 | 6.3 | 9.4 |
| 5-Mar-21 | 39.8 | 24 | 0 | 6.1 | E | W | 1 | 0 | 68 | 22 | 6.6 | 9.4 |
| 6-Mar-21 | 39.5 | 24.6 | 0 | 4.9 | S | SW | 0 | 0 | 64 | 45 | 9.3 | 9.8 |
| 7-Mar-21 | 40.5 | 25 | 0 | 5.5 | SE | NW | 0 | 0 | 61 | 44 | 3.3 | 9.4 |
| 8-Mar-21 | 41.8 | 27.2 | 0 | 4.5 | SE | E | 1 | 1 | 57 | 41 | 8.2 | 9.8 |
| 9-Mar-21 | 38.2 | 20.5 | 2.5 | 9 | N | E | 0 | 1 | 66 | 54 | 7.9 | 9.6 |
| 10-Mar-21 | 34.8 | 22.2 | 0 | 3.5 | C | N | 0 | 0 | 48 | 46 | 8.8 | 9.2 |
| 11-Mar-21 | 38.5 | 25 | 0 | 3.6 | E | NE | 0 | 1 | 61 | 28 | 10.7 | 9.1 |
| 12-Mar-21 | 38.2 | 23 | 0.2 | 5.1 | NW | E | 2 | 0 | 53 | 45 | 5.7 | 9.6 |
| 13-Mar-21 | 36 | 25.4 | 0 | 5.3 | SE | W | 0 | 0 | 69 | 48 | 4.6 | 9.3 |
| 14-Mar-21 | 40 | 23.5 | 0 | 5.4 | SE | NW | 0 | 0 | 54 | 49 | 10.5 | 9.6 |
| 15-Mar-21 | 36.6 | 20.6 | 3.4 | 7 | SE | NE | 2 | 0 | 80 | 47 | 8.5 | 9.2 |
| 16-Mar-21 | 33.6 | 21.8 | 0 | 5 | C | N | 1 | 0 | 75 | 39 | 8.4 | 9.5 |
| 17-Mar-21 | 36.4 | 22 | 0 | 4.1 | W | NW | 1 | 0 | 65 | 32 | 8 | 8.8 |
| 18-Mar-21 | 35.4 | 23.5 | 0 | 4 | C | S | 1 | 0 | 70 | 40 | 8.7 | 9.2 |
| 19-Mar-21 | 36.6 | 22.2 | 0 | 3.5 | S | NW | 0 | 0 | 82 | 33 | 9.5 | 9.6 |
| 20-Mar-21 | 32.6 | 21.8 | 0 | 4.5 | S | SE | 1 | 5.0 | 84 | 59 | 3.2 | 9.4 |
| 21-Mar-21 | 31 | 22 | 3.5 | 3.4 | C | N | 5 | 6 | 91 | 94 | 0 | 9 |
| 22-Mar-21 | 24 | 19.6 | 167.1 | 7.9 | NW | NW | 2 | 1 | 83 | 67 | 0 | 8 |
| 23-Mar-21 | 30.8 | 20 | 14.2 | 6.1 | NW | E | 6 | 0 | 94 | 61 | 1.4 | 8.4 |
| 24-Mar-21 | 31 | 19.5 | 33.5 | 6.7 | E | N | 0 | 0 | 81 | 63 | 7.9 | 8.2 |
| 25-Mar-21 | 33.8 | 22.2 | 0 | 3.4 | W | W | 1 | 1 | 64 | 56 | 9.9 | 8.9 |
| 26-Mar-21 | 32.5 | 21.8 | 0 | 6 | NW | W | 0 | 0 | 70 | 47 | 2.7 | 8 |
| 27-Mar-21 | 35.5 | 22.8 | 0 | 6.9 | NW | NW | 0 | 0 | 60 | 43 | 9.5 | 8.6 |
| 28-Mar-21 | 38.5 | 21.6 | 0 | 4.3 | NW | NW | 0 | 0 | 62 | 38 | 10.3 | 8.9 |
| 29-Mar-21 | 40.8 | 22 | 0 | 4.3 | NW | W | 0 | 0 | 66 | 34 | 10.5 | 8.2 |
| 30-Mar-21 | 40.5 | 26 | 0 | 5.3 | S | S | 0 | 0 | 79 | 33 | 10.9 | 8.8 |
| 31-Mar-21 | 39 | 27.5 | 0 | 6.2 | S | S | 1 | 0 | 64 | 47 | 10.3 | 8.9 |
| 1-Apr-21 | 36.4 | 26.2 | 0 | 10.5 | E | S | 1 | 0 | 80 | 56 | 8.5 | 8.6 |
| 2-Apr-21 | 36.8 | 25.4 | 0 | 4.8 | C | SE | 1 | 0 | 63 | 46 | 8.8 | 8.4 |
| 3-Apr-21 | 38.7 | 20.2 | 7.8 | 6.9 | E | E | 0 | 1 | 74 | 61 | 2.9 | 7.5 |
| 4-Apr-21 | 31.8 | 23.2 | 0 | 3.7 | C | S | 1 | 1 | 74 | 52 | 5.6 | 8 |
| 5-Apr-21 | 36.2 | 25.2 | 0 | 6 | C | S | 0 | 0 | 78 | 47 | 6.3 | 8.5 |
| 6-Apr-21 | 37 | 27 | 0 | 2.6 | C | SE | 1 | 0 | 75 | 59 | 6.5 | 8.7 |
| 7-Apr-21 | 38.2 | 20 | 18.2 | 4.6 | NE | W | 0 | 0 | 74 | 41 | 2.1 | 6.9 |
| 8-Apr-21 | 37 | 20.1 | 0 | 4.8 | C | W | 0 | 0 | 67 | 41 | 1.8 | 5.8 |
| 9-Apr-21 | 38 | 26.2 | 0 | 2.8 | SW | W | 0 | 1 | 74 | 34 | 2.9 | 5 |
| 10-Apr-21 | 39.6 | 29 | 0 | 5 | W | NW | 1 | 0 | 62 | 31 | 8.9 | 5.8 |
| 11-Apr-21 | 41.5 | 29.4 | 0 | 12.6 | W | NW | 1 | 0 | 60 | 35 | 7.1 | 6.7 |
| 12-Apr-21 | 41.5 | 29.6 | 0 | 4.4 | S | SE | 1 | 1 | 76 | 52 | 6.8 | 6.8 |
| 13-Apr-21 | 38.2 | 23.4 | 5.8 | 7.3 | C | E | 1 | 1 | 75 | 57 | 5.2 | 5.8 |
| 14-Apr-21 | 38 | 28.8 | 0 | 1.6 | SE | SE | 1 | 1 | 77 | 65 | 5.7 | 5 |
| 15-Apr-21 | 34.4 | 24.4 | 0 | 12.7 | SE | NW | 0 | 1 | 71 | 78 | 2 | 5.6 |
| 16-Apr-21 | 33.8 | 26.2 | 0 | 4.6 | SW | NW | 0 | 0 | 76 | 55 | 5.6 | 4.6 |
| 17-Apr-21 | 37.5 | 26 | 0 | 3.9 | SE | W | 1 | 1 | 71 | 58 | 8.1 | 5.2 |
| 18-Apr-21 | 38 | 26.8 | 0 | 6.4 | SE | NW | 1 | 1 | 71 | 85 | 5.1 | 5.8 |
| 19-Apr-21 | 33 | 26.8 | 0.3 | 4.3 | C | NW | 0 | 0 | 78 | 72 | 3.7 | 4.3 |
| 20-Apr-21 | 33.5 | 25.4 | 18 | 4.5 | C |  | 0 |  | 83 |  | 4.9 | 4 |
| **Year 2021-2022** | | | | | | | | | | | | |
| 1-Oct-20 | 29 | 12.4 | 0 | 2.4 | C | S | 0 | 1 | 93 | 38 | 8 | 3.8 |
| 2-Oct-20 | 30.9 | 13.9 | 0 | 1.5 | C | S | 0 | 1 | 89 | 57 | 7.6 | 3.7 |
| 3-Oct-20 | 29 | 15.1 | 0 | 2.3 | C | S | 1 | 0 | 94 | 58 | 6.6 | 2.8 |
| 4-Oct-20 | 28.9 | 14.1 | 0 | 1.3 | C | C | 4 | 1 | 98 | 58 | 4.8 | 2.5 |
| 5-Oct-20 | 27 | 12.4 | 0 | 1.4 | C | NW | 4 | 4.0 | 96 | 79 | 0 | 2.6 |
| 6-Oct-20 | 26.5 | 12.4 | 0 | 1.3 | C | W | 4 | 1 | 91 | 35 | 0 | 2.3 |
| 7-Oct-20 | 28 | 12.6 | 0 | 2.7 | C | C | 4 | 1 | 91 | 41 | 1.8 | 2 |
| 8-Oct-20 | 28.9 | 12 | 0 | 1.8 | C | NW | 4 | 0 | 93 | 45 | 5.5 | 2.4 |
| 9-Oct-20 | 29 | 12 | 0 | 1.9 | C | NW | 4 | 0 | 95 | 34 | 5.2 | 2.5 |
| 10-Oct-20 | 28.2 | 11.2 | 0 | 1.9 | C | NW | 4 | 0 | 93 | 51 | 3.2 | 2.4 |
| 11-Oct-20 | 29 | 11.4 | 0 | 1.1 | C | NW | 4 | 1 | 95 | 67 | 4.2 | 2.5 |
| 12-Oct-20 | 25.5 | 11 | 0 | 0.9 | C | N | 4 | 0 | 93 | 45 | 0 | 1.8 |
| 13-Oct-20 | 27.6 | 9.6 | 0 | 1.3 | C | NW | 4 | 0 | 93 | 43 | 1.3 | 2.3 |
| 14-Oct-20 | 27.5 | 9 | 0 | 1.7 | C | NW | 4 | 0 | 88 | 46 | 5 | 2.6 |
| 15-Oct-20 | 27 | 9 | 0 | 0.8 | C | W | 4 | 1 | 90 | 62 | 5.8 | 2.3 |
| 16-Oct-20 | 25.6 | 8.6 | 0 | 0.8 | C | W | 4 | 1 | 92 | 34 | 3 | 1.9 |
| 17-Oct-20 | 26 | 8.5 | 0 | 1.6 | C | NE | 4 | 0 | 90 | 64 | 5.2 | 2.4 |
| 18-Oct-20 | 25.2 | 9.1 | 0 | 1 | C | W | 4 | 1 | 90 | 60 | 5.4 | 2.7 |
| 19-Oct-20 | 27 | 9.4 | 0 | 0.5 | C | W | 4 | 1 | 88 | 63 | 4.6 | 2.4 |
| 20-Oct-20 | 25 | 13.2 | 0 | 0.9 | C | N | 4 | 1 | 93 | 63 | 3.3 | 1.9 |
| 21-Oct-20 | 26 | 9.4 | 0 | 1 | C | W | 4 | 0 | 95 | 46 | 4.6 | 2 |
| 22-Oct-20 | 27 | 10.2 | 0 | 3.3 | W | NW | 0 | 0 | 76 | 52 | 6.8 | 3 |
| 23-Oct-20 | 27 | 8.6 | 0 | 5 | C | W | 0 | 0 | 83 | 53 | 8 | 3.8 |
| 24-Oct-20 | 25.4 | 7.4 | 0 | 2 | C | C | 4 | 0 | 92 | 64 | 7 | 2.2 |
| 25-Oct-20 | 28 | 9 | 0 | 0.6 | C | S | 4 | 1 | 90 | 58 | 6.6 | 2.6 |
| 26-Oct-20 | 26.8 | 9 | 0 | 1.1 | C | S | 4 | 0 | 92 | 57 | 4.5 | 1.9 |
| 27-Oct-20 | 26 | 9 | 0 | 1 | C | E | 4 | 1 | 90 | 51 | 4.4 | 2.3 |
| 28-Oct-20 | 25.2 | 10.4 | 0 | 1.6 | C | C | 4 | 1 | 93 | 51 | 4.5 | 2.3 |
| 29-Oct-20 | 25.7 | 8.7 | 0 | 0.9 | C | W | 4 | 0.0 | 97 | 40 | 2.7 | 2.1 |
| 30-Oct-20 | 25.6 | 9 | 0 | 2 | C | W | 4 | 0 | 95 | 44 | 6.1 | 2 |
| 31-Oct-20 | 25.9 | 10.9 | 0 | 2.1 | C | E | 4 | 4 | 88 | 64 | 5.6 | 1.8 |
| 1-Nov-20 | 22.4 | 13 | 0 | 0.4 | C | W | 4 | 4 | 93 | 72 | 0 | 1.5 |
| 2-Nov-20 | 20 | 11.4 | 2 | 1.7 | W | NW | 4 | 4 | 87 | 73 | 0 | 1.7 |
| 3-Nov-20 | 22 | 10 | 0 | 1.5 | C | W | 4 | 0 | 88 | 60 | 0 | 1.6 |
| 4-Nov-20 | 26.4 | 10.6 | 0 | 1.5 | C | SE | 4 | 1 | 88 | 67 | 7 | 2.9 |
| 5-Nov-20 | 25.4 | 14.2 | 0 | 1.3 | C | NW | 4 | 0 | 96 | 54 | 2.4 | 1.7 |
| 6-Nov-20 | 24.2 | 11 | 0 | 2.4 | C | NW | 4 | 0 | 91 | 60 | 3.8 | 2.2 |
| 7-Nov-20 | 24.2 | 8.2 | 0 | 2.9 | C | NW | 4 | 0.0 | 92 | 59 | 7.6 | 2.3 |
| 8-Nov-20 | 24 | 7 | 0 | 2.2 | C | NW | 4 | 1 | 89 | 73 | 7.6 | 2.4 |
| 9-Nov-20 | 23 | 7.6 | 0 | 1.3 | C |  | 4 |  | 87 |  | 4.2 | 1.7 |
| 10-Nov-20 | 21.2 | 9.7 | 0.0 | 3.0 | C | C | 4.0 | 0.0 | 97.0 | 54.0 | 0.0 | 1.4 |
| 11-Nov-20 | 22.9 | 7.7 | 0.0 | 1.6 | W | NW | 2.0 | 0.0 | 90.0 | 54.0 | 5.9 | 1.9 |
| 12-Nov-20 | 18.0 | 3.6 | 0.0 | 7.9 | NW | N | 4.0 | 1.0 | 92.0 | 59.0 | 6.7 | 1.6 |
| 13-Nov-20 | 18.0 | 16.2 | 0.0 | 7.3 | NW | SW | 4.0 | 0.0 | 97.0 | 52.0 | 3.7 | 1.3 |
| 14-Nov-20 | 17.4 | 3.4 | 0.0 | 2.8 | W | W | 4.0 | 0.0 | 84.0 | 57.0 | 5.6 | 1.6 |
| 15-Nov-20 | 14.6 | 4.6 | 0.0 | 5.6 | W | W | 4.0 | 0.0 | 74.0 | 38.0 | 6.0 | 1.7 |
| 16-Nov-20 | 19.4 | 2.5 | 0.0 | 4.1 | C | NW | 4.0 | 0.0 | 78.0 | 42.0 | 8.2 | 2.0 |
| 17-Nov-20 | 21.2 | 2.4 | 0.0 | 3.4 | C | W | 4.0 | 0.0 | 70.0 | 27.0 | 7.6 | 2.2 |
| 18-Nov-20 | 21.2 | 3.9 | 0.0 | 2.8 | C | W | 4.0 | 0.0 | 85.0 | 39.0 | 5.9 | 1.8 |
| 19-Nov-20 | 22.5 | 3.5 | 0.0 | 2.0 | C | NW | 4.0 | 0.0 | 94.0 | 36.0 | 5.9 | 2.3 |
| 20-Nov-20 | 22.6 | 2.6 | 0.0 | 1.2 | C | W | 4.0 | 0.0 | 93.0 | 43.0 | 5.5 | 2.0 |
| 21-Nov-20 | 23.0 | 2.9 | 0.0 | 1.2 | C | W | 4.0 | 0.0 | 93.0 | 48.0 | 4.6 | 1.7 |
| 22-Nov-20 | 22.0 | 3.6 | 0.0 | 4.7 | C | NW | 4.0 | 0.0 | 94.0 | 34.0 | 2.9 | 1.7 |
| 23-Nov-20 | 19.0 | 3.5 | 0.0 | 3.6 | C | NW | 4.0 | 0.0 | 85.0 | 47.0 | 7.5 | 2.0 |
| 24-Nov-20 | 17.0 | 5.0 | 0.0 | 6.0 | NW | W | 4.0 | 0.0 | 94.0 | 64.0 | 7.2 | 1.6 |
| 25-Nov-20 | 16.0 | 2.6 | 0.0 | 4.2 | NW | W | 4.0 | 1.0 | 97.0 | 63.0 | 5.5 | 1.5 |
| 26-Nov-20 | 18.2 | 3.6 | 0.0 | 1.3 | C | NW | 4.0 | 0.0 | 93.0 | 42.0 | 5.2 | 1.5 |
| 27-Nov-20 | 17.3 | 3.2 | 0.0 | 3.0 | C | NW | 4.0 | 0.0 | 90.0 | 48.0 | 6.6 | 1.6 |
| 28-Nov-20 | 19.2 | 2.6 | 0.0 | 2.0 | C | SE | 4.0 | 1.0 | 88.0 | 55.0 | 6.1 | 5.1 |
| 29-Nov-20 | 18.6 | 2.9 | 0 | 1.5 | C | C | 4 | 0 | 87 | 78 | 5.1 | 1.8 |
| 30-Nov-20 | 20 | 3.6 | 0 | 1.3 | C | NW | 4 | 0 | 94 | 68 | 3.8 | 1.5 |
| 1-Dec-20 | 20 | 4 | 0 | 1.3 | C | W | 4 | 1 | 97 | 69 | 4 | 1.7 |
| 2-Dec-20 | 21 | 6.5 | 0 | 1.9 | C | W | 4 | 1 | 94 | 53 | 5.8 | 2 |
| 3-Dec-20 | 23.4 | 9.5 | 0 | 1.2 | C | SE | 4 | 6 | 95 | 95 | 3 | 1.8 |
| 4-Dec-20 | 16 | 11.4 | 11.8 | 3.4 | C | SE | 4 | 1 | 91 | 81 | 0 | 1.1 |
| 5-Dec-20 | 20 | 12.4 | 0 | 4.9 | S | SE | 4 | 1 | 93 | 90 | 1 | 1.5 |
| 6-Dec-20 | 19.6 | 14.4 | 69.2 | 6.4 | E | E | 6 | 1 | 93 | 96 | 1 | 1.8 |
| 7-Dec-20 | 17 | 13.4 | 26.2 | 5 | W | W | 6 | 1 | 93 | 84 | 0 | 1.4 |
| 8-Dec-20 | 15.2 | 9 | 1.4 | 4.3 | NW | W | 4 | 1 | 92 | 79 | 0 | 1 |
| 9-Dec-20 | 16.8 | 5.4 | 0 | 1.8 | C | W | 4 | 1 | 97 | 74 | 0.7 | 1.5 |
| 10-Dec-20 | 17 | 7.7 | 0 | 6.7 | C | W | 4 | 1 | 92 | 64 | 6.7 | 1.9 |
| 11-Dec-20 | 17.4 | 4.9 | 0 | 1.6 | C | C | 4 | 1 | 94 | 71 | 2.3 | 1.6 |
| 12-Dec-20 | 16.4 | 5 | 0 | 0.9 | C | N | 4 | 4 | 92 | 66 | 0 | 1.7 |
| 13-Dec-20 | 14.5 | 6.4 | 0 | 2.9 | W | S | 4 | 4 | 92 | 69 | 0 | 1.6 |
| 14-Dec-20 | 15.5 | 7 | 0 | 3.5 | C | N | 4 | 1 | 91 | 67 | 0 | 1.1 |
| 15-Dec-20 | 15.4 | 7.4 | 0 | 2.2 | C | W | 4 | 1 | 81 | 60 | 3.3 | 1.6 |
| 16-Dec-20 | 15.4 | 8.4 | 0 | 2.5 | C | NW | 4 | 1 | 94 | 76 | 1.7 | 1.4 |
| 17-Dec-20 | 14.4 | 7 | 0 | 3.6 | NW | NW | 4 | 1 | 92 | 85 | 1.5 | 1.7 |
| 18-Dec-20 | 16.4 | 9.2 | 0 | 1.8 | SE | SE | 4 | 1 | 97 | 84 | 1.5 | 1.6 |
| 19-Dec-20 | 18 | 6.9 | 0 | 2.2 | C | SE | 4 | 1 | 97 | 75 | 0.3 | 1.8 |
| 20-Dec-20 | 19.2 | 10.5 | 7 | 4 | SE | SE | 6 | 1 | 93 | 89 | 1.3 | 1.7 |
| 21-Dec-20 | 15 | 10 | 26.3 | 7.6 | C | NW | 4 | 4 | 93 | 82 | 0 | 1.3 |
| 22-Dec-20 | 15.2 | 7 | 0 | 1.9 | C | C | 4 | 2 | 87 | 82 | 0 | 1.2 |
| 23-Dec-20 | 14.4 | 5 | 0 | 1.5 | W | NW | 4 | 2 | 92 | 92 | 0 | 1 |
| 24-Dec-20 | 11.6 | 9.2 | 0 | 3.3 | C | NW | 4 | 1 | 100 | 80 | 0 | 1 |
| 25-Dec-20 | 15.2 | 4 | 0 | 3 | W | NW | 4 | 1 | 91 | 74 | 0.8 | 1 |
| 26-Dec-20 | 18 | 6.2 | 0 | 4.4 | C | W | 4 | 0 | 97 | 69 | 7.2 | 1.6 |
| 27-Dec-20 | 19.6 | 4 | 0 | 4.6 | C | W | 4 | 0 | 83 | 47 | 8.6 | 2 |
| 28-Dec-20 | 21.2 | 7.5 | 0 | 3.8 | W | W | 4 | 0 | 85 | 68 | 8.4 | 2.5 |
| 29-Dec-20 | 21.2 | 5 | 0 | 4.9 | C | NNE | 4 | 1 | 91 | 74 | 7.8 | 2.7 |
| 30-Dec-20 | 21.5 | 5 | 0 | 2 | C | W | 4 | 1 | 94 | 75 | 3.3 | 1.8 |
| 31-Dec-20 | 19.2 | 10 | 0 | 5.4 | W | SSW | 4 | 1 | 93 | 72 | 4.7 | 1.7 |
| 1-Jan-21 | 22 | 10.4 | 0 | 6.9 | SE | SE | 4 | 4 | 93 | 86 | 5.8 | 1.6 |
| 2-Jan-21 | 14 | 9.6 | 1.8 | 9.5 | W | W | 4 | 1 | 93 | 79 | 0 | 1.4 |
| 3-Jan-21 | 17.5 | 4.5 | 0 | 3.5 | C | W | 4 | 1 | 94 | 78 | 3.3 | 1.6 |
| 4-Jan-21 | 18 | 3.8 | 0 | 1.5 | C | NW | 4 | 0 | 84 | 65 | 6 | 1.9 |
| 5-Jan-21 | 22.4 | 7.6 | 0 | 1.3 | C | W | 4 | 0 | 88 | 68 | 7.9 | 2.5 |
| 6-Jan-21 | 25 | 10 | 0 | 1.6 | C | E | 4 | 1 | 95 | 80 | 6.8 | 3 |
| 7-Jan-21 | 24 | 10 | 10.8 | 2.8 | N | E | 6 | 1 | 98 | 72 | 4.1 | 1.8 |
| 8-Jan-21 | 20 | 8 | 0 | 2.9 | C | WNW | 4 | 1 | 95 | 52 | 3.8 | 2 |
| 9-Jan-21 | 21 | 8.5 | 0 | 4.8 | W | W | 4 | 0 | 83 | 42 | 7.6 | 2.6 |
| 10-Jan-21 | 22 | 5.5 | 0 | 5.6 | SW | NW | 0 | 0 | 82 | 35 | 8.8 | 3 |
| 11-Jan-21 | 23 | 5.4 | 0 | 4.1 | C | NW | 4 | 0 | 86 | 52 | 8.8 | 3.2 |
| 12-Jan-21 | 25.2 | 7 | 0 | 1.2 | C | W | 0 |  | 86 |  | 8.8 | 3.7 |

**NOTE:**

1. **SOURCE:** Agromet Observatory, Division of Agricultural Physics, IARI, New Delhi (Latitude 28°38'23"N, Longitude:77°09'27"E., Altitude:228.61m above). https://iari.res.in/bms/daily-weather/
2. ABBREVIATIONS: C=Calm / No wind, N=North, S= South, E=East, W= West, NW=North West, NE=North East, SW=South West, SE=South East, WNW= West North-West, SSW=South south-west, NNE=North-northeast, RH=Relative Humidity, BSS=Bright Sun Shine hours
3. Observations taken twice at 7**.21** hrs. and **14:21** hrs, known as Observations I & II.
4. **Code:0** - Fair (clear and slightly clouded), **1** - Variable Sky, **2** - Mainly Overcast, **3** - Sand Storm or Dust Storm, **4** - Fog or Thick Dust Haze, **5** - Drizzle, **6** - Rain, **7** - Snow or Sleet, **8** - Shower (s), **9** - Thunder Storm with or without Precipitation
5. Maximum temperature observed during previous day, Minimum temperature observed on observed day, Maximum Rainfall observed during previous day 8:30 AM and Minimum on observed day 8:30 AM, Wind speed observed average during previous day 7:21 AM to observed day 7:21 AM, Wind direction I observed day at 7:21 AM, Wind direction II observed day at 2:21 PM, Weather condition I observed day at 7:21 AM, Weather condition II observed day at 2:21 PM, RH I observed day at 7:21 AM, RH II observed day at 2:21 PM, BSS observed during previous day, Evaporation observed during previous day.
6. Weather condition-I (Code 0-9) could represent the primary weather condition, such as clear skies, rain, snow, fog, etc. The numbers 0 through 9 would each correspond to a different weather condition.
7. Weather condition-II (Code 0-9) could represent secondary or additional weather conditions, such as intensity, visibility, wind speed, etc. Again, the numbers 0 through 9 would each correspond to a different condition.
