# Supplementary material for: Symbiotic nitrogen fixation for sustainable chickpea yield and prospects for genome editing in changing climatic situations
Source: Front Plant Sci. 2025 Sep 1;16:1621191. doi: 10.3389/fpls.2025.1621191 (PMC12433944; doi:10.3389/fpls.2025.1621191)
Supplement: Supplementary file 5 [file Table3.docx]

**SUPPLEMENTARY TABLE 3: ANOVA FOR 20 GENOTYPES, 15 TRAITS, 8 TREATMENTS (ENVIRONMENTS)**

| **Interactions**  **/ D.F. →**  **Traits**  **↓** | **Replicates** | **Year** | **Treatment** | **Year x Treatment** | **Genotype** | **Year x Genotype** | **Treatment x Genotype** | **Year x Treatment x Genotype** | **Error C** | **C.V. %** |
| --- | --- | --- | --- | --- | --- | --- | --- | --- | --- | --- |
|  | **2** | **1** | **7** | **7** | **19** | **19** | **133** | **133** | **638** | **1** |
| **Number of Nodules** | 0.84 | 10113.77*** | 1864.25*** | 800.19 *** | 1805.44 *** | 197.27 *** | 136.73 *** | 149.97 ** * | 2.00 | 7.99 |
| **Nodule fresh weight (mg)** | 901.12 | 8 65701.50*** | 518769.40*** | 411783.40*** | 322235.30*** | 267685.50*** | 118496.20*** | 93224.37*** | 763.61 | 6.90 |
| **Seed weight per plant (g)** | 0.02 * | 1 54.93* ** | 4.11*** | 3.97*** | 2.20** * | 1.80** * | 0.65** * | 0.62** * | 0.00 | 6.43 |
| **Nodule d weight (mg)** | 102.2 | 48 53460.00*** | 116774.70 *** | 101632.50 *** | 39176.50 *** | 38221.74 *** | 12407.27 *** | 11107.92 *** | 68.67 | 8.23 |
| **Root fresh weight (g)** | 0.03 | 125.47 *** | 24.15 *** | 27.02 *** | 6.19 *** | 6.21 *** | 3.76 *** | 4.04 *** | 0.02 | 7.18 |
| **Root dry weight (g)** | 0.00 | 4.01 *** | 0.77 *** | 0.87 *** | 0.20 *** | 0.20 *** | 0.12 *** | 0.13 *** | 0.00 | 6.78 |
| **Shoot fresh weight (g)** | 0.48 | 230.71 *** | 309.49 *** | 251.29 *** | 71.46 *** | 73.84 *** | 23.33 *** | 19.42 *** | 0.29 | 6.58 |
| **Shoot dry weight (g)** | 0.00 | 12.24 ** * | 16.34 *** | 13.27 *** | 3.76 *** | 3.90 *** | 1.23 *** | 1.02 *** | 0.01 | 6.33 |
| **Plant height (cm)** | 0.56 | 23474.53 *** | 259.68 *** | 200.40 *** | 70.85 *** | 78.94 *** | 52.62 *** | 41.24 *** | 1.10 | 5.74 |
| **Number of primary branches** | 0.02 | 0.23 *** | 3.66 *** | 0.21 *** | 1.20 *** | 0.11*** | 0.51 *** | 0.18 *** | 0.02 | 5.90 |
| **Number of secondary branches** | 1.83 | 667.36** * | 960.71 ** * | 67.98 *** | 123.23 *** | 8.64 *** | 46.97 *** | 17.80 *** | 0.74 | 6.35 |
| **Days to flowering** | 10.86 | 12333.31*** | 1091.29 ** * | 840.30 ** * | 36.84 | 49.78 ** | 24.81 | 39.88 *** | 24.05 | 6.12 |
| **Days to maturity** | 45.10 | 2 28424.80*** | 8940.32*** | 7711.05*** | 47.30 | 117.55*** | 58.83* | 76.24*** | 45.59 | 6.06 |
| **Number of pods per plant** | 0.94 | 310.13*** | 264.49*** | 301.30*** | 87.74*** | 175.64*** | 79.20*** | 63.17*** | 0.89 | 6.54 |
| **Number of seeds per plant** | 2.02 | 694.70 *** | 311.01 *** | 260.98 *** | 129.65 *** | 313.57 *** | 80.97 *** | 74.30 *** | 1.00 | 6.70 |

**Note:** 1- Asterisks (*) indicate as *=Significant @ 5%, **=Significance @ 1%, ***=Significance @ 0.1%

2- **Abbreviations:** DF= Degree of Freedom, MSS= Mean sum of squares, CV= Coefficient of Variation

**SUPPLEMENTARY TABLE 4: GENETIC PARAMETERS FOR VARIOUS TRAITS**

| **Traits** | **GCV (%)** | **PCV**  **(%)** | **h^2^ (BS)**  **(%)** | **GA @ 5%** | **GA as % of mean @ 5%** |
| --- | --- | --- | --- | --- | --- |
| **Number of secondary branches** | 23.1 | 27.0 | 73.6 | 3.9 | 40.9 |
| **Number of primary branches** | 14.6 | 17.2 | 72.6 | 0.7 | 25.6 |
| **Number of Nodules** | 37.4 | 45.0 | 68.9 | 9.0 | 63.9 |
| **Plant height** | 29.9 | 39.2 | 58.2 | 8.2 | 47.0 |
| **Days to maturity** | 0.6 | 6.9 | 58.2 | 10.4 | 9.7 |
| **Seed weight per plant** | 29.7 | 41.5 | 51.3 | 0.5 | 43.8 |
| **Number of pods/plants** | 24.9 | 34.9 | 50.8 | 4.7 | 36.6 |
| **Root fresh weight** | 53.8 | 87.3 | 38.0 | 1.5 | 68.3 |
| **Root dry weight** | 54.0 | 87.7 | 37.9 | 0.3 | 68.5 |
| **Nodule dry weight** | 63.0 | 103.5 | 37.1 | 63.6 | 79.1 |
| **Shoot fresh weight** | 26.6 | 43.6 | 37.1 | 2.9 | 33.3 |
| **Shoot dry weight** | 26.6 | 43.7 | 37.0 | 0.7 | 33.3 |
| **Number of seeds/plants** | 30.4 | 50.5 | 36.3 | 5.2 | 37.8 |
| **Nodule fresh weight** | 30.1 | 57.8 | 27.1 | 107.8 | 32.3 |
| **Days to flowering** | 2.1 | 7.0 | 9.0 | 10.2 | 10.3 |

**Abrreviations**: GCV = Genotypic Coefficient of Variation, PCV= Phenotypic Coefficient of Variation,

h^2^ (BS) = Heritability in broad sense, GA= Genetic Advancement

**SUPPLEMENTARY TABLE 5: GENOTYPIC & PHENOTYPIC CORRELATION MATRES FOR VARIOUS TRAITS**

| **Traits →**  **↓** | **Nodule fresh weight** | **Seed weight per plant** | **Nodule dry weight** | **Root fresh weight** | **Root dry weight** | **Shoot fresh weight** | **Shoot dry weight** | **Plant height (cm)** | **Number of primary branches** | **Number of secondary branches** | **Days to flowering** | **Days to maturity** | **Number of pods per plant** | **Number of seeds per plant** | **Number of Nodules** |
| --- | --- | --- | --- | --- | --- | --- | --- | --- | --- | --- | --- | --- | --- | --- | --- |
| **Nodule fresh weight** | 1 | 0.13 | 0.84** | 0.14 | 0.14 | 0.74** | 0.74** | 0.91** | 0.26* | 0.49** | -0.03 | -0.56** | 0.42** | -0.33* | 0.63** |
| **Seed weight per plant** | 0.03 | 1 | 0.23 | -0.44** | -0.44** | 0.27* | 0.27* | 0.19 | 0.36* | 0.2 | 0.07 | -0.61** | 0.42** | 0.67** | 0.43** |
| **Nodule dry weight** | 0.86** | 0.21 | 1 | -0.17 | -0.17 | 0.49** | 0.49** | 0.82** | 0.15 | 0.62** | -0.12 | -0.41** | 0.51** | -0.23 | 0.52** |
| **Root fresh weight** | 0.40* | -0.31* | 0.09 | 1 | 0.88** | 0.29* | 0.29* | 0.05 | -0.38* | -0.15 | 0.21 | 0.82** | -0.31* | -0.35* | -0.09 |
| **Root dry weight** | 0.41* | -0.31* | 0.09 | 0.98** | 1 | 0.29* | 0.29* | 0.04 | -0.38* | -0.16 | 0.18 | 0.92** | -0.32* | -0.35* | -0.09 |
| **Shoot fresh weight** | 0.51** | 0.35* | 0.38* | 0.41* | 0.42** | 1 | 0.86** | 0.46** | -0.01 | 0.38* | 0.30* | 0.05 | 0.24 | 0.25* | 0.46** |
| **Shoot dry weight** | 0.52** | 0.35* | 0.37* | 0.41** | 0.41** | 0.97** | 1 | 0.46** | -0.01 | 0.38* | 0.31* | 0.15 | 0.24 | 0.25* | 0.46** |
| **Plant height** | 0.13 | 0.16 | 0.26* | -0.03 | -0.03 | 0.33* | 0.33* | 1 | -0.03 | 0.50** | 0.21 | -0.09 | 0.30* | 0.04 | 0.16 |
| **Number of primary branches** | 0.23 | 0.16 | 0.16 | -0.11 | -0.12 | -0.04 | -0.04 | -0.18 | 1 | 0.19 | -0.35* | -0.80** | 0.07 | 0.13 | 0.26* |
| **Number of secondary branches** | 0.2 | 0.07 | 0.25 | -0.04 | -0.04 | 0.32* | 0.32* | 0.47** | 0.06 | 1 | 0.02 | -0.45** | 0.01 | 0.13 | 0.39* |
| **Days to flowering** | 0.06 | 0.06 | 0.07 | -0.04 | -0.07 | 0.03 | 0.03 | 0.02 | 0.08 | 0 | 1 | 0.91** | -0.2 | 0.39* | -0.22 |
| **Days to maturity** | 0.17 | 0.16 | 0.18 | -0.06 | -0.06 | 0.18 | 0.21 | 0.18 | 0.06 | 0.13 | 0.28* | 1 | -0.05 | 0.92** | -0.91** |
| **Number of pods/plant** | 0.17 | 0.284* | 0.306* | -0.255* | -0.25 | 0.19 | 0.19 | 0.18 | 0.05 | -0.05 | 0.07 | -0.04 | 1 | 0.12 | 0.46** |
| **Number of seeds/plant** | -0.06 | 0.69** | 0.11 | -0.22 | -0.22 | 0.32* | 0.31* | 0.05 | 0 | 0.02 | -0.08 | 0.06 | 0.2 | 1 | -0.06 |
| **Number of Nodules** | 0.42** | 0.2 | 0.40* | 0.02 | 0.03 | 0.287* | 0.28* | 0.07 | 0.22 | 0.23 | 0.03 | 0.1 | 0.44** | 0.04 | 1 |

**Note:** **1**- Asterisks (*) indicate as *=Significant @ 5%, ** = Significance @ 1%

**2-** Upper diagonal values are phenotypic and lower diagonal values are genotypic correlation coefficients

**SUPPLEMENTARY TABLE 6 (A): PHENOTYPIC PATH MATRIX FOR NUMBER OF NODULES**

| **Traits→**  **↓** | **Nodule fresh weight** | **Seed weight per plant** | **Nodule dry weight** | **Root fresh weight** | **Root dry weight** | **Shoot fresh weight** | **Shoot dry weight** | **Plant height** | **Number of primary branches** | **Number of secondary branches** | **Days to flowering** | **Days to maturity** | **Number of pods per plant** | **Number of seeds per plant** | **Number of Nodules**  **(rg)** |
| --- | --- | --- | --- | --- | --- | --- | --- | --- | --- | --- | --- | --- | --- | --- | --- |
| **Nodule fresh weight** | **0 .47** | 0.01 | 0.41 | 0.19 | 0.20 | 0.25 | 0.25 | 0.06 | 0.11 | 0.10 | 0.03 | 0.08 | 0.08 | -0.03 | 0.42** |
| **Seed weight per plant** | 0.01 | **0.23** | 0.05 | -0.07 | -0.07 | 0.08 | 0.08 | 0.04 | 0.04 | 0.02 | 0.01 | 0.04 | 0.07 | 0.16 | 0.20 |
| **Nodule dry weight** | -0.18 | -0.04 | **-0.21** | -0.02 | -0.02 | -0.08 | -0.08 | -0.06 | -0.03 | -0.05 | -0.01 | -0.04 | -0.06 | -0.02 | 0.40* |
| **Root fresh weight** | 0.05 | -0.04 | 0.01 | **0.11** | 0.11 | 0.05 | 0.05 | 0.00 | -0.01 | 0.00 | -0.01 | -0.01 | -0.03 | -0.03 | 0.02 |
| **Root dry weight** | -0.02 | 0.01 | 0.00 | -0.04 | **-0.04** | -0.02 | -0.02 | 0.00 | 0.01 | 0.00 | 0.00 | 0.00 | 0.01 | 0.01 | 0.03 |
| **Shoot fresh weight** | 0.04 | 0.03 | 0.03 | 0.03 | 0.03 | **0.07** | 0.07 | 0.03 | 0.00 | 0.02 | 0.00 | 0.01 | 0.01 | 0.02 | 0.28* |
| **Shoot dry weight** | -0.09 | -0.06 | -0.06 | -0.07 | -0.07 | -0.17 | **-0.17** | -0.06 | 0.01 | -0.05 | 0.00 | -0.03 | -0.03 | -0.05 | 0.28* |
| **Plant height** | -0.02 | -0.03 | -0.04 | 0.01 | 0.00 | -0.06 | -0.06 | -0.17 | 0.03 | -0.08 | 0.00 | -0.03 | -0.03 | -0.01 | 0.07 |
| **Number of primary branches** | 0.01 | 0.01 | 0.01 | 0.00 | 0.00 | 0.00 | 0.00 | -0.01 | **0.04** | 0.00 | 0.00 | 0.00 | 0.00 | 0.00 | 0.22 |
| **Number of secondary branches** | 0.06 | 0.02 | 0.07 | -0.01 | -0.01 | 0.10 | 0.09 | 0.14 | 0.02 | **0.29** | 0.00 | 0.04 | -0.02 | 0.01 | 0.23 |
| **Days to flowering** | 0.00 | 0.00 | 0.00 | 0.00 | 0.00 | 0.00 | 0.00 | 0.00 | -0.01 | 0.00 | **-0.06** | -0.02 | 0.00 | 0.00 | 0.03 |
| **Days to maturity** | 0.01 | 0.01 | 0.01 | 0.00 | 0.00 | 0.01 | 0.02 | 0.01 | 0.00 | 0.01 | 0.02 | **0.08** | 0.00 | 0.00 | 0.10 |
| **Number of pods per plant** | 0.08 | 0.14 | 0.15 | -0.12 | -0.12 | 0.09 | 0.09 | 0.08 | 0.02 | -0.03 | 0.04 | -0.02 | **0.48** | 0.09 | 0.44** |
| **Number of seeds per plant** | 0.01 | -0.09 | -0.01 | 0.03 | 0.03 | -0.04 | -0.04 | -0.01 | 0.00 | 0.00 | 0.01 | -0.01 | -0.03 | **-0.13** | 0.04 |
| **Number of Nodules (rg)** | 0.42** | 0.20 | 0.40* | 0.02 | 0.03 | 0.28* | 0.28* | 0.07 | 0.22 | 0.23 | 0.03 | 0.10 | 0.44** | 0.04 | 1 |
| **Partial R^2^** | 0.20 | 0.05 | -0.08 | 0.00 | 0.00 | 0.02 | -0.05 | -0.01 | 0.01 | 0.07 | 0.00 | 0.01 | 0.21 | 0.00 | - |

**Note:** 1- Asterisks (*) indicate as *Significant at 5%, ** Significance at 1%

2- Bold underlines indicate direct effects

**SUPPLEMENTARY TABLE 6 (B): GENOTYPIC PATH MATRIX FOR NUMBER OF NODULES**

| **Traits →**  **↓** | **Nodule fresh weight** | **Seed weight per plant** | **Nodule dry weight** | **Root fresh weight** | **Root dry weight** | **Shoot fresh weight** | **Shoot dry weight** | **Plant height** | **Number of primary branches** | **Number of secondary branches** | **Days to flowering** | **Days to maturity** | **Number of pods per plant** | **Number of seeds per plant** | **Number of Nodules (rg)** |
| --- | --- | --- | --- | --- | --- | --- | --- | --- | --- | --- | --- | --- | --- | --- | --- |
| **Nodule fresh weight** | **0.14** | 0.02 | 0.12 | 0.02 | 0.02 | 0.11 | 0.11 | 0.13 | 0.04 | 0.07 | 0.00 | -0.08 | 0.06 | -0.05 | 0.63** |
| **Seed weight per plant** | 0.10 | **0.70** | 0.16 | -0.31 | -0.31 | 0.19 | 0.19 | 0.14 | 0.26 | 0.14 | 0.05 | -0.71 | 0.29 | 0.47 | 0.43** |
| **Nodule dry weight** | -0.20 | -0.06 | **-0.23** | 0.04 | 0.04 | -0.12 | -0.12 | -0.26 | -0.04 | -0.15 | 0.03 | 0.33 | -0.12 | 0.06 | 0.52** |
| **Root fresh weight** | -0.01 | 0.03 | 0.01 | **-0.06** | -0.06 | -0.02 | -0.02 | 0.00 | 0.02 | 0.01 | -0.01 | -0.07 | 0.02 | 0.02 | -0.09 |
| **Root dry weight** | 0.00 | 0.00 | 0.00 | 0.00 | **0.00** | 0.00 | 0.00 | 0.00 | 0.00 | 0.00 | 0.00 | 0.00 | 0.00 | 0.00 | -0.09 |
| **Shoot fresh weight** | 0.19 | 0.07 | 0.13 | 0.08 | 0.08 | **0.26** | 0.26 | 0.12 | 0.00 | 0.10 | 0.08 | 0.01 | 0.06 | 0.07 | 0.46** |
| **Shoot dry weight** | 0.11 | 0.04 | 0.07 | 0.04 | 0.04 | 0.15 | **0.15** | 0.07 | 0.00 | 0.06 | 0.05 | 0.02 | 0.04 | 0.04 | 0.46** |
| **Plant height** | -0.26 | -0.06 | -0.31 | -0.01 | -0.01 | -0.13 | -0.13 | **-0.28** | 0.01 | -0.14 | -0.06 | 0.03 | -0.09 | -0.01 | 0.17 |
| **Number of primary branches** | -0.01 | -0.01 | 0.00 | 0.01 | 0.01 | 0.00 | 0.00 | 0.00 | **-0.02** | 0.00 | 0.01 | 0.04 | 0.00 | 0.00 | 0.26* |
| **Number of secondary branches** | 0.20 | 0.08 | 0.26 | -0.06 | -0.07 | 0.16 | 0.16 | 0.21 | 0.08 | **0.41** | 0.01 | -0.60 | 0.01 | 0.06 | 0.39* |
| **Days to flowering** | 0.00 | 0.00 | 0.00 | -0.01 | -0.01 | -0.01 | -0.01 | -0.01 | 0.01 | 0.00 | **-0.03** | -0.04 | 0.01 | -0.01 | -0.22 |
| **Days to maturity** | 0.00 | -0.01 | -0.01 | 0.01 | 0.01 | 0.00 | 0.00 | 0.00 | -0.01 | -0.01 | 0.01 | **0.00** | 0.00 | 0.01 | -0.91 |
| **Number of pods per plant** | 0.12 | 0.12 | 0.14 | -0.09 | -0.09 | 0.07 | 0.07 | 0.08 | 0.02 | 0.01 | -0.06 | -0.02 | **0.28** | 0.04 | 0.46** |
| **Number of seeds per plant** | 0.25 | -0.50 | 0.17 | 0.26 | 0.26 | -0.19 | -0.19 | -0.03 | -0.10 | -0.10 | -0.29 | -0.84 | -0.10 | **-0.74** | -0.06 |
| **Number of Nodules (rg)** | 0.63** | 0.43** | 0.52** | -0.10 | -0.10 | 0.46** | 0.46** | 0.17 | 0.26* | 0.392* | -0.22 | -0.91 | 0.463** | -0.06 | 1 |
| **Partial R^2^** | 0.09 | 0.31 | -0.12 | 0.01 | 0.00 | 0.12 | 0.07 | -0.05 | -0.01 | 0.16 | 0.01 | -0.01 | 0.13 | 0.05 | - |

**Note:** 1- Asterisks (*) indicate as *Significant at 5%, ** Significance at 1%, *** Significance at 0.1%

2- Bold underlines indicate direct effects

**SUPPLEMENTARY TABLE 7: CONTRIBUTIONs OF TRAITS TO DIVERGENCE**

| **S. No** | **Source** | **Contribution (%)** | **Times ranked 1st** |
| --- | --- | --- | --- |
| 1 | Number of Nodules | 15.38 | 29 |
| 2 | Nodule fresh weight (mg) | 8.52 | 16 |
| 3 | Seed weight per plant | 4.74 | 9 |
| 4 | Nodule dry weight (mg) | 0.53 | 1 |
| 5 | Root fresh weight (g) | 5.79 | 11 |
| 6 | Root dry weight (g) | 6.65 | 13 |
| 7 | Shoot fresh weight (g) | 5.23 | 10 |
| 8 | Shoot dry weight (g) | 6.62 | 12 |
| 9 | Plant height (cm) | 5.41 | 10 |
| 10 | Number of primary branches | 20 | 38 |
| 11 | Number of secondary branches | 3.65 | 7 |
| 12 | Days to flowering | 6.52 | 12 |
| 13 | Days to maturity | 0.53 | 1 |
| 14 | Number of pods per plant | 4.33 | 8 |
| 15 | Number of seeds per plant | 6.1 | 12 |

**SUPPLEMENTARY TABLE 8: PRINCIPAL COMPONENT ANALYSIS**

| **S. No** | **Genotypes** | **PCA I** | **PCA II** | **PCA III** |
| --- | --- | --- | --- | --- |
| 1 | **ICC 1083** | 5.37 | 1.58 | 5.07 |
| 2 | **ICC 1172** | 6.44 | 1.89 | 6.10 |
| 3 | **ICC3093** | 6.60 | 1.25 | 6.09 |
| 4 | **ICC3631** | 6.02 | 1.80 | 6.04 |
| 5 | **ICC6579** | 5.73 | 1.29 | 7.14 |
| 6 | **ICC6995** | 7.07 | 1.97 | 6.10 |
| 7 | **ICC7167** | 6.81 | 1.60 | 6.47 |
| 8 | **ICC7305** | 7.12 | 1.51 | 5.72 |
| 9 | **ICC13185** | 6.72 | 1.80 | 6.74 |
| 10 | **ICC1852** | 6.54 | 1.25 | 6.50 |
| 11 | **ICC1891** | 8.16 | 2.10 | 6.15 |
| 12 | **ICC1896** | 7.27 | 1.49 | 5.74 |
| 13 | **ICC2083** | 7.00 | 1.92 | 6.19 |
| 14 | **ICC3696** | 8.45 | 2.52 | 7.07 |
| 15 | **ICC4638** | 7.91 | 2.89 | 7.42 |
| 16 | **ICC6661** | 9.07 | 2.49 | 7.80 |
| 17 | **ICC9002** | 7.28 | 3.18 | 6.39 |
| 18 | **ICC9085** | 8.62 | 3.45 | 7.44 |
| 19 | **BG 372 (Check)** | 6.71 | 6.14 | 7.89 |
| 20 | **BG 3022 (Check)** | 9.13 | 3.19 | 6.20 |

**NOTE:** PCA I, II, III on the basis of X, Y and Z vectors

**SUPPLEMENTARY TABLE 9: NUMBER OF NODULES AND NITROGEN CONTENT FOR 20 GENOTYPES**

| **Treatments→**  **Genotypes↓** | **C** | | **VAM** | | **RZ** | |
| --- | --- | --- | --- | --- | --- | --- |
|  | **%N** | **NON** | **%N** | **NON** | **%N** | **NON** |
| **ICC 1083** | 1.69 | 1.566 | 1.99 | 5.734 | 2.89 | 4.23 |
| **ICC 1172** | 1.65 | 3.088 | 1.8 | 7.37 | 2.58 | 5.037 |
| **ICC3093** | 1.44 | 3.374 | 1.84 | 8.827 | 1.52 | 4.658 |
| **ICC3631** | 1.185 | 3.929 | 1.485 | 6.844 | 1.63 | 5.312 |
| **ICC6579** | 1.35 | 4.301 | 1.55 | 9.908 | 1.6 | 4.899 |
| **ICC6995** | 2.155 | 4.398 | 2.31 | 6.505 | 2.52 | 4.871 |
| **ICC7167** | 1.585 | 5.292 | 1.885 | 5.344 | 2.3 | 5.112 |
| **ICC7305** | 2.06 | 6.31 | 2.26 | 8.468 | 2.91 | 5.754 |
| **ICC13185** | 1.78 | 3.641 | 2.085 | 8.957 | 2.99 | 5.829 |
| **ICC1852** | 2.37 | 5.659 | 2.57 | 11.976 | 3.45 | 8.102 |
| **ICC1891** | 2.195 | 5.103 | 2.295 | 14.49 | 3.5 | 10.779 |
| **ICC1896** | 1.78 | 11.984 | 1.885 | 9.832 | 2.43 | 13.491 |
| **ICC2083** | 2.34 | 4.332 | 2.64 | 13.396 | 2.76 | 14.294 |
| **ICC3696** | 2.34 | 5.306 | 2.54 | 12.993 | 2.49 | 8.669 |
| **ICC4638** | 2.545 | 6.74 | 2.645 | 12.684 | 2.66 | 15.39 |
| **ICC6661** | 2.62 | 5.963 | 2.72 | 14.792 | 2.44 | 16.363 |
| **ICC9002** | 2.245 | 9.115 | 2.345 | 16.022 | 2.7 | 17.141 |
| **ICC9085** | 3.647 | 21.355 | 4.347 | 16.24 | 4.504 | 23.987 |
| **BG 372 (Check)** | 2.45 | 7.018 | 2.8 | 17.66 | 3.39 | 15.644 |
| **BG 3022 (Check)** | 2.515 | 12.125 | 2.865 | 15.921 | 3.24 | 17.655 |

**Note:** C= Control VAM= Vesicular Arbuscular Mycorrhiza, RZ= *Rhizobium*, N%= Nitrogen Percentage, NON= Number of Nodules
